# Pleiotropic Effect of Common Variants at ABO Glycosyltranferase Locus in 9q32 on Plasma Levels of Pancreatic Lipase and Angiotensin Converting Enzyme

**DOI:** 10.1371/journal.pone.0055903

**Published:** 2014-02-28

**Authors:** Yamagata University Genomic Cohort Consortium (YUGCC)

**Affiliations:** Children's National Medical Center, Washington, United States of America

## Abstract

For forty-three clinical test values presumably associated to common complex human diseases, we carried out a genome-wide association study using 600K SNPs in a general Japanese population of 1,639 individuals (1,252 after quality control procedures) drawn from a regional cohort, followed by a replication study for statistically significant SNPs (p = 1.95×10^−9^–8.34×10^−39^) using an independent population of 1,671 from another cohort. In this single two-stage study, we newly found strong and robust associations of common variants at the ABO histo-blood glycosyltransferase locus in 9q32 with the plasma levels of pancreatic lipase (P-LIP), in addition to successful confirmation of the known ABO association of angiotensin converting enzyme (ACE) independent of the ACE1 gene in 17q23.2 with the ACE level. Our results are compatible with the previously reported association between the ABO gene and pancreatic cancer, and show that the effect of these common variants at the ABO locus on the P-LIP and ACE levels is largely opposing and pleiotropic.

## Introduction

Genome-wide association studies using hundreds of thousands of single nucleotide polymorphisms (SNPs) have been revealing important genetic components underlying the common complex human diseases [1], even though their effect sizes are so modest or small as not to account for the original heritabilities of diseases [2]. In addition to such dichotomous traits, some quantitative characteristics such as body mass index (BMI), blood pressure or various kinds of clinical test values in general human populations are also attractive targets for genome-wide association study [3],[4] which are sometimes called as intermediate phenotype, endophenotype or biomarker presumably correlated to unobservable liability of diseases that has long been utilized as a theoretical tool to estimate diseases heritability [5]. With such quantitative endophenotypes underlying the common complex human diseases, association studies could be much more informative and powerful than with dichotomous traits themselves [6].

In order to identify genetic components affecting quantitative clinical test values, we carried out a population-based genome-wide association study and a subsequent replication study for the statistically significant SNPs beyond a genome-wide significance level (5×10^−8^) or the Bonferroni's corrected level by the number of phenotypes (5×10^−8^/43). For this two-stage design, we utilized two independent sample populations in Yamagata Prefecture located in the northeastern district of Japan; one from a regional cohort established in a small rural town, Takahata Town, for the 1st genome-wide genotyping, and another from a different cohort in the largest urban capital of the prefecture, Yamagata City, for the replication.

## Results

### Genome-wide genotyping in the 1st stage

**Figure 1:**
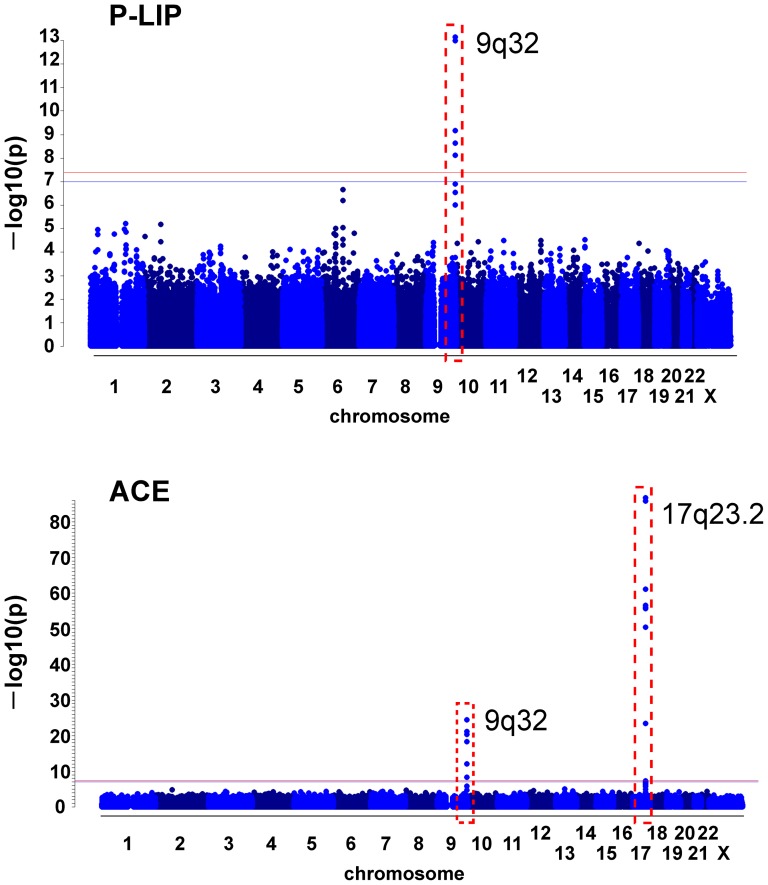
Manhattan plot for genome-wide association signals with the P-LIP (upper panel) and ACE (lower) levels in the Takahata population by linear regression with adjustment for age and gender as covariates. The red line represents a genome-wide significance level (5×10^−8^), whereas the blue line represents a genome-wide suggestive level (1×10^−7^). The red broken boxes indicate loci having the SNPs that attained a genome-wide significance level or the Bonferroni's corrected level by the number of phenotypes.

By applying standard quality control procedures (see the Methods for details) to the genome-wide genotyping data obtained using 600K SNP BeadChip (Illumina) in the Takahata population of 1,639 individuals, we eliminated low quality SNPs (i.e. low minor allele frequency, high missing rate or deviation from the Hardy-Weinberg equilibrium) and individuals with unusual statistics (i.e. high missing rate, high heterozygosity or cryptic relatedness) as well as potential population stratification [1], to have a high quality data set consisting of 436,670 SNPs in 1,252 individuals with 43 endophenotypic values (see a detailed list in the legend of Figure 5). By applying a standard linear regression analysis for each SNP in this data set with adjustment for (i.e. elimination of the potential confounding effect of) age and gender as covariates, we found strong associations of nine common variants at the ABO histo-blood glycosyltransferase locus in 9q32 with two endophenotypes, the plasma levels of P-LIP (Genomic inflation factor based on median chi-squared = 1.013) and ACE (1.011) (Figure 1), with extremely small p-values; rs4363269 (p = 1.50×10^−19^ for ACE), rs8176749 (5.30×10^−14^ for P-LIP; 1.00×10^−21^ for ACE), rs8176746 (3.89×10^−14^; 1.34×10^−22^), rs2073824 (4.00×10^−9^ for ACE), rs657152 (5.13×10^−10^ for P-LIP), rs500498 (6.26×10^−9^ for P-LIP), rs505922 (1.95×10^−9^ for P-LIP), rs495828 (4.27×10^−26^ for ACE) and rs7025162 (5.37×10^−13^ for ACE) as listed in Table 1. In addition to the ABO locus, we found that eight common variants at the ACE1 locus itself in 17q23.2 are also strongly associated with the ACE level; rs4459609 (p = 5.76×10^−56^), rs4309 (2.97×10^−69^), rs4311 (2.59×10^−62^), rs4329 (2.12×10^−63^), rs4343 (9.92×10^−63^), rs4353 (1×10^−102^), rs4362 (3.44×10^−104^) and rs4461142 (4.98×10^−25^) as listed in Table 2. Using this 1st data, we imputed unobserved variants on chromosome 9 based on data from 1000 Genomes project and test the effects of imputed variants on the ABO locus on the P-LIP and ACE levels (Tables S1 and S2 in File S1). These results show that there is no variant having lower p-value than that from the real data.

**Table 1:** Associations of ABO gene in 9q32 with P-LIP and ACE in Takahata and Yamagata populations.

SNPs (position)		minor		P-LIP				ACE			
	stage*	allele	MAF	beta**	SE	p value	ARS***	beta**	SE	p value	ARS***
rs4363269	1st	G	0.287	−0.0335	0.481	>1e-7	-	−1.96	0.213	1.50e-19	0.064
(136123840)	2nd	G	0.269	−0.242	0.511	>1e-7	-	−1.93	0.180	4.58e-26	0.071
rs8176749	1st	A	0.161	4.38	0.575	5.30e-14	0.065	2.53	0.260	1.00e-21	0.072
(136131188)	2nd	A	0.188	5.14	0.576	1.11e-18	0.074	2.19	0.208	2.69e-25	0.066
rs8176746	1st	A	0.167	4.35	0.568	3.89e-14	0.067	2.55	0.255	1.34e-22	0.075
(136131322)	2nd	A	0.198	5.44	0.562	1.33e-21	0.083	2.16	0.204	1.79e-25	0.068
rs2073824	1st	G	0.496	1.35	0.430	>1e-7	-	1.15	0.193	4.00e-9	0.031
(136132633)	2nd	A	0.475	−1.85	0.462	>1e-7	-	−1.06	0.167	2.96e-10	0.030
rs657152	1st	A	0.440	2.66	0.424	5.13e-10	0.056	−0.554	0.196	>1e-7	-
(136139265)	2nd	A	0.454	3.38	0.456	2.01e-13	0.060	−0.697	0.168	>1e-7	-
rs500498	1st	A	0.455	−2.47	0.421	6.26e-9	0.054	0.385	0.195	>1e-7	-
(136148647)	2nd	A	0.445	−3.00	0.457	6.58e-11	0.050	0.424	0.168	>1e-7	-
rs505922	1st	G	0.450	2.54	0.419	1.95e-9	0.056	−0.465	0.194	>1e-7	-
(136149229)	2nd	G	0.458	3.57	0.453	5.83e-15	0.068	−0.645	0.167	>1e-7	-
rs495828	1st	A	0.281	0.27	0.481	>1e-7	-	−2.27	0.210	4.27e-26	0.087
(136154867)	2nd	A	0.264	−0.0726	0.509	>1e-7	-	−2.36	0.176	8.34e-39	0.102
rs7025162	1st	A	0.415	−0.815	0.428	>1e-7	-	−1.40	0.192	5.37e-13	0.044
(136166346)	2nd	A	0.398	−0.695	0.467	>1e-7	-	−1.26	0.167	7.55e-14	0.039

*1st population from the Takahata cohort, 2nd from the Yamagata cohort.

**Regression coefficient.

***Degrees of freedom adjusted R squared.

**Table 2:** Associations of ACE1 gene in 17q23.2 with ACE level in Takahata and Yamagata populations.

SNPs (position)		minor		ACE			
	stage*	allele	MAF	beta**	SE	p value	ARS***
rs4459609	1st	C	0.371	3.10	0.1868	5.76e-56	0.184
(61548948)	2nd	C	0.356	2.47	0.1658	3.32e-47	0.124
rs4309	1st	G	0.426	3.32	0.1772	2.97e-69	0.222
(61559923)	2nd	G	0.409	3.18	0.1531	1.55e-85	0.210
rs4311	1st	A	0.349	3.26	0.1849	2.59e-62	0.203
(61560763)	2nd	A	0.346	2.58	0.1684	1.44e-49	0.131
rs4329	1st	A	0.358	3.28	0.1841	2.12e-63	0.206
(61563458)	2nd	A	0.342	2.65	0.1643	1.68e-54	0.143
rs4343	1st	G	0.360	3.26	0.184	9.92e-63	0.204
(61566031)	2nd	G	0.342	2.65	0.1643	1.14e-54	0.143
rs4353	1st	A	0.440	3.97	0.1675	1e-102	0.311
(61570422)	2nd	A	0.422	3.47	0.1481	7.09e-105	0.251
rs4362	1st	A	0.442	3.99	0.1671	3.44e-104	0.314
(61573761)	2nd	A	0.426	3.49	0.1475	5.21e-107	0.254
rs4461142	1st	A	0.434	1.99	0.1884	4.98e-25	0.085
(61578048)	2nd	A	0.433	1.86	0.1611	1.13e-29	0.079

*1st population from the Takahata cohort, 2nd from the Yamagata cohort.

**Regression coefficient.

***Degrees of freedom adjusted R squared.

Previously reported associations of ABO with red blood cell related traits [4] or liver enzymes [7] in Asian populations were not found in our populations possibly due to specific factors to each study (c.f. sample size, local/global differences of populations, and/or measurement error inherent in such values). Moreover, although, in this 1st screening using the SNP array in our population, some SNPs were suspected around the genome-wide threshold for other phenotypes, we first focus on this only simultaneous effect of P-LIP and ACE to perform then a replication study in independent sample set.

### Confirmatory genotyping in the 2nd stage

By genotyping of these associated SNPs using a custom BeadChip (Illumina) in a different sample population of 1,671 individuals from the Yamagata cohort, we successfully confirmed all these associations at highly significant levels ranging from p = 6.58×10^−11^ (rs500498) to 1.33×10^−21^ (rs8176746) for P-LIP and from 2.96×10^−10^ (rs2073824) to 8.34×10^−39^ (rs495828) for ACE in 9q32, and also from 1.13×10^−29^ (rs4461142) to 5.21×10^−107^ (rs4362) for ACE in 17q23.2, as listed in Tables 1 and 2. Of these SNPs, rs2073824 had a discordant minor allele between the Takahata and Yamagata populations probably due to poor clustering in the 2nd assay (Figures S1 and S2 in File S1), so we eliminated it for further analyses.

### Adjustments for potential confounders

These associations were robust to additional adjustment for alcohol intake (for P-LIP) or smoking (for ACE) as potential confounders, and to the Box-Cox transformations for normalizing these endophenotypic distributions having a long-tail (Tables S3 and S4 in File S1). In addition, because the most common and largest potential confounder is treatment of hypertension such as administration of some ACE inhibitors, we tried to control it by adjustment for the dichotomized treatment history for hypertension (i.e. treated or not) along with age and gender, and then found that these associations were robust to this adjustment and even to a subgroup analysis in which certainly untreated individuals in both populations (840 in Takahata and 844 in Yamagata) were combined in a single group to allow direct comparisons with foregoing analyses because of its compatible sample size (1,684) with the original 1st or 2nd populations (Tables S5 and S6 in File S1).

### Association pattern and linkage disequilibrium structures

**Figure 2:**
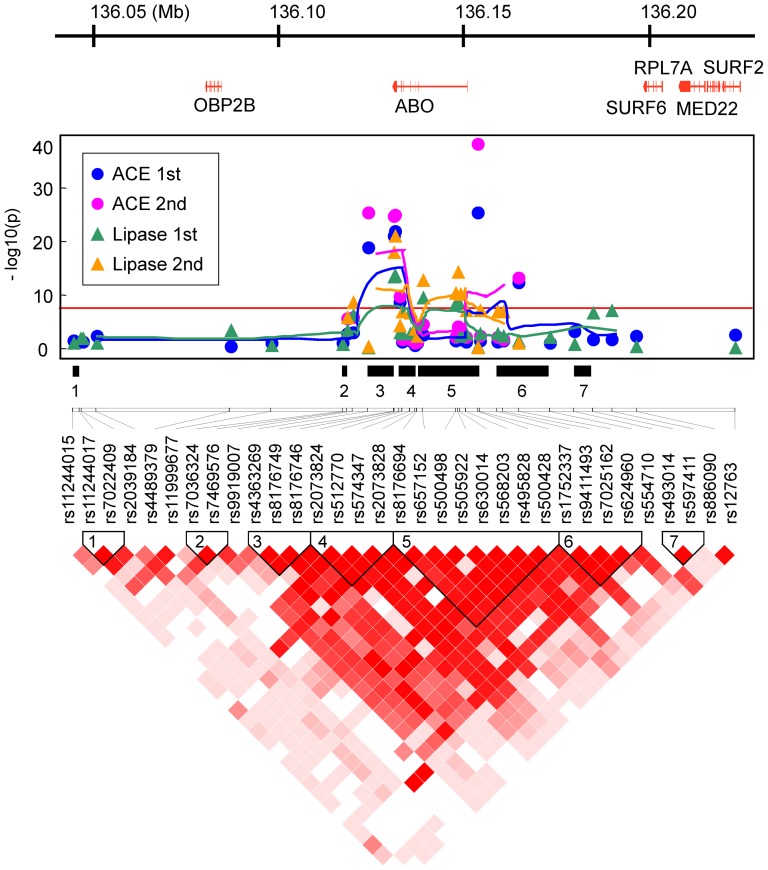
Gene map from RefSeq (upper panel), association pattern (middle), and LD structure (lower) by D′ measure in the Takahata population, in the 186 kb region from 136,040,829 to 136,227,260 of 9q32, using the coordinate system of the human genomic DNA sequence (GRCh37/hg19). The association pattern is expressed by plots of −log(p-value) with lines connecting five-point moving averages for SNPs around the ABO and ACE1 loci in the Takahata (1st) and Yamagata (2nd) populations. The numbered black bars indicate LD blocks defined by a confidence-interval of D′ measure that reflects those in the LD structure panel.

**Figure 3:**
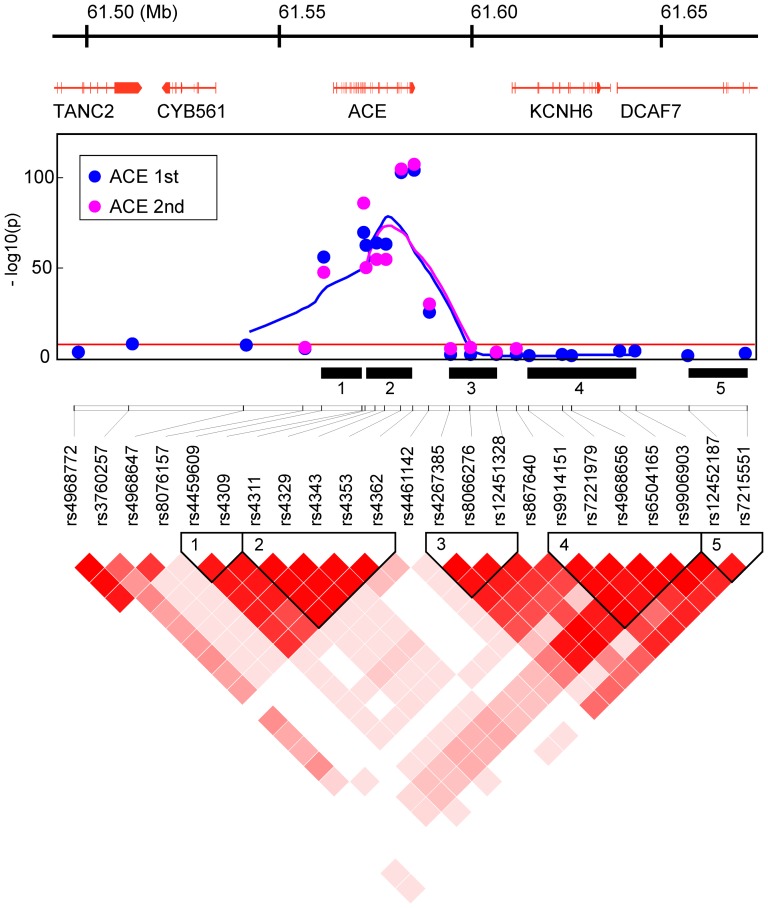
Gene map from RefSeq (upper panel), association pattern (middle), and LD structure (lower) by D′ measure in the Takahata population, in the 183 kb region from 61,481,619 to 61,664,990 of 17q23.2, using the coordinate system of the human genomic DNA sequence (GRCh37/hg19). The association pattern is expressed by plots of −log(p-value) with lines connecting five-point moving averages for SNPs around the ABO and ACE1 loci in the Takahata (1st) and Yamagata (2nd) populations. The numbered black bars indicate LD blocks defined by a confidence-interval of D′ measure that reflects those in the LD structure panel.

The detailed association patterns and linkage disequilibrium (LD) structures within about 180 kb of genomic segments around the ABO and ACE1 loci suggested that these genes themselves, rather than adjacent genes, affect each endophenotype in the two independent populations (Figures 2 and 3). Moreover, these patterns are very similar between the 1st and 2nd populations, implying that there is no serious bias from potential population structure.

### ABO association with the P-LIP level

As the results of this two-stage study, we newly identified the robust and strong associations of eight common variants at the ABO locus with the plasma level (i.e. leaking level) of P-LIP potentially correlated to some pathological status of the pancreas. For example, the most significant SNP, rs8176746 that causes a nonsynonymous amino-acid change (Leu266Met) chiefly dividing between the B and other lineages (Table S7 in File S1), can account for about 6.7 percent of the total variance in the P-LIP level in the Takahata population (8.3% of that in the Yamagata), in terms of degrees of freedom adjusted R squared (ARS; also called coefficient of determination) listed in Table 1. Intriguingly, a robust association of the ABO histo-blood group with pancreatic cancer reported in earlier epidemiological studies has been established using a modern genome-wide association study [8]. Our finding that T (complementary A) allele of rs505922 is protective against the elevation of the P-LIP level is compatible with their results that the T allele in complete LD with the O allele may express a low risk of pancreatic cancer.

### ABO association with the ACE level

In our single two-stage study, we successfully confirmed the known ACE1 and ABO associations with the ACE level [9] after adjustments for possible confounders. No SNP pair from these two loci in 9q32 and 17q23.3 shows strong linkage disequilibrium (D′ = 0.0019–0.15; r^2^ = 8.47×10^−7^–0.00576 in Takahata; 0.0061–0.16 and 3.50×10^−5^–5.79×10^−3^ in Yamagata) and has any joint effect (i.e. interaction) on the ACE level (p = 0.011–0.996 in Takahata; 0.0017–0.934 in Yamagata), indicating that they are independent of each other; the most significant SNP, rs4362, at the ACE1 locus can account for a large percentage of the total variance of the ACE level (31.4% in Takahata and 25.4% in Yamagata) and also rs495828 in ABO can independently account for a considerable proportion (8.7% in Takahata and 10.2% in Yamagata).

### Pleiotrophism at the ABO locus

**Figure 4:**
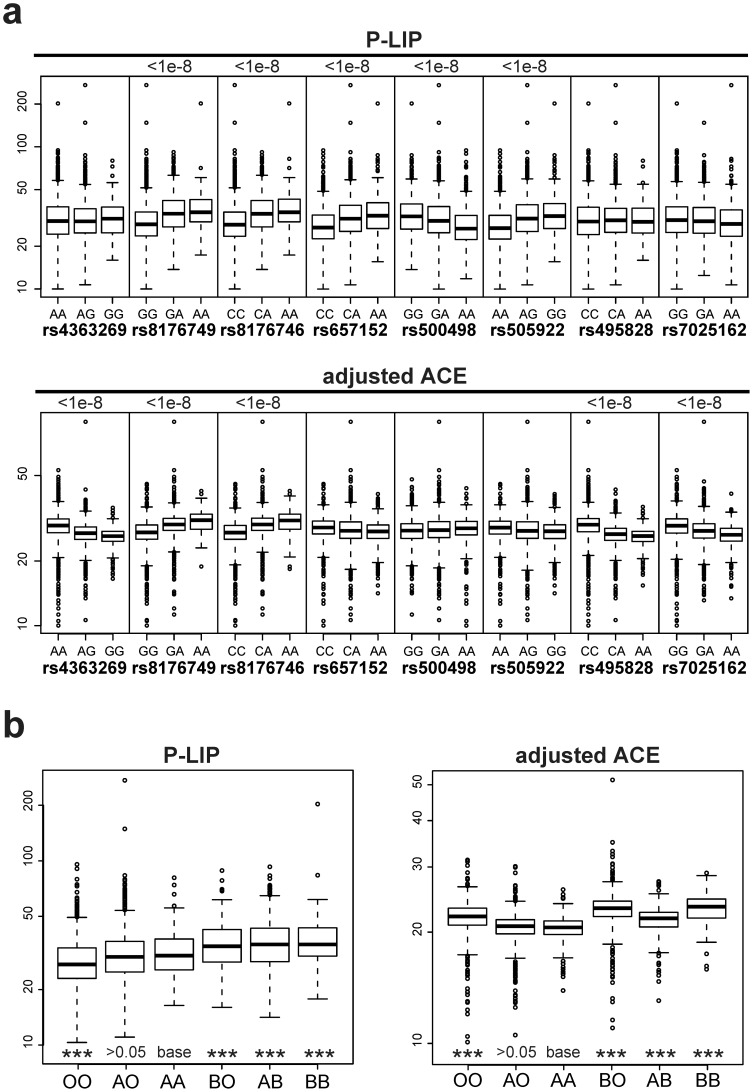
ABO locus and PLIP/ACE levels. (a) Box plots for the P-LIP and ACE level per each genotype of the significant SNPs at the ABO locus in the combined population of Takahata and Yamagata with adjustment for age and gender as covariates. These plots show the difference and direction of effects of each SNP on P-LIP and ACE. (b) One-way ANCOVA for the means of the P-LIP and ACE levels per genomically-deduced ABO group in the combined population with adjustment for age and gender as well as rs4356 (the effect of ACE1), in which AA is set as the baseline group in the corner-point parameterization denoted as “base”. Asterisks denote that the significance level reached in the one-way ANCOVA (*** <0.001).

**Figure 5:**
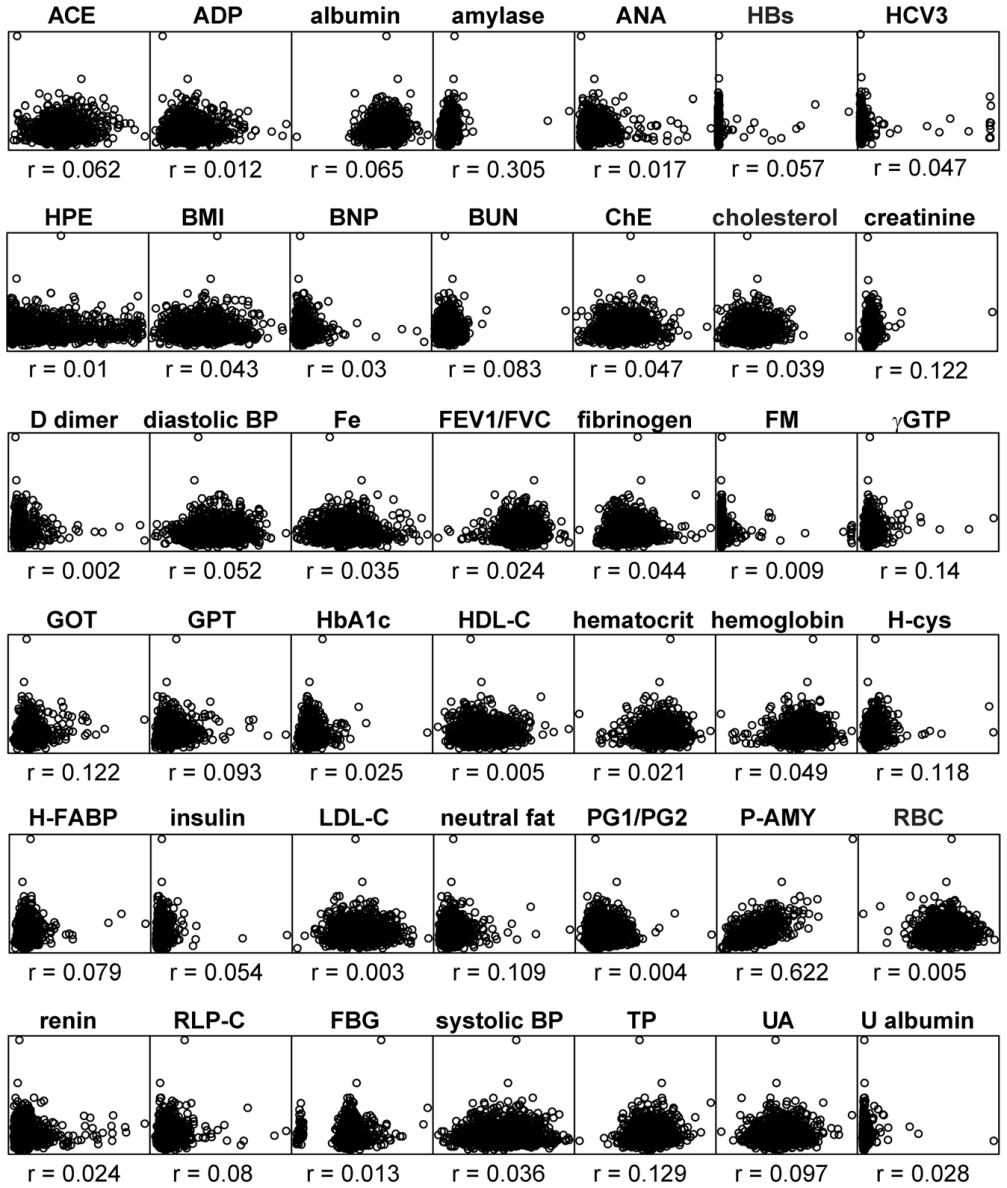
Scatter plots of the P-LIP levels with each of the other clinical test values in the Takahata population, where the plotted values are residuals from linear regression of each test value on age and gender. These plots show no strong correlation of each of other phenotypes to P-LIP. Each r denotes Pearson's (product-moment) correlation coefficient. The names and abbreviations of the total of 43 quantitative values are listed below (asterisks denote that it is plasma level); ACE (angiotensin converting enzyme*), ADP (adiponectin*), albumin*, amylase*, ANA (antinuclear antibody*), HBs (hepatitis Bs antigen*), HCV3 (hepatitis C virus 3 antibody, index*), HPE (Helicobacter pylori antibody E*), BMI (body mass index), BNP (B-type natriuretic peptide*), BUN (blood urea nitrogen*), ChE (cholinesterase*), cholesterol (total cholesterol*), creatinine*, D dimer*, diastolic BP (diastolic blood pressure), Fe (iron*), FEV1/FVC (forced expiratory volume in 1s/forced vital capacity), fibrinogen*, FM (fibrin monomer complex*), gGTP (gamma glutamyl transpeptidase*), GOT (asparate aminotransferase*), GPT (alanine aminotransferase*), HbA1c (hemoglobin A1c*), HDL-C (high density lipoprotein cholesterol*), hematocrit, hemoglobin, H-cys (homocysteine*), H-FABP (Heart type fatty acid-binding protein*), insulin (before a meal*), LDL-C (low density lipoprotein cholesterol*), P-LIP (pancreatic lipase*), neutral fat*, PG1/PG2 (pepsinogen 1/2 ratio), P-AMY (pancreatic amylase*), RBC (red blood cell counts), renin*, RLP-C (remnant-like particle cholesterol*), FBG (fasting blood glucose*), systolic BP (systolic blood pressure), TP (total protein*), UA (uric acid*), and U albumin (urinary albumin).

**Figure 6:**
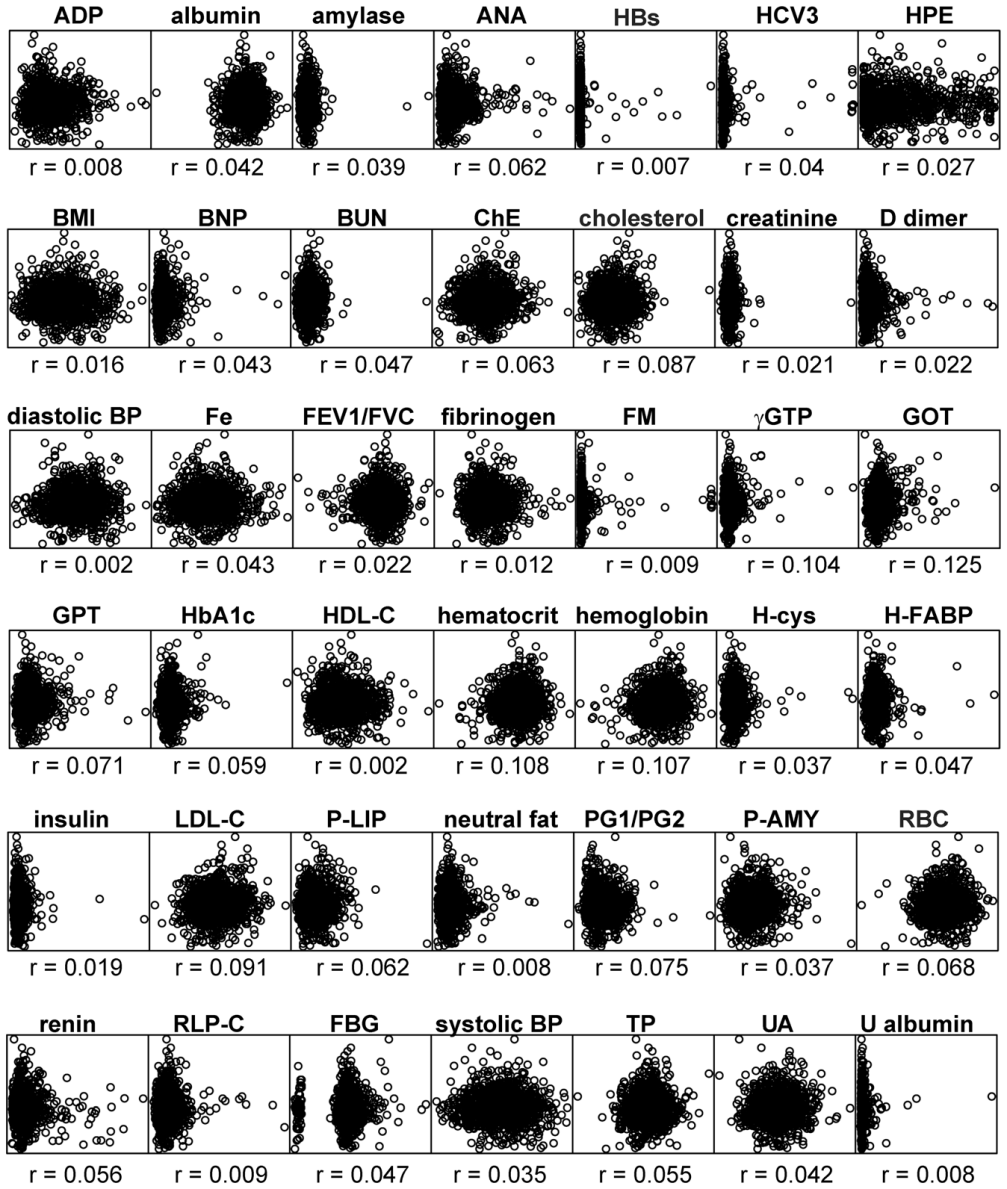
Scatter plots of the ACE levels with each of the other clinical test values in the Takahata population. These plots show no strong correlation of each of other phenotypes to ACE. Each r denotes Pearson's (product-moment) correlation coefficient. The names and abbreviations of the total of 43 quantitative values are same as Figure 5.

Box-plots of the plasma levels of P-LIP and ACE per each genotype of two significant SNPs at the ABO locus, rs8176749 and rs8176746, shared by both the endophenotypes, show that their effects are apparently parallel with respect to two traits, which tend to elevate the levels of both the enzymes with increase of minor allele in each of these SNPs (Figure 4a). One might, therefore, regard them as mere secondary effects resulting from a possible single pathologic change (i.e. confounding) such as renal insufficiency or hypertension. However, the P-LIP and ACE levels share no strong correlation to any of other endophenotypes including the creatinine level, urinary albumin and blood pressure in the Takahata population (Figures 5 and 6), and none of these endophenotypes show any strong association signal on 9q32 and 17q23.2 (Figure S3 in File S1), together suggesting that potential confounding is vanishingly small, if any, among them. Moreover, it is important to note that three of the remaining SNPs at the ABO locus, rs657152, rs500498 and rs505922, appear to have an opposite effect on the P-LIP and ACE levels, although the effect attained a genome-wide significance level only for P-LIP even in the combined population (Tables S8 and S9 in File S1); if one is elevated with an increase of a minor allele of any of these SNPs, the other would be reduced (Figure 4a). The lack of the association of these SNPs with the ACE levels, which are located in an anterior part of this gene near a variant determining the O group, may result in a largely discordant association pattern of the genomically-deduced ABO histo-blood groups with each enzyme level (Figure 4b); for example, individuals with the OO group, which has been well known as being protective against pancreatic cancer [8], is similarly protective (p<0.001 in one-way ANCOVA) against the elevation of the P-LIP level compared to AA, but, in contrast, susceptible (p<0.001) to that of the ACE level. Together with the fact that there is no obvious correlation (Pearson's correlation coefficient r = 0.077) between the P-LIP and ACE levels even after eliminating the effect of the ACE1 locus, we conclude that the parallel effect of these common variants at the ABO locus on these endophenotypes is pleiotropic, rather than two different outcomes from a primary pathologic change, according to a traditional definition as “a number of distinct and seemingly unrelated phenotypic effects of a single gene [10].”

In addition, we applied a phenotype defined as the ratio of ACE and P-LIP values, namely ACE/P-LIP, to calculate the P-gain statistics [11], in order to assess the direction of the effect of these SNPs on the two traits. As listed in Table 3, of 17 SNPs originally associated with P-LIP or ACE, 14 SNPs passed the P-gain threshold (>43; the number of all tested traits) for both 1st and 2nd stage samples, suggesting that they have opposing effect to P-LIP and ACE respectively. In contrast, three remaining SNPs, rs8176749, rs8176746 and rs2073824, failed to pass this threshold because they have the same elevation direction between P-LIP and ACE. These results suggest that the pleiotropic effect of the ABO locus on P-LIP and ACE is partly opposing.

**Table 3:** Antagonistic pleiotropism of ABO gene in 9q32 with ACE/P-LIP in Takahata and Yamagata populations.

SNPs (position)		minor		ACE/P-LIP			
	stage*	allele	MAF	beta**	SE	p value	P-gain
rs4363269	1st	G	0.287	−0.0869	0.012352	3.33e−12	1.26e+11
(136123840)	2nd	G	0.269	−0.0984	0.012118	9.26e−16	9.62e+14
rs8176749	1st	A	0.161	0.022	0.016694	>0.05	1.03e−15
(136131188)	2nd	A	0.188	0.002	0.014242	>0.05	1.14e−24
rs8176746	1st	A	0.167	0.023	0.016458	>0.05	2.61e−16
(136131322)	2nd	A	0.198	−0.0054	0.013962	>0.05	1.24e−24
rs2073824	1st	G	0.496	0.024	0.012196	>0.05	2.20e−2
(136132633)	2nd	A	0.475	−0.0192	0.011215	>0.05	1.79e−4
rs657152	1st	A	0.440	−0.0814	0.011028	2.84e−13	1.75e+10
(136139265)	2nd	A	0.454	−0.1013	0.009994	1.80e−23	1.47e+18
rs500498	1st	A	0.455	0.073	0.010976	4.00e−11	1.16e+9
(136148647)	2nd	A	0.445	0.089	0.010965	1.26e−15	7.07e+12
rs505922	1st	G	0.450	−0.0767	0.010916	3.42e−12	4.74e+9
(136149229)	2nd	G	0.458	−0.1038	0.009933	8.67e−25	9.48e+19
rs495828	1st	A	0.281	−0.1077	0.012212	3.80e−18	4.24e+16
(136154867)	2nd	A	0.264	−0.1146	0.01097	8.50e−25	5.99e+23
rs7025162	1st	A	0.415	−0.0469	0.012084	1.1e−4	5.55e+2
(136166346)	2nd	A	0.398	−0.0462	0.011234	4.11e−5	8.19e+2

*1st population from the Takahata cohort, 2nd from the Yamagata cohort.

**Regression coefficient.

### Statistically independent SNPs on the ACE1 and ABO locus

We applied the PLINK –clump option to the 1st stage GWAS data with the parameters used in Ahn et al. (2012) [12]. For ACE, five clumped SNP groups, G1 represented by rs4362 (for rs4459609, rs4309, rs4311, rs4329, rs4343, rs4353, and rs4461142), G2 by rs2447447 (for rs7210972, rs4968755, rs1518774, rs2460111, and rs2447442), G3 by rs1588368, G4 by rs495828 (for rs4363269 and rs7025162), and G5 by rs8176746 (for rs8176749 and rs2073824), are identified, while, for P-LIP, G6 by rs8176746 (for rs8176749, rs657152, rs500498, and rs505922) is identified as an only clumped SNP group (Table 4).

**Table 4:** Associations of clumped SNP groups with ACE and P-LIP in Takahata population.

traits	Clumped group	representative SNP (locus)	transformation	p value	(Clumped SNPs)
ACE	G1	rs4362	none	3.44e-105	(rs4459609, rs4309, rs4311, rs4329, rs4343, rs4353, rs4461142)
		(ACE1)	Box-Cox	1.04e-103	
	G2	rs2447447	none	7.59e-3	(rs7210972, rs4968755, rs1518774, rs2460111, rs2447442)
		(ACE1)	Box-Cox	6.73e-3	
	G3	rs1588368	none	>0.05	None
		(ACE1)	Box-Cox	>0.05	
	G4	rs495828	none	1.55e-27	(rs4363269, rs7025162)
		(ABO)	Box-Cox	1.23e-26	
	G5	rs8176746	none	2.06e-14	(rs8176749, rs2073824)
		(ABO)	Box-Cox	9.58e-14	
PLIP	G6	rs8176746	none	3.92e-14	(rs8176749, rs657152, rs500498, rs505922)
		(ABO)	Box-Cox	4.46e-17	

Following to the Ahn et al.'s (2012) approach [12], we performed multivariate linear regression model by using the clumped SNP group as predictors, where age and sex are set as covariates. As listed in Table 4, here appears no large difference of the results between untransformed and optimally Box-Cox transformed traits. For effects of the ACE1 on ACE, stringent significance appeared at G1 (P = 3.44×10^−105^ for untransformed; P = 1.04×10^−103^ for Box-Cox transformed), while G2 showed moderate significance (P = 7.59×10^−3^; P = 6.73×10^−3^) and G3 was not significant (P>0.05; P>0.05). For effects of the ABO on ACE, stringent significance appeared at G4 (P = 1.55×10^−27^; P = 1.23×10^−26^), G5 (P = 2.06×10^−14^; P = 9.58×10^−14^). For the ABO locus on P-LIP, G6 shows strong significance (P = 3.92×10^−14^; P = 4.46×10^−17^). The above results possibly means that these four clumped SNP groups (G1 and G2 on the ACE1, G4 and G5 on the ABO) have independent effect on ACE, while G6 on the ABO has independent effect on P-LIP. Of these groups, G5 and G6 share rs8176746 (associated with B blood as an alternative to O/A) but rs505922 (with O). On the other hand, G4 share no same SNP with G5 or G6, so it is independent. G6 may alternatively harbor converse effects by rs8176746 associated with B blood or by O with rs505922 on P-LIP. These results are largely consistent with results from single SNP analyses and ANCOVA for ABO blood type (Figure 4b), although there appears to be some difficulties for interpretation due to potential increase of variability through the multiple application of various kinds of statistical estimations.

## Discussion

By the two-stage study using two independent sample populations, we identified the association of rs505922 at the ABO locus on the elevation of the leaking P-LIP. Because this finding is highly compatible with the previous report of the protective effect of T allele of rs505922 (i.e. O group) with pancreatic cancer, one might regard the elevated enzyme levels with increase of alternative C (complementary G) allele of rs505922 as a simple consequence from the tissue damage by preclinical pancreatic cancer. However, given the relatively low incidence of pancreatic cancer (e.g. approximately 10 per 100,000) [13], it is unlikely that as large as 5–8.3 percent of the total variance in the P-LIP level in the general population could be explained by preclinical pancreatic cancer. Rather, our findings may enable us to hypothesize that the ABO blood group affects some unknown common status in the pancreas (e.g. pancreatic steatosis [14],[15]), yielding the variation of the plasma level of the leaking enzyme, prior to irreversible pathological changes such as cancer development and progression promoted by additional risk factors in a small fraction of the susceptible population, although causal relationships between the distribution of the P-LIP level in the general population and the pathogenesis of pancreatic cancer (see ref. [16],[17]) are still obscure. Our findings would warrant further investigations of causal inference between the variation of the P-LIP level and cancer development due to the ABO gene, using prospective data in our cohorts, coupled with detailed echographic examinations (or other imaging technologies) and more mechanistic studies, in order to test our hypothesis of the unknown common status in the pancreas.

Many earlier serological studies and recent genomic studies have reported that the ABO histo-blood groups show weak but consistent associations with a large number of traits including red blood cell phenotypes [4], soluble Intercellular adhesion molecule 1 level [18], stomach cancer [19], diabetes [20], pancreatic cancer [21], and duodenal ulcer [22], together implying the possibility that this gene has a pleiotropic effect. Our findings are the first demonstration of the pleiotropic effect of common variants at the ABO locus on the plasma levels of P-LIP and ACE using a single genome-wide data at the genome-wide significance level, even though the clinical significance of the P-LIP and ACE levels is not so straightforward. Including the opposing (i.e. “antagonistic”) effects on the P-LIP and ACE levels, the pleiotropism at the ABO locus may be supported by an extensive report of balancing selection on this gene [23], because such opposing effects could reduce the efficiency of negative selection pressure probably in combination with particular environmental perturbations, as suggested for several immune diseases [24],[25]. Moreover, such a pleiotropism seems to be widespread across a variety of disease-related genes such as particular alleles of major histocompatibility complex (MHC) [23], apolipoprotein E (ApoE) [25] and nucleotide-binding oligomerization domain protein 2 (NOD2) [26], implying the limitations (e.g. a serious source of drug side-effects) on a conventional strategy for preventative and therapeutic interventions, based on knowledge from any single effect of genes related to common complex human diseases, as have been mentioned in the context of system biology for molecular pathway (i.e. cascade/network) [27],[28].

So far, no mechanistic studies have successfully deciphered the functional basis for the ABO associations. However, the modern methodologies including extensive applications of genome-wide association study to genomic cohorts would provide new insights into the biochemical/biological basis that could promote novel mechanistic experimentations, as in a lesson from a series of recent studies for the entangled MHC associations with a large number of traits far from adaptive immune system that has been revealing the hidden roles and pathways consisting of functional interactions among MHC and diverse molecules [23],[29],[30].

## Subjects and Methods

### Subjects

The Takahata cohort was established for a baseline survey in a small rural town, Takahata Town, of Yamagata Prefecture in 2006–2008, whose total population size has been approximately 25 thousands through this period, whereas the Yamagata cohort is now ongoing in the urban prefectural capital, Yamagata City, having approximately 250 thousands residents, becoming part of our large genomic cohort initiative. For the 1st stage, we used genomic DNAs with 43 clinical test values (listed in the legend of Figure 5) from 1,639 individuals who completed the questionnaire for environmental exposures and informed consent for our modern prospective genomic cohort study. For the 2nd stage, from the Yamagata cohort, we collected 1,672 individuals enrolled in multiple sites as different as possible in order to avoid contamination of cryptic relatedness. The age and gender compositions are similar between them; average age is 61.3+/−10.2 (male:female = 1∶1.24) for Takahata and 63.3+/−8.6 (1∶1.13) for Yamagata. The detailed procedure of the DNA extraction has been described elsewhere [31]. This study was carried out under the approval by the ethical committee at Yamagata University and all other institutions involved.

### Genotyping

Using genomic DNAs from the Takahata population, we carried out genotyping for 657,366 SNPs by Infinium Assay with Human660W-Quad BeadChip (Illumina) according to the standard procedure provided by Illumina. After eliminating probes for copy number variation (CNV) detection, we applied a standard SNP quality control (QC) filter to this genome-wide data; SNP locus missing rate (lmiss) >0.05, MAF <0.01 and HWE p-value<0.000001. We also adopted a standard sample QC filter; individual missing rate (imiss) >0.01 and high heterozygosity >0.35. In addition, in order to avoid any bias from cryptic relatedness and potential population stratification, we eliminated higher lmiss samples from pairs with pair-wise IBD (identical by descent) sharing probability 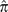
>(1/8+1/16)/2 and outliers until Tracy-Widom statistics p-value<0.05 for any principal component that they are calculated using a LD-based pruned autosomal SNP set (r∧2<0.01) through a stringent SNP-QC filters (lmiss >0.01; MAF <0.05; HWE p<0.05). Finally, we had 436,670 SNPs in 1,252 individuals (age = 61.1+/−9.97; gender ratio = 1∶1.28) for further analyses (Table S10 in File S1). For 20 SNPs in 9q32 and 13 in 17q23.2 around the significant SNPs that survived after imposing a genome-wide significance level or the Bonferroni's corrected level by the number of phenotypes to the 1st study, we newly carried out confirmatory genotyping in the Yamagata population by Golden Gate Assay with a custom BeadChip (Illumina) according to the standard procedure provided by Illumina as well. The quality of the genotypes in the second sample set was checked by the visual inspection of the clusters. Finally, we deduced the ABO histo-blood group for individuals in the two populations using rs505922, rs495828, rs8176749 and rs8176746, in addition to rs8176719 which is genotyped by PCR-RFLP according to a previous described procedure [32].

### Statistical analysis

Under the assumption of the additive effect of minor allele dosage, we carried out linear regression for each SNP from Infinium and Golden Gate Assays on the plasma levels of P-LIP and ACE separately with adjustment by age and gender as covariates, in which the statistical significance of each regression coefficient was evaluated by p value from the Wald test using the PLINK software package [33]. In addition, we carried out linear regression for the P-LIP and ACE levels on the same SNP set by applying an additional adjustment by potential confounders, alcohol intake (for P-LIP) and smoking (for ACE), and then by applying the Box-Cox normalizing transformation. We also carried out regression diagnosis for these analysis (Figure S4 in File S1). Finally, we carried out one-way ANCOVA (analysis of covariance) to compare mean levels of genomically-deduced ABO groups (i.e. six categories) in the combined population separately for the P-LIP and ACE, with adjustment for age and gender as well as rs4356 (i.e. the effect of ACE1), in which AA is set as the baseline group in the corner-point parameterization. These standard genetical/statistical analyses were done with the PLINK software package [33], the EIGENSTRAT software program [34] and the Haploview [35], in addition to the MASS package [36] and the R statistical environment [37].

## Supporting Information

File S1 **Supporting Information. Figure S1, Polar transformed cluster plot for the significant SNPs surviving the two-stage study for the Takahata and Yamagata. Figure S2, Polar transformed cluster plot for the significant SNPs surviving the two-stage study for the Takahata and Yamagata. Figure S3, Association patterns for SNPs in the 186 kb region from 136,040,829 to 136,227,260 of 9q32 with other clinical test values in the Takahata population, whose range is identical to that in** **Fig. 2a** **. The ABO locus is located within about 30 kb on the center of this range of 186 kb (** **Fig. 2a** **), which is enough to cover the ABO locus. Figure S4, The regression diagnosis plots for ACE and P-LIP after Box-Cox transformations or untransformations. Methods S1. Table S1, Associations of imputed genotypes in the ABO locus with P-LIP in Takahata population. Table S2, Associations of imputed genotypes in the ABO locus with ACE in Takahata population. Table S3, Associations of ABO gene in 9q32 with P-LIP or ACE levels after adjustment by smoking or Box-Cox transformation of trait values. Table S4, Associations of ACE1 in 17q23.3 with ACE level after adjustment by smoking or Box-Cox transformation of trait values. Table S5, Associations of ABO gene in 9q32 with P-LIP or ACE levels after adjustment or subgroup by treatment. Table S6, Associations of ACE1 gene in 17q23.2 with ACE level after adjustment or subgroup by treatment. Table S7, SNPs associated with P-LIP and/or ACE levels at a genome-wide significance level. Table S8, Associations of ABO gene in 9q32 with P-LIP and ACE in combined population. Table S9, Associations of ACE gene in 17q23.2 with ACE in combined population. Table S10, Sample numbers removed by each quality control filter. Text S1, Organization and all contributors list of Yamagata University Genomic Cohort Consortium.**
(PDF)

## References

[1] Wellcome Trust Case Control Consortium (2007) Genome-wide association study of 14,000 cases of seven common diseases and 3,000 shared controls. Nature 447: 661–678. 17554300 10.1038/nature05911 PMC2719288

[2] Manolio TA, Collins FS, Cox NJ, Goldstein DB, Hindorff LA, et al (2009) Finding the missing heritability of complex diseases. Nature 461: 747–753. 19812666 10.1038/nature08494 PMC2831613

[3] Wray NR, Visscher PM (2007) Mapping common disease genes. In: Genes and Common Disease. eds. Wright AF, Hastie N. (Cambridge: Cambridge University Press).

[4] Kamatani Y, Matsuda K, Okada Y, Kubo M, Hosono N, et al (2010) Genome-wide association study of hematological and biochemical traits in a Japanese population. Nat Genet 42: 210–215. 20139978 10.1038/ng.531

[5] Falconer DS, Mackay TFC (1996). Introduction to Quantitative Genetics, 4th edn. (Edinburgh: Longman).

[6] Yang J, Wray NR, Visscher PM (2010) Comparing apples and oranges: equating the power of case-control and quantitative trait association studies. Genet Epidemiol 34: 254–257. 19918758 10.1002/gepi.20456

[7] Yuan X, Waterworth D, Perry JR, Lim N, Song K, Chambers JC, et al (2008) Population-based genome-wide association studies reveal six loci influencing plasma levels of liver enzymes. Am J Hum Genet 83 (4) 520–528. 18940312 10.1016/j.ajhg.2008.09.012 PMC2561937

[8] Amundadottir L, Kraft P, Stolzenberg-Solomon RZ, Fuchs CS, Petersen GM, et al (2009) Genome-wide association study identifies variants in the ABO locus associated with susceptibility to pancreatic cancer. Nat Genet 41: 986–990. 19648918 10.1038/ng.429 PMC2839871

[9] Chung CM, Wang RY, Chen JW, Fann CS, Leu HB, et al (2010) A genome-wide association study identifies new loci for ACE activity: potential implications for response to ACE inhibitor. Pharmacogenomics J 10: 537–544. 20066004 10.1038/tpj.2009.70

[10] King RC, Stansfield WD (2006) A Dictionary of genetics, 7th edn. (Oxford: Oxford university press).

[11] Suhre K, Shin SY, Petersen AK, Mohney RP, Meredith D, et al (2011) Human metabolic individuality in biomedical and pharmaceutical research. Nature 477 (7362) 54–60. 21886157 10.1038/nature10354 PMC3832838

[12] Ahn R, Ding YC, Murray J, Fasano A, Green PH, et al (2012) Association analysis of the extended MHC region in celiac disease implicates multiple independent susceptibility loci. PLoS One 7 (5) e36926. 22615847 10.1371/journal.pone.0036926 PMC3355177

[13] Ferlay J, Bray F, Pisani P, Parkin DM (2004) GLOBOCAN 2002: Cancer Incidence, Mortality and Prevalence Worldwide. IARC CancerBase No. 5, (Lyon: IARC Press).

[14] Mathur A, Zyromski NJ, Pitt HA, Al-Azzawi H, Walker JJ, et al (2009) Pancreatic steatosis promotes dissemination and lethality of pancreatic cancer. J Am Coll Surg 208: 989–996. 19476877 10.1016/j.jamcollsurg.2008.12.026

[15] van Raalte DH, van der Zijl NJ, Diamant M (2010) Pancreatic steatosis in humans: cause or marker of lipotoxicity? Curr Opin Clin Nutr Metab Care 13: 478–485. 20489606 10.1097/MCO.0b013e32833aa1ef

[16] Malka D, Hammel P, Maire F, Rufat P, Madeira I, et al (2002) Risk of pancreatic adenocarcinoma in chronic pancreatitis. Gut 51 (6) 849–852. 12427788 10.1136/gut.51.6.849 PMC1773474

[17] Raimondi S, Lowenfels AB, Morselli-Labate AM, Maisonneuve P, Pezzilli R, et al (2010) Pancreatic cancer in chronic pancreatitis; aetiology, incidence, and early detection. Best Pract Res Clin Gastroenterol 24 (3) 349–358. 20510834 10.1016/j.bpg.2010.02.007

[18] Paré G, Ridker PM, Rose L, Barbalic M, Dupuis J, et al (2011) Genome-wide association analysis of soluble ICAM-1 concentration reveals novel associations at the NFKBIK, PNPLA3, RELA, and SH2B3 loci. PLoS Genet 7: e1001374. 21533024 10.1371/journal.pgen.1001374 PMC3080865

[19] Marcus DM (1969) The ABO and Lewis blood-group system. Immunochemistry, genetics and relation to human disease. N Engl J Med 280: 994–1006. 4888078 10.1056/NEJM196905012801806

[20] Vogel F, Motulsky AG (1982) Human Genetics: Problems and Approaches. Springer, Berlin.

[21] Tanikawa C, Urabe Y, Matsuo K, Kubo M, Takahashi A, et al (2012) A genome-wide association study identifies two susceptibility loci for duodenal ulcer in the Japanese population. Nat Genet 44: 430–434. 22387998 10.1038/ng.1109

[22] Cavalli-Sforza LL, Edwards A (1967) Phylogenetic analysis. Models and estimation procedures. Am J Hum Genet 19: 233–257. 6026583 PMC1706274

[23] Gregersen PK, Behrens TW (2006) Genetics of autoimmune diseases – disorders of immune homeostasis. Nat Rev Genet 7: 917–928. 17139323 10.1038/nrg1944

[24] Hindorff LA, Sethupathy P, Junkins HA, Ramos EM, Mehta JP, et al (2009) Potential etiologic and functional implications of genome-wide association loci for human diseases and traits. Proc Natl Acad Sci USA 106: 9362–9367. 19474294 10.1073/pnas.0903103106 PMC2687147

[25] Boerwinkle E, Utermann G (1988) Simultaneous effects of the apolipoprotein E polymorphism or apolipoprotein E, apolipoprotein B, and cholesterol metabolism. Am J Hum Genet 42: 104–112. 3337104 PMC1715322

[26] Rahman P, Bartlett S, Siannis F, Pellett FJ, Farewell VT, et al (2003) CARD15: a pleiotropic autoimmune gene that confers susceptibility to psoriatic arthritis. Am J Hum Genet 73: 677–681. 12879366 10.1086/378076 PMC1180694

[27] Dudley AM, Janse DM, Tanay A, Shamir R, Church GM (2005) A global view of pleiotropy and phenotypically derived gene function in yeast. Mol Syst Biol 1: 2005.0001. 10.1038/msb4100004 PMC1681449 16729036

[28] Boone C, Bussey H, Andrews BJ (2007) Exploring genetic interactions and networks with yeast. Nat Rev Genet 8: 437–449. 17510664 10.1038/nrg2085

[29] Jacob S, McClintock MK, Zelano B, Ober C (2002) Paternally inherited HLA alleles are associated with women's choice of male odor. Nat Genet 30: 175–179. 11799397 10.1038/ng830

[30] Boulanger LM, Shatz CJ (2004) Immune signalling in neural development, synaptic plasticity and disease. Nat Rev Neurosci 5: 521–531. 15208694 10.1038/nrn1428

[31] Konta T, Hao Z, Abiko H, Ishikawa M, Takahashi T, et al (2006) Prevalence and risk factor analysis of microalbuminuria in Japanese general population: The Takahata Study. Kidney Int 70: 751–756. 16807548 10.1038/sj.ki.5001504

[32] Shintani-Ishida K, Zhu BL, Maeda H, Uemura K, Yoshida K (2008) A new method for ABO genotyping to avoid discrepancy between genetic and serological determinations. Int J Legal Med 122: 7–9. 17242950 10.1007/s00414-006-0148-0

[33] Purcell S, Neale B, Todd-Brown K, Thomas L, Ferreira MA, et al (2007) PLINK: a tool set for whole-genome association and population-based linkage analyses. Am J Hum Genet 81: 559–575. 17701901 10.1086/519795 PMC1950838

[34] Patterson N, Price AL, Reich D (2006) Population Structure and Eigenanalysis. PLoS Genet 2: e190. 17194218 10.1371/journal.pgen.0020190 PMC1713260

[35] Barrett JC, Fry B, Maller J, Daly MJ (2005) Haploview: analysis and visualization of LD and haplotype maps. Bioinformatics 21: 263–265. 15297300 10.1093/bioinformatics/bth457

[36] Venables WN, Ripley BD (2002) Modern Applied Statistics with S. 4th edn. (New York: Springer).

[37] Ihaka R, Gentleman R (1996) R: a language for data analysis and graphics. J Comp Graph Stat 5: 299–314.

