# Supplementary material for: Pleiotropic Effect of Common Variants at ABO Glycosyltranferase Locus in 9q32 on Plasma Levels of Pancreatic Lipase and Angiotensin Converting Enzyme
Source: PLoS One. 2014 Feb 28;9(2):e55903. doi: 10.1371/journal.pone.0055903 (PMC3938410; doi:10.1371/journal.pone.0055903)
Supplement: File S1 — Supporting Information. Figure S1, Polar transformed cluster plot for the significant SNPs surviving the two-stage study for the Takahata and Yamagata. Figure S2, Polar transformed cluster plot for the significant SNPs surviving the two-stage study for the Takahata and Yamagata. Figure S3, Association patterns for SNPs in the 186 kb region from 136,040,829 to 136,227,260 of 9q32 with other clinical test values in the Takahata population, whose range is identical to that in Fig. 2a . The ABO locus is located within about 30 kb on the center of this range of 186 kb ( Fig. 2a ), which is enough to cover the ABO locus. Figure S4, The regression diagnosis plots for ACE and P-LIP after Box-Cox transformations or untransformations. Methods S1. Table S1, Associations of imputed genotypes in the ABO locus with P-LIP in Takahata population. Table S2, Associations of imputed genotypes in the ABO locus with ACE in Takahata population. Table S3, Associations of ABO gene in 9q32 with P-LIP or ACE levels after adjustment by smoking or Box-Cox transformation of trait values. Table S4, Associations of ACE1 in 17q23.3 with ACE level after adjustment by smoking or Box-Cox transformation of trait values. Table S5, Associations of ABO gene in 9q32 with P-LIP or ACE levels after adjustment or subgroup by treatment. Table S6, Associations of ACE1 gene in 17q23.2 with ACE level after adjustment or subgroup by treatment. Table S7, SNPs associated with P-LIP and/or ACE levels at a genome-wide significance level. Table S8, Associations of ABO gene in 9q32 with P-LIP and ACE in combined population. Table S9, Associations of ACE gene in 17q23.2 with ACE in combined population. Table S10, Sample numbers removed by each quality control filter. Text S1, Organization and all contributors list of Yamagata University Genomic Cohort Consortium. (PDF) [file pone.0055903.s001.pdf]

Figure S1

ABO in 9q32

Infinium assay

rs4363269 rs8176749 rs8176746 rs2073824 rs657152

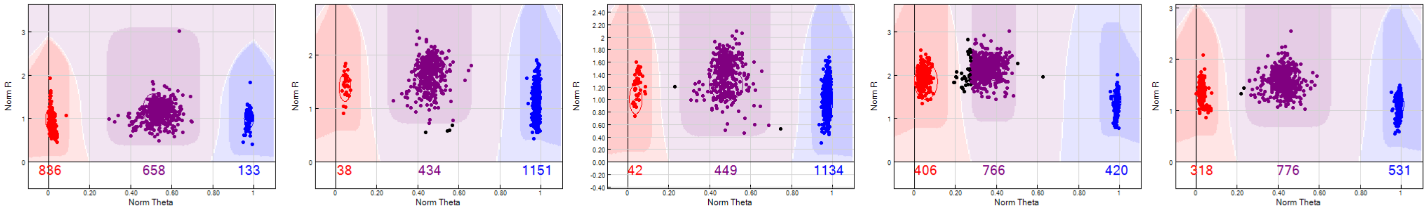

Infinium assay

rs500498 rs505922 rs495828 rs7025162

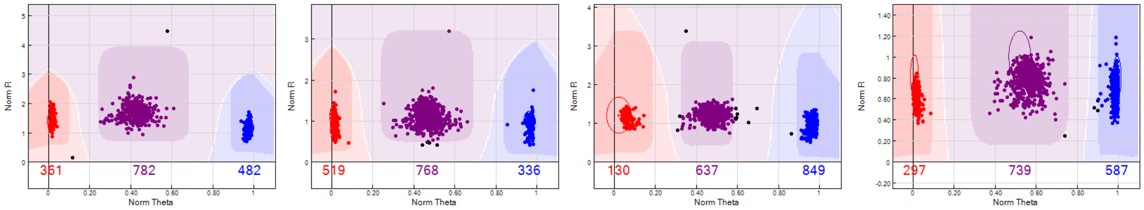

Golden Gate assay

rs4363269 rs8176749 rs8176746 rs2073824 rs657152

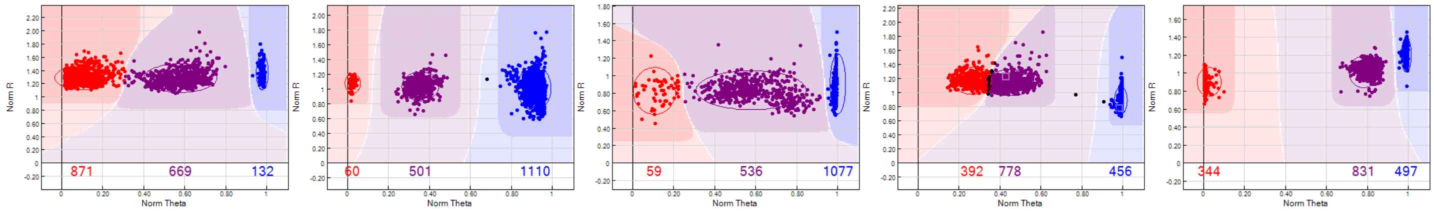

Golden Gate assay

rs500498 rs505922 rs495828 rs7025162

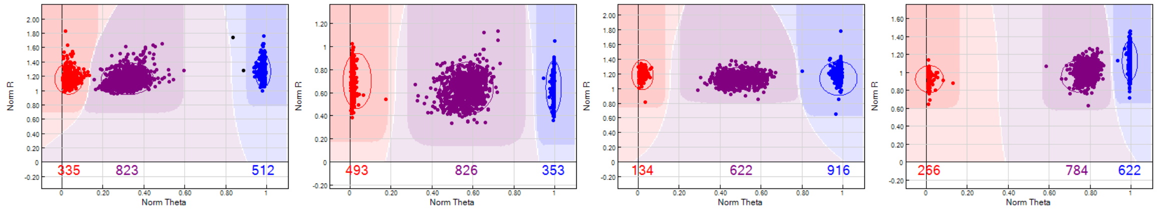

Figure S2

ACE1 in 17q23.2

Infinium assay

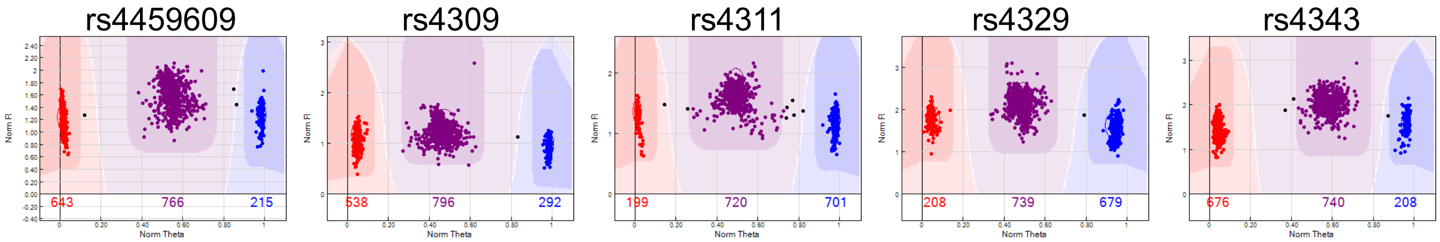

Infinium assay

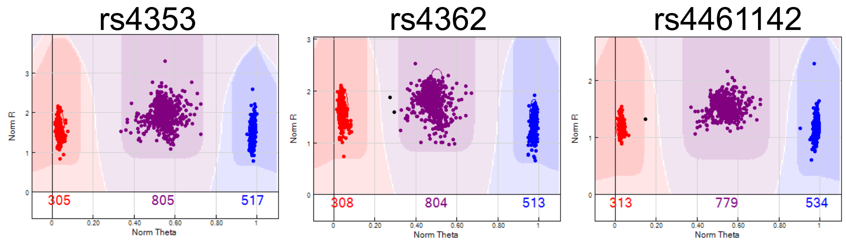

Golden Gate assay

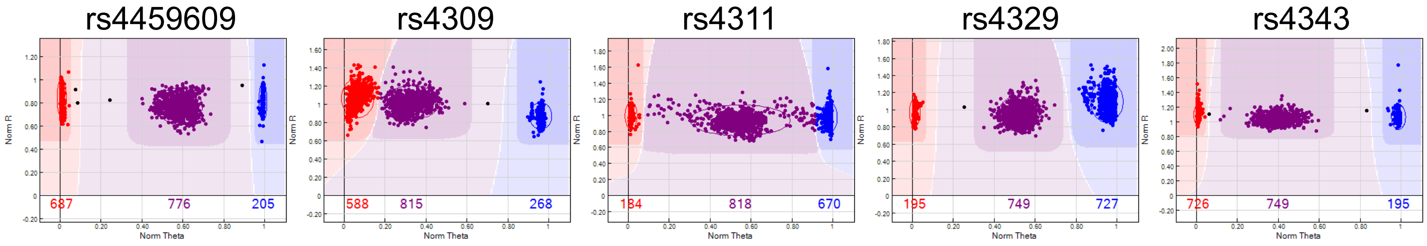

Golden Gate assay

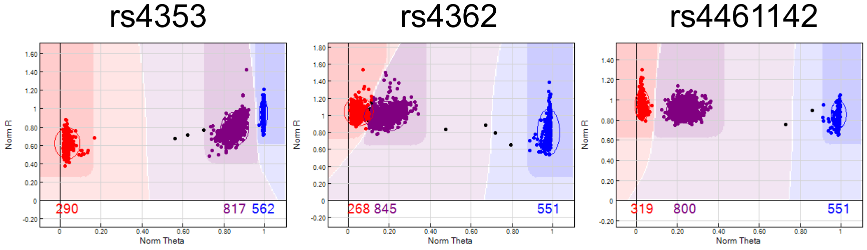

Figure S3

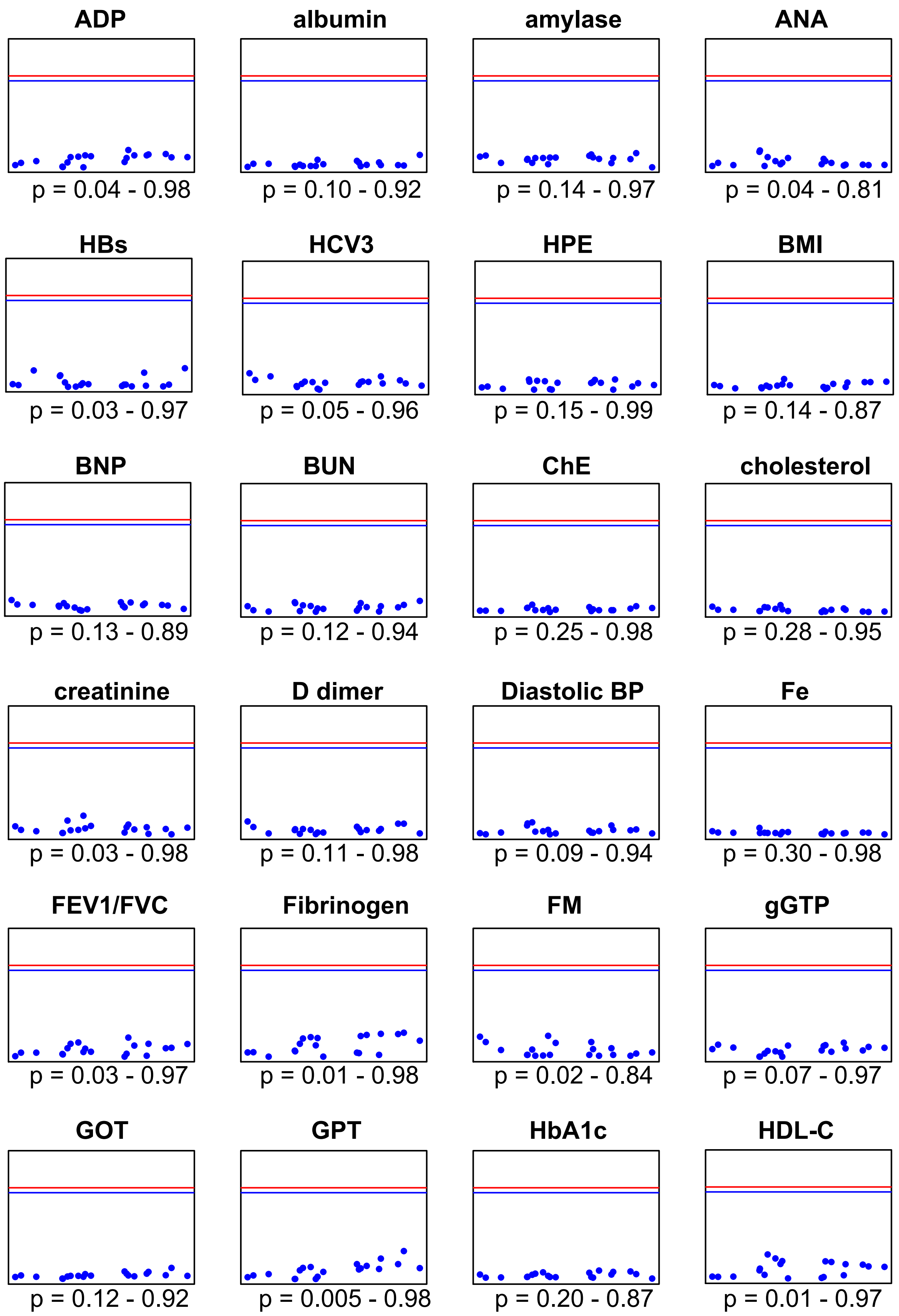
ADP
p = 0.04 - 0.98
albumin
p = 0.10 - 0.92
amylase
p = 0.14 - 0.97
ANA
p = 0.04 - 0.81
HBs
p = 0.03 - 0.97
HCV3
p = 0.05 - 0.96
HPE
p = 0.15 - 0.99
BMI
p = 0.14 - 0.87
BNP
p = 0.13 - 0.89
BUN
p = 0.12 - 0.94
ChE
p = 0.25 - 0.98
cholesterol
p = 0.28 - 0.95
creatinine
p = 0.03 - 0.98
D dimer
p = 0.11 - 0.98
Diastolic BP
p = 0.09 - 0.94
Fe
p = 0.30 - 0.98
FEV1/FVC
p = 0.03 - 0.97
Fibrinogen
p = 0.01 - 0.98
FM
p = 0.02 - 0.84
gGTP
p = 0.07 - 0.97
GOT
p = 0.12 - 0.92
GPT
p = 0.005 - 0.98
HbA1c
p = 0.20 - 0.87
HDL-C
p = 0.01 - 0.97

Figure S3 (continued)

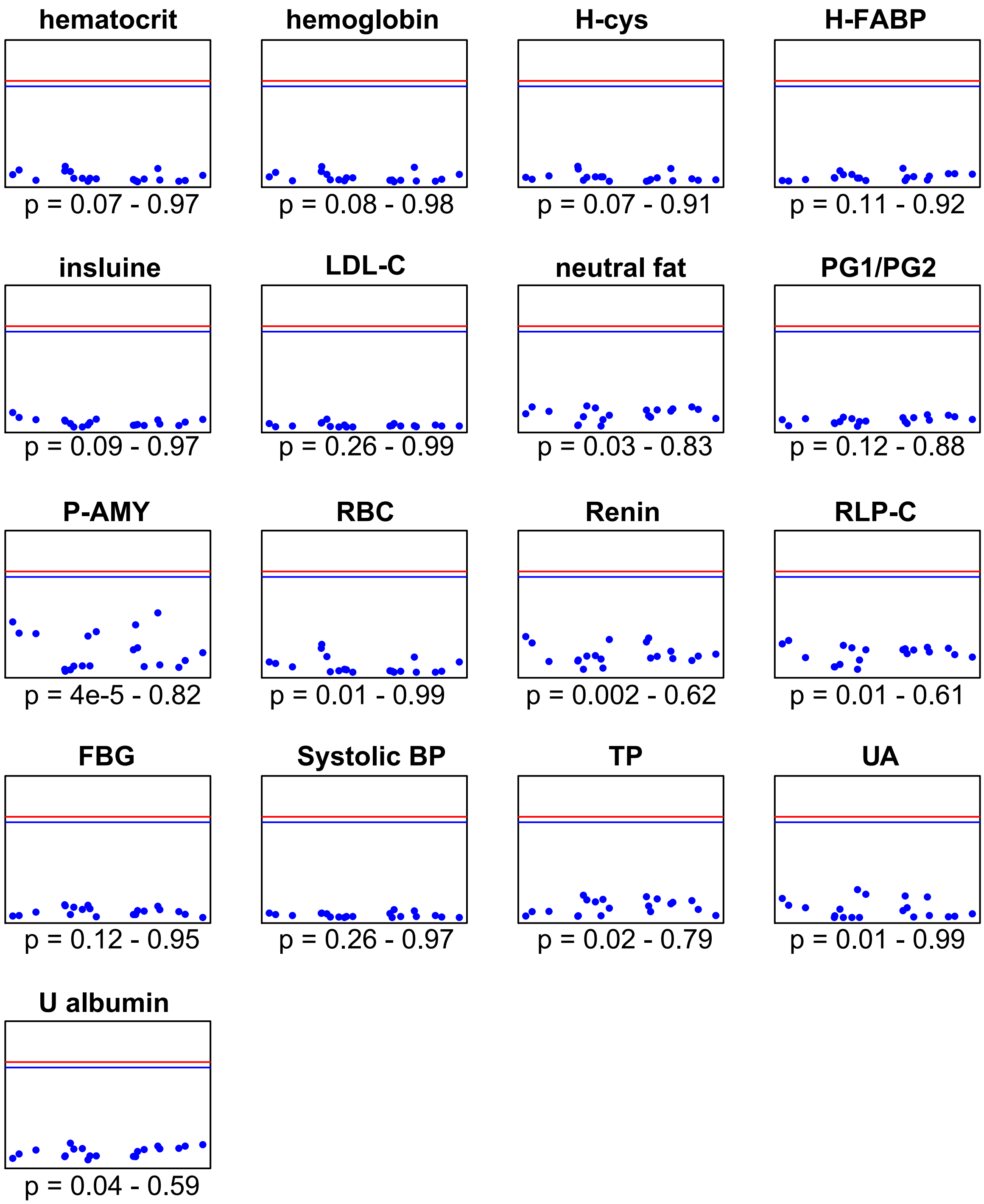

Figure S4

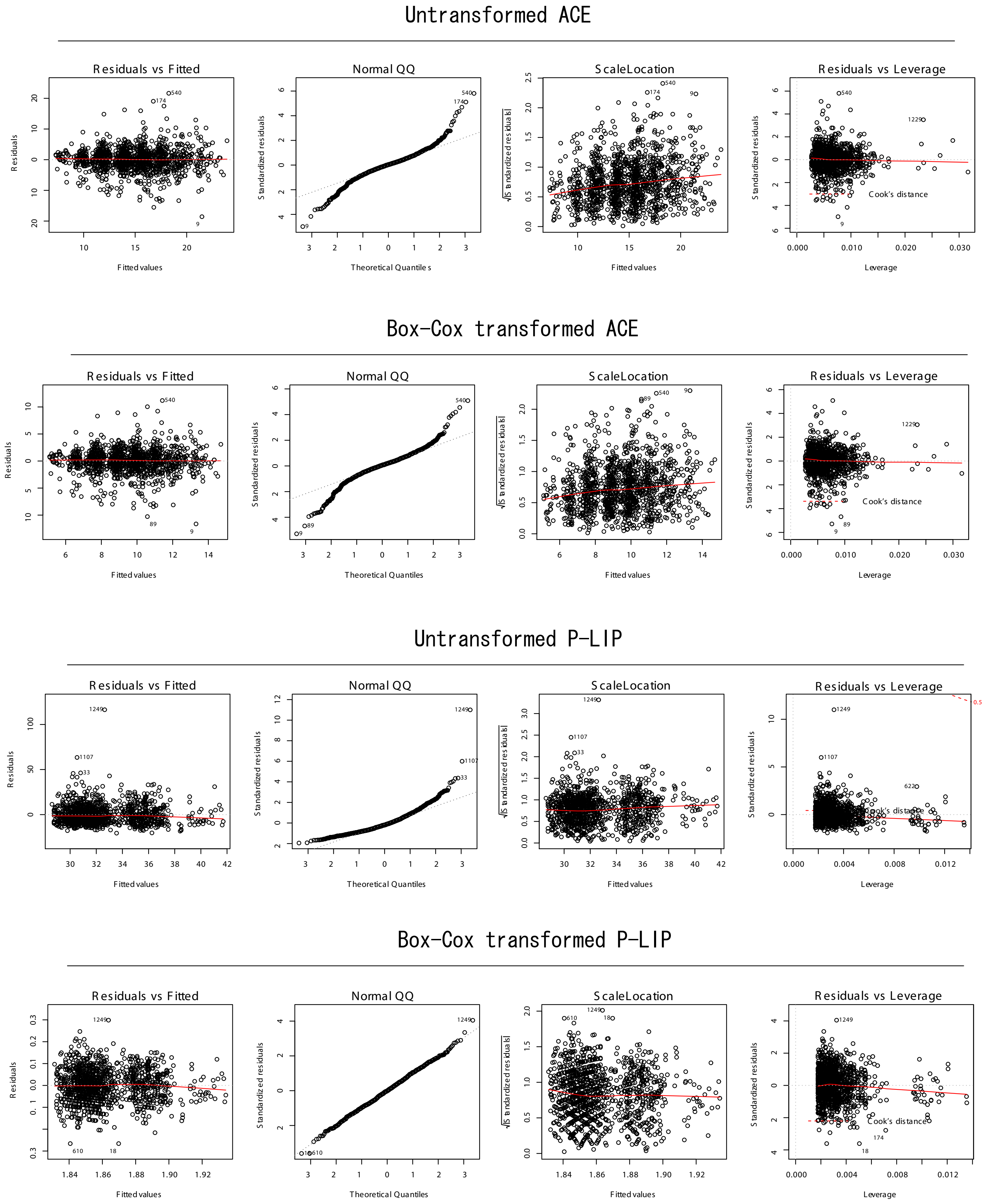
Untransformed ACE
Residuals vs Fitted
Normal QQ
ScaleLocation
Residuals vs Leverage
Fitted values
Theoretical Quantiles
Leverage
Residuals
Standardized residuals
Cook's distance
Box-Cox transformed ACE
Residuals vs Fitted
Normal QQ
ScaleLocation
Residuals vs Leverage
Untransformed P-LIP
Residuals vs Fitted
Normal QQ
ScaleLocation
Residuals vs Leverage
Box-Cox transformed P-LIP
Residuals vs Fitted
Normal QQ
ScaleLocation
Residuals vs Leverage

Methods S1

Imputation and test: We first carried out pre-phasing [1] using SHAPEIT [2] based on 1000 Genome Project for chromosome 9, then applied IMPUTE2 [3] to ABO locus to complete imputation for ABO locus. Subsequently we used SNPTEST [4] to examine quantitative traits association with loci after imputation, in which adjustment by age and sex was incorporated. The above procedures were conducted separately for the first and second stage datasets.

[1] Howie B, Fuchsberger C, Stephens M, Marchini J & Abecasis GR. Fast and accurate genotype imputation in genome-wide association studies through pre-phasing. Nat Genet 44(8): 955-959 (2012).

[2] Delaneau O, Marchini J & Zagury JF A linear complexity phasing method for thousands of genomes. Nat. Methods 9, 179-181 (2011).

[3] Howie BN, Donnelly P & Marchini J. A flexible and accurate genotype imputation method for the next generation of genome-wide association studies. PLoS Genet 5(6): e1000529 (2009).

[4] Marchini J, Howie B, Myers S, McVean G & Donnelly P. A new multipoint method for genome-wide association studies via imputation of genotypes. Nat

Genet 39: 906-913 (2007).

Regression diagnosis: We carried out regression diagnosis for each marker being significant in GWAS on both ACE and P-LIP for 1st and 2nd stage samples, utilizing four diagnosis plots implemented in R (fitted values vs residuals, normal QQ plot for standardized residuals, fitted values vs sqrt(standardized residuals), and leverage vs standardized residuals). We performed the diagnosis plots both for untransformed and for optimally Box-Cox transformed traits (lambda = 0.8 for ACE and lambda = -0.4 for P-LIP).

Table S1. Associations of imputed genotypes in the ABOlocus with P-LIP in Takahata population.

					Allele		average maximum		Genotypes							
index	id*	rsid	chr	pos	A	B	posterior call	info	AA	AB	BB	Null	MAF	beta	SE	P-value
1	---	rs73562368	NA	136123019	C	T	0.99972	0.0585888	1251.6	0.351	0	0	0.00014018	ND	ND	ND
2	---	rs11244049	NA	136123092	T	C	0.99546	0.98526	907.73	314.42	29.845	0.008	0.14941	0.34474	0.055764	6.3238e-10
3	---	rs148031614	NA	136123172	T	C	0.99928	0.25135	1251.1	0.9	0	0	0.00035942	ND	ND	ND
4	---	rs9328546	NA	136123267	C	G	0.93562	0.84338	838.89	376.56	36.519	0.034	0.17956	0.06006	0.058599	0.30539
5	---	rs114204311	NA	136123277	G	A	0.99987	0.083469	1251.8	0.166	0	0	0	ND	ND	ND
6	---	rs192446092	NA	136123600	A	C	0.9999	0.01904	1251.9	0.131	0	0	0	ND	ND	ND
7	---	rs183830736	NA	136123679	C	T	0.99982	0.10392	1251.8	0.221	0	0	0	ND	ND	ND
8	---	rs6597618	NA	136123740	T	C	0.99414	0.98988	459.62	583.73	208.63	0.01	0.39976	0.20029	0.040161	6.1246e-07
9	rs4363269	rs4363269	NA	136123840	A	G	1	1	634	517	101	0	0.28714	-0.0030706	0.044089	0.94448
10	---	rs186098467	NA	136123994	T	C	1	0.0029988	1252	0.003	0	0	0	ND	ND	ND
11	---	rs7025839	NA	136124190	G	A	0.99694	0.99335	703.13	474.76	74.108	0.005	0.24879	0.033506	0.046373	0.46998
12	---	rs72775410	NA	136124314	G	A	0.96768	0.3896	1204.1	47.501	0.434	0.002	0.019317	-0.02986	0.20035	0.88152
13	---	rs73562371	NA	136124326	T	C	0.99955	0.72665	1250.2	1.76	0	0	0.00070288	-0.96353	0.85558	0.26009
14	---	rs9411473	NA	136124399	G	A	0.99562	0.98996	702.96	474.93	74.095	0.021	0.24885	0.03319	0.046467	0.47506
15	---	rs9411370	NA	136124432	A	G	0.99538	0.98938	702.97	474.94	74.071	0.015	0.24884	0.033183	0.046484	0.47531
16	---	rs78755596	NA	136124590	T	A	0.99747	0.075064	1248.8	3.171	0	0	0.0012664	ND	ND	ND
17	---	rs115370344	NA	136124602	T	G	0.99982	0.017178	1251.8	0.225	0	0	0	ND	ND	ND
18	---	rs12338026	NA	136124857	C	G	0.9993	0.58542	1250.4	1.624	0	0	0.00064856	ND	ND	ND
19	---	rs7047076	NA	136125329	A	T	0.989	0.97695	702.56	475.81	73.599	0.031	0.24881	0.032534	0.046852	0.48743
20	---	rs193302481	NA	136125592	C	T	1	0.0009992	1252	0.002	0	0	0	ND	ND	ND
21	---	rs9411474	NA	136125716	C	G	0.98636	0.97321	702.37	476.16	73.432	0.031	0.24882	0.032289	0.046956	0.49168
22	---	rs61527302	NA	136125994	G	A	0.98778	0.97299	813.69	388.96	49.337	0.007	0.19474	0.077463	0.050608	0.12585
23	---	rs191151099	NA	136126026	T	A	1	1	1252	0	0	0	0	ND	ND	ND
24	---	rs10901251	NA	136126129	A	C	0.97626	0.96443	456.22	577.46	218.27	0.044	0.40497	0.20263	0.040336	5.0714e-07
25	---	rs148763789	NA	136126132	C	T	0.99999	0.0058286	1252	0.012	0	0	0	ND	ND	ND
26	---	rs188658949	NA	136126153	G	T	1	0.0019992	1252	0.002	0	0	0	ND	ND	ND
27	---	rs78320709	NA	136126394	C	T	0.989	0.49772	1231.6	20.321	0.068	0.002	0.0081697	-0.39605	0.26639	0.13709
28	---	rs34266669	NA	136126495	C	T	0.98406	0.96903	702.47	476.4	73.112	0.022	0.24866	0.032268	0.047075	0.49305
29	---	rs151316644	NA	136126625	G	A	0.99948	0.039007	1251.3	0.653	0	0	0.00026078	ND	ND	ND
30	---	rs3205136	NA	136126631	C	A	0.98673	0.96021	895.8	322.73	33.451	0.018	0.15561	0.33809	0.055421	1.0577e-09
31	---	rs7849280	NA	136126636	A	G	0.94202	0.87242	783.14	416.66	52.162	0.033	0.20807	0.028529	0.053719	0.59537
32	---	rs116958345	NA	136127122	A	C	0.99974	0.013815	1251.7	0.33	0	0	0.00013179	ND	ND	ND
33	---	rs9411475	NA	136127268	T	C	0.95823	0.92214	649.98	507.3	94.676	0.044	0.27823	0.018941	0.046111	0.68124
34	---	rs118065674	NA	136127346	C	T	0.99999	0.0019952	1252	0.012	0	0	0	ND	ND	ND
35	---	rs12216891	NA	136127366	C	T	0.97965	0.94627	834.13	376.17	41.661	0.04	0.18351	0.3858	0.053041	3.4977e-13
36	---	chr9:136127382:I	NA	136127382	A	AGCCTC	0.99991	0.015581	1251.9	0.115	0	0	0	ND	ND	ND
37	---	rs13291798	NA	136127481	A	G	0.99227	0.25953	1242.2	9.761	0	0.002	0.0038982	-0.43236	0.39464	0.27327
38	---	rs186319566	NA	136127534	C	A	1	1	1252	0	0	0	0	ND	ND	ND
39	---	rs9411476	NA	136127601	G	A	0.98081	0.95873	741.55	443.51	66.925	0.017	0.23058	0.033088	0.047993	0.49055
40	---	rs34085694	NA	136127605	C	G	0.99157	0.24806	1241.4	10.634	0.003	0.004	0.0042492	-0.49702	0.40463	0.21933
41	---	rs4962113	NA	136127641	T	C	0.90386	0.84096	575.07	543.82	133.01	0.099	0.32345	-0.17926	0.045581	8.3987e-05
42	---	rs112134200	NA	136127689	A	G	0.99849	0.18823	1250.1	1.89	0	0	0.00075479	ND	ND	ND
43	---	rs146579922	NA	136127736	C	T	0.99748	0.11506	1248.8	3.154	0	0	0.0012596	ND	ND	ND
44	---	rs115413527	NA	136127737	G	A	0.98845	0.26706	1236.9	15.091	0	0	0.0060268	-0.14746	0.38746	0.70352
45	---	rs62574563	NA	136127764	C	T	0.96103	0.91922	675.29	484.63	92.006	0.071	0.26704	-0.12629	0.046073	0.0061235
46	---	rs62574564	NA	136127811	G	A	0.96476	0.92562	683.55	482.47	85.92	0.057	0.26132	-0.14733	0.046479	0.001525
47	---	rs73660468	NA	136127907	G	A	0.99996	0.012682	1251.9	0.054	0	0	0	ND	ND	ND
48	---	rs189931088	NA	136127934	C	A	0.99995	0.033943	1251.9	0.058	0	0	0	ND	ND	ND
49	---	rs34039247	NA	136127956	A	C	0.99135	0.23459	1241.2	10.828	0.001	0.001	0.0043251	-0.46352	0.39745	0.24352
50	---	rs10901252	NA	136128000	G	C	0.99201	0.97662	838.99	369.68	43.306	0.017	0.18223	0.37994	0.05184	2.3166e-13
51	---	rs141318395	NA	136128175	G	A	0.99976	0.008487	1251.7	0.299	0	0	0.00011941	ND	ND	ND
52	---	rs62574565	NA	136128259	G	A	0.98307	0.9609	706.68	467.13	78.136	0.054	0.24897	-0.13633	0.046705	0.003511
53	---	rs58081338	NA	136128329	T	C	0.98293	0.96091	705.84	467.9	78.204	0.057	0.24933	-0.1371	0.046684	0.0033167
54	---	rs62574567	NA	136128421	C	G	0.98235	0.95978	706.69	467.16	78.094	0.05	0.24895	-0.13692	0.046709	0.0033756
55	---	rs60484807	NA	136128467	A	G	0.98558	0.97444	425.78	609.68	216.5	0.04	0.41642	0.12501	0.040749	0.0021568
56	---	rs7857390	NA	136128546	A	G	0.97543	0.94996	111.38	510.4	630.16	0.056	0.29281	0.176	0.044343	7.2195e-05
57	---	rs77693339	NA	136128558	T	C	0.99453	0.98277	868.78	348.12	35.085	0.016	0.16705	0.39978	0.053789	1.0667e-13
58	---	rs12554580	NA	136128603	T	C	0.9858	0.97488	425.82	609.68	216.45	0.043	0.41639	0.125	0.040742	0.0021543
59	---	rs12554336	NA	136128663	A	G	0.96931	0.93122	719.48	460.02	72.445	0.053	0.24159	-0.13583	0.047909	0.0045813
60	---	rs12554595	NA	136128664	T	C	0.96954	0.93141	720.32	459.28	72.357	0.049	0.24122	-0.13513	0.047933	0.0048133
61	---	rs11244051	NA	136128731	G	A	0.99583	0.14038	1246.8	5.212	0.003	0	0.0020839	ND	ND	ND
62	---	rs12554339	NA	136128737	A	C	0.98236	0.95916	705.65	467.64	78.678	0.038	0.2496	-0.13505	0.046741	0.003861
63	---	rs10901253	NA	136128772	T	C	0.98353	0.96237	706.03	467.75	78.166	0.047	0.24924	-0.13677	0.046658	0.0033761
64	---	rs10751502	NA	136129079	A	G	0.98545	0.97436	425.26	609.23	217.47	0.042	0.41701	0.12391	0.040704	0.0023338
65	---	rs35698328	NA	136129112	C	T	0.99154	0.18325	1240.6	11.35	0.009	0.007	0.00454	-0.60565	0.48481	0.21157
66	---	rs10793962	NA	136129115	A	T	0.99495	0.98413	868.71	348.23	35.047	0.011	0.16706	0.3993	0.053756	1.1018e-13
67	---	rs11244052	NA	136129125	C	A	0.98427	0.96382	706.97	466.93	78.048	0.053	0.24882	-0.13578	0.046653	0.0036089
68	---	rs11244053	NA	136129360	A	G	0.98434	0.96435	706.28	467.59	78.091	0.038	0.24912	-0.13629	0.046616	0.0034591
69	---	rs4962114	NA	136129611	G	A	0.98237	0.95956	709.96	464.38	77.611	0.05	0.24745	-0.13701	0.046782	0.0034041
70	---	rs4962115	NA	136129642	T	G	0.98471	0.9653	706.4	467.49	78.071	0.04	0.24906	-0.13611	0.046598	0.003491
71	---	rs8176759	NA	136129647	G	A	0.99532	0.98533	868.68	348.31	35.003	0.015	0.16706	0.39884	0.053725	1.1389e-13
72	---	rs1137827	NA	136129715	C	T	0.99537	0.98551	868.67	348.32	34.997	0.015	0.16706	0.3988	0.053722	1.1402e-13
73	---	rs4962116	NA	136129716	G	A	0.9848	0.96551	706.42	467.47	78.062	0.048	0.24905	-0.13606	0.046596	0.0035001
74	---	rs188682222	NA	136129731	G	A	0.99938	0.028384	1251.2	0.776	0	0.001	0.0003099	ND	ND	ND
75	---	rs8176757	NA	136130012	A	C	0.9881	0.97928	426.23	609.66	216.07	0.034	0.41607	0.12499	0.040661	0.0021122
76	---	rs117308290	NA	136130382	G	A	0.99967	0.23179	1251.6	0.411	0	0	0.00016414	ND	ND	ND
77	---	rs62641783	NA	136130653	G	C	0.88545	0.59613	1003.7	236.21	12.038	0.033	0.10395	-0.26212	0.087501	0.0027385
78	---	rs62641784	NA	136130665	G	A	0.91516	0.55494	1077.8	169.01	5.2	0.024	0.07165	-0.29074	0.10768	0.0069306
79	---	rs62641785	NA	136130669	T	C	0.89227	0.71802	888.33	334.16	29.466	0.048	0.15699	-0.20621	0.065714	0.0017009
80	---	rs62641786	NA	136130677	T	C	0.89017	0.66483	963.82	269.33	18.851	0.005	0.12262	-0.22281	0.075894	0.0033264
81	---	rs184116305	NA	136130686	C	G	0.96079	0.4347	1184.9	66.245	0.872	0.001	0.027152	-0.33159	0.204	0.10407
82	---	rs187645401	NA	136130689	A	G	0.93208	0.52788	1119.8	128.06	4.125	0.014	0.054437	-0.08766	0.13053	0.50186
83	---	rs192412098	NA	136130694	C	G	0.94028	0.44951	1150.2	99.31	2.445	0.012	0.041614	-0.24882	0.15583	0.11032
84	---	rs62636487	NA	136130702	C	T	0.93479	0.62416	1086.1	159	6.873	0.018	0.068988	-0.12186	0.10645	0.25229
85	---	rs141472448	NA	136130703	G	A	1	0.0019992	1252	0.002	0	0	0	ND	ND	ND
86	---	rs7469795	NA	136130893	T	C	0.97592	0.96017	468.91	593.26	189.78	0.047	0.38852	0.1473	0.041435	0.00037802
87	---	rs8176751	NA	136131022	C	T	0.99942	0.99823	867.13	349.79	35.078	0.004	0.16771	0.39919	0.05321	6.2797e-14
88	---	chr9:136131056:D	NA	136131056	CG	C	0.99846	0.14736	1250.1	1.921	0	0.001	0.00076717	ND	ND	ND
89	---	rs183748371	NA	136131065	G	A	1	1	1252	0	0	0	0	ND	ND	ND
90	rs8176749	rs8176749	NA	136131188	C	T	1	1	881	338	33	0	0.16134	0.40539	0.053925	5.5758e-14
91	---	rs8176748	NA	136131289	C	T	0.99751	0.99449	710.81	462.39	78.797	0.009	0.2476	-0.14203	0.045885	0.0019658
92	---	rs8176747	NA	136131315	C	G	0.99997	0.99989	867.04	349.96	35	0	0.16772	0.39936	0.053191	6.0051e-14
93	---	rs41302905	NA	136131316	C	T	0.99999	0.0058286	1252	0.012	0	0	0	ND	ND	ND
94	rs8176746	rs8176746	NA	136131322	G	T	1	1	867	350	35	0	0.16773	0.39939	0.053189	5.9587e-14
95	---	rs8176745	NA	136131347	G	A	0.99765	0.99502	710.91	462.38	78.694	0.011	0.24751	-0.14204	0.045882	0.0019637
96	---	rs8176744	NA	136131350	G	T	0.98229	0.29554	1228.2	23.766	0.027	0	0.0095128	-0.43646	0.28641	0.12753
97	---	rs8176743	NA	136131415	C	T	0.9978	0.99462	861.32	352.08	38.607	0	0.17144	0.3825	0.052473	3.1097e-13
98	---	rs56116432	NA	136131429	C	T	1	1	1252	0	0	0	0	ND	ND	ND
99	---	rs8176742	NA	136131437	C	T	0.9935	0.98443	722.51	454.38	75.088	0.021	0.24144	-0.13652	0.046531	0.0033462
100	---	rs8176741	NA	136131461	G	A	0.99775	0.9945	861.25	352.1	38.648	0.001	0.17149	0.38232	0.052465	3.1646e-13
101	---	rs190299333	NA	136131469	G	A	0.99712	0.096925	1248.4	3.601	0	0	0.0014381	ND	ND	ND
102	---	rs8176740	NA	136131472	A	T	0.99776	0.9953	718.08	456.4	77.508	0.009	0.24418	-0.13819	0.045993	0.0026587
103	---	rs8176739	NA	136131523	G	A	0.9999	0.051417	1251.9	0.121	0	0	0	ND	ND	ND
104	---	rs184446112	NA	136131556	G	T	0.99978	0.051382	1251.7	0.272	0	0	0.00010863	ND	ND	ND
105	---	rs55727303	NA	136131576	C	T	0.98865	0.6437	1223.4	27.543	1.08	0	0.011862	0.89465	0.32628	0.0061078
106	---	rs55687199	NA	136131589	C	T	0.99841	0.46096	1248.5	3.488	0	0.002	0.001393	-0.99484	0.76938	0.196
107	---	rs7853989	NA	136131592	G	C	0.99774	0.99454	861.24	352.09	38.676	0	0.1715	0.38223	0.052459	3.1854e-13
108	---	rs1053878	NA	136131651	G	A	0.99077	0.97859	758.22	425.4	68.374	0.01	0.2245	0.06576	0.04745	0.16578
109	---	rs8176737	NA	136131783	C	T	0.99711	0.99388	717.74	456.23	78.024	0.008	0.24452	-0.13799	0.045967	0.0026834
110	---	rs8176736	NA	136131785	T	C	0.99528	0.98945	720.11	454.62	77.263	0.012	0.24327	-0.13993	0.046158	0.0024329
111	---	rs7873416	NA	136131846	T	C	0.99723	0.99538	428.83	606.89	216.26	0.012	0.41511	0.11934	0.04034	0.0030931
112	---	rs7855255	NA	136131895	C	T	0.99772	0.99454	861.19	352.08	38.729	0.001	0.17154	0.38205	0.052448	3.2309e-13
113	---	rs7873522	NA	136131905	T	C	0.99571	0.99233	427.91	606.8	217.29	0.011	0.41589	0.1192	0.040365	0.0031478
114	---	rs114820734	NA	136132010	T	C	0.99727	0.99542	428.86	606.84	216.29	0.007	0.41511	0.11934	0.040338	0.0030917
115	---	rs7873635	NA	136132012	T	C	0.9938	0.98724	110.36	513.6	628.03	0.009	0.29326	0.16915	0.043636	0.00010608
116	---	rs113209447	NA	136132079	C	T	0.9961	0.99181	719.32	456.25	76.416	0.01	0.24325	-0.13917	0.046188	0.0025859
117	---	rs112685218	NA	136132168	T	C	0.99768	0.99454	861.15	352.08	38.777	0	0.17158	0.38188	0.052439	3.2774e-13
118	---	rs181890403	NA	136132205	G	A	0.9998	0.016255	1251.7	0.254	0	0	0.00010144	ND	ND	ND
119	---	rs8176732	NA	136132303	A	G	0.99383	0.98643	716.86	457.83	77.302	0.012	0.24458	-0.14036	0.046138	0.0023482

continued 1

120	---	rs8176731	NA	136132350	T	C	0.99741	0.99567	428.81	606.82	216.35	0.015	0.41515	0.11926	0.040332	0.0031066
121	---	rs2073823	NA	136132516	G	A	0.99758	0.99453	860.98	352.1	38.922	0	0.1717	0.38133	0.052409	3.4373e-13
122	---	rs8176730	NA	136132525	T	C	0.99761	0.99463	860.99	352.07	38.936	0	0.1717	0.3813	0.052406	3.44e-13
123	---	rs8176728	NA	136132561	G	C	0.99785	0.99567	718.21	456.02	77.765	0.005	0.24423	-0.13836	0.045954	0.0026059
124	---	rs8176727	NA	136132570	G	A	0.99823	0.99639	718.31	456.29	77.398	0.008	0.24405	-0.13809	0.045985	0.0026742
125	---	rs112655244	NA	136132608	C	T	0.99795	0.9959	718.35	455.96	77.685	0.007	0.24414	-0.13841	0.045967	0.0026033
126	---	rs8176725	NA	136132617	G	A	0.99526	0.99115	721.82	446.91	83.272	0	0.24499	0.3069	0.045456	1.4632e-11
127	rs2073824	rs2073824	NA	136132633	A	G	1	1	327	609	316	0	0.49561	0.12405	0.039195	0.0015511
128	---	rs2073825	NA	136132707	A	T	0.99883	0.99748	718.55	456.18	77.27	0.001	0.2439	-0.13807	0.045986	0.0026785
129	---	rs8176722	NA	136132754	C	A	0.99867	0.99629	860.62	351.83	39.546	0.001	0.1721	0.37927	0.052282	4.0392e-13
130	---	rs8176721	NA	136132852	G	A	0.99934	0.062922	1251.2	0.82	0	0.001	0.00032748	ND	ND	ND
131	---	rs8176720	NA	136132873	T	C	0.99964	0.9993	428.06	606.21	217.73	0	0.416	0.11869	0.04023	0.0031746
132	---	chr9:136132908:I	NA	136132908	T	TC	0.99875	0.99767	387.31	595.14	269.54	0.002	0.45297	0.23404	0.039133	2.2221e-09
133	---	rs75179845	NA	136132954	T	C	0.99743	0.99301	862.35	350.51	39.138	0.003	0.17124	0.38078	0.05249	4.0388e-13
134	---	rs8176718	NA	136132957	C	T	0.99986	0.99962	718.94	456.01	77.051	0	0.24365	-0.13811	0.045994	0.0026754
135	---	rs8176717	NA	136133034	G	T	0.99933	0.99837	719.63	455.38	76.989	0	0.24335	-0.13799	0.046009	0.0027072
136	---	rs8176716	NA	136133065	G	T	0.99863	0.15653	1250.3	1.712	0	0	0.00068371	ND	ND	ND
137	---	rs8176715	NA	136133148	T	C	0.98802	0.97381	83.13	476.46	692.39	0.02	0.25668	0.15219	0.045798	0.00089066
138	---	rs191976170	NA	136133149	G	A	0.99921	0.24201	1251	0.989	0	0	0.00039497	ND	ND	ND
139	---	rs8176714	NA	136133178	G	A	0.99356	0.98601	648.13	505.84	98.016	0.01	0.2803	-0.16059	0.04443	0.00030096
140	---	rs115166192	NA	136133218	C	T	0.99965	0.0086304	1251.6	0.441	0	0	0.00017612	ND	ND	ND
141	---	rs182985414	NA	136133346	A	C	1	1	1252	0	0	0	0	ND	ND	ND
142	---	chr9:136133380:D	NA	136133380	CGGG	C	0.99867	0.99649	76.608	455.3	720.09	0.004	0.24302	0.13713	0.046111	0.0029396
143	---	rs45610939	NA	136133396	A	G	0.9792	0.8157	1146.1	105.73	0.204	0.003	0.042387	-0.29314	0.10983	0.0076077
144	rs512770	rs512770	NA	136133506	A	G	1	1	77	456	719	0	0.24361	0.1381	0.045998	0.0026784
145	---	rs4962040	NA	136133531	A	G	0.99764	0.99606	629.73	513.26	109.01	0.008	0.29204	-0.168	0.043586	0.000116
146	---	chr9:136133582:D	NA	136133582	AG	A	0.99258	0.98658	91.997	483.41	676.59	0.002	0.26654	0.1278	0.044937	0.0044552
147	---	rs641959	NA	136133699	C	A	0.99758	0.99575	89.25	468.68	694.07	0.001	0.25846	0.13101	0.044934	0.0035502
148	---	rs641943	NA	136133714	G	A	0.99778	0.99624	89.165	468.59	694.24	0.001	0.25836	0.13091	0.044938	0.0035781
149	---	rs514708	NA	136133743	T	C	0.99778	0.99625	89.165	468.59	694.24	0	0.25836	0.13091	0.044938	0.0035785
150	---	rs517414	NA	136134034	A	G	0.9996	0.99919	80.042	459.41	712.54	0	0.2474	0.13739	0.045696	0.0026413
151	---	rs9411372	NA	136134068	G	A	0.97122	0.94646	730.07	444.16	77.763	0.012	0.23949	0.036719	0.047153	0.43615
152	---	rs138164693	NA	136134118	G	A	1	1	1252	0	0	0	0	ND	ND	ND
153	---	rs638756	NA	136134472	C	A	0.99787	0.99641	89.116	468.64	694.24	0	0.25834	0.13085	0.04494	0.0035961
154	---	rs191050088	NA	136134650	C	A	0.99671	0.14604	1247.9	4.122	0	0	0.0016462	ND	ND	ND
155	---	rs183228393	NA	136134835	G	A	1	0.0033309	1252	0.006	0	0	0	ND	ND	ND
156	---	rs626792	NA	136134864	C	A	0.93522	0.87801	103.9	510.59	637.49	0.026	0.2869	0.13795	0.047145	0.0034334
157	---	rs626035	NA	136134994	G	T	0.97273	0.94743	99.363	483.5	669.14	0.006	0.27245	0.14245	0.04496	0.0015325
158	---	rs547495	NA	136134995	C	T	0.97273	0.94743	99.363	483.49	669.14	0.006	0.27245	0.14245	0.04496	0.0015329
159	---	rs547643	NA	136135047	T	C	1	1	80.001	459	713	0	0.2472	0.13752	0.045697	0.0026184
160	---	rs625593	NA	136135096	A	G	0.99998	0.99995	80.002	459.02	712.98	0	0.24721	0.13753	0.045697	0.0026163
161	---	rs8176707	NA	136135108	G	T	0.9361	0.7539	1021.6	214.61	15.814	0.015	0.098338	-0.26212	0.073878	0.00038805
162	---	chr9:136135137:D	NA	136135137	GCTTCTGTCCACAC	G	0.98818	0.97538	85.311	466.49	700.19	0.007	0.25444	0.13855	0.045722	0.0024426
163	---	rs138374634	NA	136135194	G	A	0.99916	0.84615	1246.3	5.664	0	0	0.002262	-0.21306	0.45575	0.64014
164	---	rs549331	NA	136135195	G	C	0.99799	0.99664	89.053	468.71	694.24	0	0.25831	0.13077	0.044942	0.0036164
165	---	rs549443	NA	136135237	A	G	0.89151	0.7955	120.08	528.79	603.05	0.085	0.30711	0.12136	0.047813	0.01114
166	---	rs549446	NA	136135238	T	C	0.88939	0.79851	130.45	531.66	589.83	0.055	0.31653	0.1413	0.046863	0.0025677
167	---	rs149279023	NA	136135321	G	A	1	1	1252	0	0	0	0	ND	ND	ND
168	---	rs624601	NA	136135365	A	G	0.99799	0.99664	89.053	468.71	694.24	0	0.25831	0.13077	0.044942	0.0036164
169	---	rs551322	NA	136135444	G	A	1	1	80	459	713	0	0.2472	0.13752	0.045697	0.0026186
170	---	rs192192638	NA	136135445	C	T	0.96586	0.37961	1202.7	49.017	0.258	0.008	0.019782	-0.21201	0.19199	0.26947
171	---	rs188088103	NA	136135472	C	T	0.99846	0.029421	1250.1	1.927	0	0.002	0.00076957	ND	ND	ND
172	---	rs613423	NA	136135478	A	G	1	1	80	459	713	0	0.2472	0.13752	0.045697	0.0026186
173	---	rs8176704	NA	136135552	G	A	0.99874	0.1339	1250.4	1.574	0	0	0.00062859	ND	ND	ND
174	---	chr9:136135625:D	NA	136135625	CTG	C	1	1	1252	0	0	0	0	ND	ND	ND
175	rs574347	rs574347	NA	136135659	C	T	1	1	80	459	713	0	0.2472	0.13752	0.045697	0.0026186
176	---	rs575259	NA	136135752	G	A	0.99798	0.99664	89.052	468.71	694.24	0.001	0.25831	0.13077	0.044942	0.0036172
177	---	rs8176703	NA	136135863	G	T	0.99894	0.21781	1250.7	1.35	0	0	0.00053914	ND	ND	ND
178	---	rs8176702	NA	136136146	G	A	0.9983	0.99712	630.2	512.79	109.01	0.001	0.29186	-0.16821	0.04356	0.00011268
179	---	rs579483	NA	136136196	A	T	0.99999	0.99998	80	459.01	712.99	0	0.24721	0.13751	0.045697	0.0026198
180	---	rs579622	NA	136136242	A	G	0.99999	0.99997	80	459.01	712.99	0	0.24721	0.13751	0.045697	0.0026199
181	---	rs140166545	NA	136136367	C	T	0.99989	0.058102	1251.9	0.137	0	0	0	ND	ND	ND
182	---	rs493211	NA	136136516	A	G	0.99977	0.9994	79.938	459.06	713	0.002	0.24718	0.13732	0.045716	0.0026667
183	---	rs8176698	NA	136136593	T	C	0.9993	0.065935	1251.1	0.874	0	0	0.00034904	ND	ND	ND
184	---	rs688976	NA	136136770	A	C	0.99996	0.99989	80.015	459.02	712.96	0	0.24722	0.1375	0.045696	0.00262
185	---	rs8176696	NA	136136773	C	T	0.9993	0.065935	1251.1	0.874	0	0	0.00034904	ND	ND	ND
186	---	rs2073826	NA	136136963	G	T	0.99779	0.99642	629.42	513.48	109.1	0.002	0.2922	-0.16751	0.043573	0.00012091
187	---	rs112565602	NA	136137050	G	A	0.9982	0.21075	1249.7	2.259	0	0	0.00090216	ND	ND	ND
188	---	rs687621	NA	136137065	A	G	0.99796	0.99735	378.17	594.14	279.69	0.002	0.46067	0.23505	0.039029	1.7184e-09
189	---	rs184686147	NA	136137088	G	A	0.99996	0.020119	1252	0.044	0	0	0	ND	ND	ND
190	---	rs687289	NA	136137106	G	A	0.99985	0.99973	393.1	591.97	266.92	0	0.44961	0.23365	0.039062	2.2119e-09
191	---	rs2073827	NA	136137133	G	C	1	1	609	527	116	0	0.30312	-0.16228	0.043132	0.00016833
192	rs2073828	rs2073828	NA	136137140	G	A	1	1	609	527	116	0	0.30312	-0.16228	0.043132	0.00016833
193	---	rs189048957	NA	136137228	G	A	0.99997	0.019153	1252	0.036	0	0	0	ND	ND	ND
194	---	rs150021186	NA	136137293	G	A	1	1	1252	0	0	0	0	ND	ND	ND
195	---	rs77513793	NA	136137296	T	C	0.99874	0.38576	1249.6	2.428	0	0	0.00096965	ND	ND	ND
196	---	chr9:136137375:I	NA	136137375	A	AT	1	1	1252	0	0	0	0	ND	ND	ND
197	---	rs55876802	NA	136137547	C	A	1	1	1252	0	0	0	0	ND	ND	ND
198	---	rs143174772	NA	136137600	C	T	1	1	1252	0	0	0	0	ND	ND	ND
199	---	rs151155628	NA	136137641	C	T	1	1	1252	0	0	0	0	ND	ND	ND
200	rs8176694	rs8176694	NA	136137646	T	C	1	1	963	273	16	0	0.12181	-0.2153	0.061315	0.00044578
201	---	rs8176693	NA	136137657	C	T	0.99885	0.99686	867.98	346.46	37.558	0.001	0.16836	0.3894	0.052809	1.6602e-13
202	---	rs673578	NA	136137857	G	T	0.99739	0.99552	88.963	468.74	694.3	0.001	0.25825	0.13014	0.044965	0.003802
203	---	rs8176692	NA	136137937	T	C	0.99811	0.9461	1225.3	25.718	0.996	0.001	0.011066	0.042293	0.18992	0.82378
204	---	rs145090216	NA	136138074	A	G	1	1	1252	0	0	0	0	ND	ND	ND
205	---	rs672316	NA	136138125	G	T	0.99727	0.99545	88.942	468.68	694.38	0.002	0.25821	0.13002	0.044969	0.0038371
206	---	rs8176691	NA	136138229	C	T	0.99753	0.99578	629.41	513.17	109.41	0.002	0.29233	-0.16984	0.04356	9.6637e-05
207	---	rs8176690	NA	136138317	A	G	0.99914	0.99846	608.21	527.37	116.42	0.002	0.3036	-0.16405	0.04314	0.0001431
208	---	rs189530394	NA	136138484	A	C	0.99787	0.18975	1249.3	2.673	0	0	0.0010675	ND	ND	ND
209	---	chr9:136138557:I	NA	136138557	A	AT	0.97855	0.32172	1219	32.845	0.161	0.019	0.013246	1.6807	0.48277	0.00049884
210	---	rs8176688	NA	136138583	C	T	0.99895	0.22039	1250.7	1.332	0	0	0.00053195	ND	ND	ND
211	---	chr9:136138765:D	NA	136138765	GCGCCCACCACTA	G	0.99123	0.93635	1118.2	132.71	1.127	0.007	0.053901	-0.12525	0.092119	0.17393
212	---	rs8176686	NA	136138776	T	C	0.96729	0.93963	624.46	519.27	108.25	0.02	0.29384	-0.16868	0.044658	0.00015861
213	---	rs186003835	NA	136138804	G	A	0.99905	0.092474	1250.8	1.188	0.002	0	0.00047604	ND	ND	ND
214	---	rs8176684	NA	136138874	G	A	0.99907	0.058687	1250.8	1.165	0	0.001	0.00046526	ND	ND	ND
215	---	rs147279040	NA	136138978	C	T	1	1	1252	0	0	0	0	ND	ND	ND
216	---	rs140783133	NA	136139110	A	C	0.99943	0.0989	1251.3	0.714	0	0	0.00028514	ND	ND	ND
217	---	rs147622835	NA	136139238	G	A	0.98897	0.66567	1221	29.835	1.121	0.003	0.01281	0.60711	0.27726	0.02855
218	rs657152	rs657152	NA	136139265	C	A	1	1	400	601	251	0	0.4405	0.24395	0.039492	6.5273e-10
219	---	rs8176682	NA	136139297	C	T	0.99739	0.99546	616.32	520.13	115.55	0.001	0.30001	-0.17379	0.043201	5.7524e-05
220	---	rs139568229	NA	136139383	G	T	1	1	1252	0	0	0	0	ND	ND	ND
221	---	rs149756392	NA	136139395	G	C	1	1	1252	0	0	0	0	ND	ND	ND
222	---	rs145595975	NA	136139460	G	A	0.99811	0.94713	1225.3	25.67	0.998	0.001	0.011049	0.041792	0.18986	0.82578
223	---	rs148824570	NA	136139589	C	A	1	1	1252	0	0	0	0	ND	ND	ND
224	---	rs474279	NA	136139617	T	C	0.99886	0.99784	81.091	458.96	711.95	0	0.24806	0.13477	0.045632	0.0031421
225	---	rs146458069	NA	136139672	C	T	1	1	1252	0	0	0	0	ND	ND	ND
226	---	rs8176765	NA	136139694	A	C	0.9988	0.36712	1249.9	2.121	0	0	0.00084704	ND	ND	ND
227	---	rs191325486	NA	136139702	G	A	0.99482	0.30152	1244.5	7.535	0.001	0	0.00301	1.8613	0.60945	0.0039284
228	---	rs8176681	NA	136139754	T	C	0.99692	0.99523	615.6	520.78	115.62	0.001	0.30033	-0.17307	0.043205	6.1793e-05
229	---	rs144649415	NA	136139775	G	T	0.99323	0.9169	1164.4	83.111	4.491	0.009	0.036779	-0.18025	0.10486	0.085609
230	---	rs141515001	NA	136139833	T	C	1	1	1252	0	0	0	0	ND	ND	ND
231	---	chr9:136139907:D	NA	136139907	GAAACTGCC	G	0.99662	0.9925	649.03	505.2	97.761	0.004	0.27984	0.028133	0.04434	0.52577
232	---	rs186048848	NA	136140035	G	A	0.9977	0.11318	1249.1	2.875	0	0	0.0011482	ND	ND	ND
233	---	rs1633513	NA	136140462	C	T	0.99669	0.99412	90.333	468.89	692.78	0	0.25941	0.12744	0.044872	0.0045111
234	---	rs149567216	NA	136140469	C	T	1	1	1252	0	0	0	0	ND	ND	ND
235	---	rs1633514	NA	136140513	T	C	0.99827	0.99689	81.247	458.84	711.91	0	0.24814	0.13464	0.04563	0.0031697
236	---	rs143159728	NA	136140613	T	G	1	1	1252	0	0	0	0	ND	ND	ND
237	---	rs115325682	NA	136140759	G	A	0.99914	0.4883	1250.1	1.943	0	0	0.00077596	ND	ND	ND
238	---	rs1752339	NA	136141135	C	T	0.99063	0.98026	81.109	449.2	721.69	0.006	0.24418	0.13105	0.045929	0.004325
239	---	rs142141716	NA	136141226	G	C	1	1	1252	0	0	0	0	ND	ND	ND
240	---	rs13299342	NA	136141504	G	A	0.99527	0.98648	872.75	342.77	36.481	0.003	0.16603	0.39461	0.053344	1.3895e-13

continue

continued 2

241	---	rs2519093	NA	136141870	C	T	0.99806	0.99625	647.5	505.95	98.545	0.004	0.28077	0.027212	0.04425	0.53857
242	---	rs144140881	NA	136141877	G	T	1	0.0059976	1252	0.006	0	0	0	ND	ND	ND
243	---	rs113423093	NA	136142000	G	C	0.96439	0.93239	103.39	487.25	661.33	0.03	0.27718	0.12425	0.045443	0.0062532
244	---	rs8176672	NA	136142185	C	T	0.99786	0.99415	874.05	341.66	36.29	0.001	0.16543	0.39692	0.05322	8.7796e-14
245	---	rs514659	NA	136142203	A	C	0.99836	0.99762	397.73	589.42	264.85	0	0.44693	0.23373	0.039049	2.1551e-09
246	---	rs644234	NA	136142217	T	G	0.99837	0.99763	397.73	589.42	264.85	0	0.44693	0.23373	0.039049	2.1556e-09
247	---	rs182705402	NA	136142293	C	T	1	1	1252	0	0	0	0	ND	ND	ND
248	---	rs140796254	NA	136142304	A	T	1	1	1252	0	0	0	0	ND	ND	ND
249	---	rs8176671	NA	136142313	A	C	0.9968	0.99114	874.8	341.48	35.716	0.002	0.1649	0.39753	0.05343	1.0046e-13
250	---	rs143309559	NA	136142339	C	T	1	1	1252	0	0	0	0	ND	ND	ND
251	---	rs643434	NA	136142355	G	A	0.99816	0.99722	397.68	589.26	265.06	0.001	0.44704	0.23399	0.039054	2.0789e-09
252	---	rs8176669	NA	136142463	A	C	0.99711	0.99461	707.9	460.87	83.226	0.005	0.25053	-0.13538	0.045525	0.0029426
253	---	rs148987354	NA	136142465	G	A	0.9999	0.020262	1251.9	0.128	0	0	0	ND	ND	ND
254	---	chr9:136142521:I	NA	136142521	C	CT	0.99487	0.89609	1204.1	47.878	0	0.004	0.019121	-0.0063473	0.15011	0.96627
255	---	chr9:136142540:I	NA	136142540	T	TTG	1	1	1252	0	0	0	0	ND	ND	ND
256	---	rs143746661	NA	136142711	A	G	1	1	1252	0	0	0	0	ND	ND	ND
257	---	rs187821258	NA	136142968	T	C	0.99742	0.11314	1248.8	3.226	0	0	0.0012883	ND	ND	ND
258	---	rs543040	NA	136143000	A	T	0.99582	0.9934	396.75	589.54	265.71	0.001	0.44767	0.2362	0.039159	1.622e-09
259	---	rs146903770	NA	136143055	A	G	0.99111	0.28893	1240.3	11.683	0	0	0.0046657	-0.0046294	0.46627	0.99208
260	---	rs613534	NA	136143120	A	G	0.99583	0.99385	399.27	589.45	263.29	0.001	0.4457	0.23496	0.039102	1.8695e-09
261	---	rs543968	NA	136143121	T	C	0.99437	0.9912	400.49	588.95	262.56	0.002	0.44492	0.23385	0.039169	2.3696e-09
262	---	rs139081859	NA	136143192	G	T	0.99825	0.077245	1249.8	2.196	0	0.001	0.000877	ND	ND	ND
263	---	rs544873	NA	136143212	G	A	0.99502	0.9917	402.45	586.52	263.02	0	0.44432	0.2267	0.03913	6.8917e-09
264	---	rs142956930	NA	136143330	A	G	0.99889	0.098024	1250.6	1.391	0	0	0.00055551	ND	ND	ND
265	---	rs545971	NA	136143372	C	T	0.99838	0.99763	397.73	589.42	264.85	0	0.44693	0.23372	0.039049	2.1595e-09
266	---	rs138194969	NA	136143391	G	A	1	1	1252	0	0	0	0	ND	ND	ND
267	---	rs612169	NA	136143442	A	G	0.99839	0.99764	397.73	589.42	264.85	0	0.44693	0.23373	0.039049	2.158e-09
268	---	rs144566139	NA	136143919	G	A	1	1	1252	0	0	0	0	ND	ND	ND
269	---	chr9:136144000:D	NA	136144000	AT	A	0.98756	0.54188	1223.5	28.432	0.053	0.001	0.011397	0.16805	0.27756	0.54488
270	---	rs8176668	NA	136144059	A	T	0.99815	0.9973	611.89	523.75	116.36	0	0.3021	-0.16333	0.04312	0.00015202
271	---	rs574311	NA	136144110	G	A	0.99799	0.99662	83.583	461.25	707.17	0	0.25096	0.13496	0.045414	0.0029614
272	---	rs187551277	NA	136144172	G	A	0.99825	0.077245	1249.8	2.196	0	0.001	0.000877	ND	ND	ND
273	---	rs597988	NA	136144284	T	A	0.97199	0.95467	459.93	572.81	219.24	0.019	0.40388	0.24214	0.040417	2.0855e-09
274	---	rs597974	NA	136144297	A	G	0.96877	0.94921	462.62	571.57	217.79	0.018	0.40222	0.24298	0.040508	1.9938e-09
275	---	rs140114497	NA	136144308	T	C	0.96718	0.9468	463.77	571.11	217.11	0.017	0.40149	0.24164	0.040583	2.6109e-09
276	---	rs576125	NA	136144309	G	A	0.96718	0.94681	463.77	571.1	217.12	0.017	0.4015	0.24163	0.040582	2.6158e-09
277	---	rs8176663	NA	136144427	T	C	0.99836	0.99757	397.61	589.5	264.89	0	0.447	0.23358	0.039052	2.2144e-09
278	---	rs8176662	NA	136144454	A	G	0.99669	0.9908	874.92	341.45	35.619	0.004	0.16481	0.39785	0.053453	9.8386e-14
279	---	rs149899707	NA	136144521	C	T	1	1	1252	0	0	0	0	ND	ND	ND
280	---	rs488775	NA	136144534	G	A	0.9979	0.99644	83.591	461.14	707.27	0.001	0.25093	0.13473	0.045419	0.0030144
281	---	rs66697526	NA	136144593	G	T	0.99595	0.98605	958.35	272.93	20.717	0.007	0.12554	-0.02537	0.060261	0.67375
282	---	rs7036642	NA	136144626	G	A	0.9981	0.99718	611.96	523.69	116.36	0	0.30208	-0.16337	0.043123	0.00015164
283	---	rs8176661	NA	136144640	A	G	0.99996	0.0086711	1252	0.045	0	0	0	ND	ND	ND
284	---	rs596141	NA	136144689	T	G	0.99816	0.99701	79.694	461.37	710.94	0	0.24791	0.1373	0.045782	0.002708
285	---	rs146907040	NA	136144771	A	G	1	0.0039984	1252	0.004	0	0	0	ND	ND	ND
286	---	rs491626	NA	136144873	C	T	0.99109	0.98649	411.75	583.83	256.42	0.001	0.43797	0.19894	0.039186	3.8392e-07
287	---	rs492488	NA	136144960	G	A	0.99656	0.99462	391.81	591.2	269	0	0.45095	0.2331	0.039058	2.4003e-09
288	---	rs493246	NA	136144994	G	A	0.98929	0.982	384.57	593.12	274.3	0.01	0.45596	0.23485	0.039266	2.2184e-09
289	---	rs193238931	NA	136145029	C	T	0.99979	0.10628	1251.7	0.261	0	0	0.00010423	ND	ND	ND
290	---	rs494242	NA	136145118	C	T	0.99587	0.99328	391.13	591.58	269.29	0.001	0.45134	0.23314	0.039088	2.4534e-09
291	---	rs495203	NA	136145240	C	T	0.99587	0.99328	391.13	591.58	269.29	0.001	0.45134	0.23314	0.039088	2.4537e-09
292	---	chr9:136145403:D	NA	136145403	AC	A	0.89421	0.82553	562.28	557.59	132.08	0.05	0.32819	0.20554	0.047103	1.2795e-05
293	---	chr9:136145414:I	NA	136145414	C	CA	0.90476	0.60485	1022.7	217.57	11.653	0.032	0.096197	0.37574	0.085683	1.1587e-05
294	---	chr9:136145418:D	NA	136145418	AC	A	0.88378	0.80486	584.03	533.39	134.53	0.044	0.32048	0.17883	0.047069	0.00014515
295	---	chr9:136145424:D	NA	136145424	AC	A	0.96664	0.92861	684.01	483.64	84.323	0.03	0.2605	0.033456	0.047087	0.47739
296	---	rs9411378	NA	136145425	C	A	0.96466	0.92381	684.98	482.46	84.528	0.038	0.2602	0.035049	0.047122	0.457
297	---	rs582118	NA	136145471	A	G	0.99785	0.9965	392.39	591.52	268.09	0	0.45036	0.23391	0.03908	2.1568e-09
298	---	rs582094	NA	136145484	A	T	0.99785	0.9965	392.39	591.52	268.09	0	0.45036	0.23391	0.03908	2.1568e-09
299	---	rs189022067	NA	136145717	A	G	0.99986	0.035747	1251.8	0.179	0	0	0	ND	ND	ND
300	---	chr9:136145907:I	NA	136145907	G	GA	0.99609	0.99346	395.16	590.05	266.79	0.001	0.44874	0.23502	0.039071	1.7968e-09
301	---	rs2769071	NA	136145974	A	G	0.99601	0.99357	392.38	591.18	268.44	0.004	0.4505	0.23674	0.039157	1.4849e-09
302	---	rs147027134	NA	136145979	A	G	1	1	1252	0	0	0	0	ND	ND	ND
303	---	rs677355	NA	136146046	G	A	0.99691	0.99484	394.01	590.7	267.29	0.003	0.44939	0.23489	0.039079	1.8488e-09
304	---	chr9:136146061:I	NA	136146061	T	TA	0.99429	0.99034	398.01	585.8	268.18	0.009	0.44815	0.23506	0.039103	1.8422e-09
305	---	chr9:136146068:I	NA	136146068	A	AT	0.99726	0.99542	393.77	590.73	267.49	0.005	0.44957	0.2346	0.03907	1.9183e-09
306	---	rs676996	NA	136146077	T	G	0.99628	0.99354	399.35	585.54	267.11	0.005	0.44719	0.23275	0.039007	2.4183e-09
307	---	rs676457	NA	136146227	A	T	0.99785	0.99652	393.66	590.84	267.5	0.002	0.44962	0.23446	0.039059	1.9414e-09
308	---	rs79343853	NA	136146310	T	C	0.99653	0.99042	867.38	349.01	35.609	0.006	0.16782	0.39565	0.05327	1.1091e-13
309	---	rs527210	NA	136146431	C	T	0.99668	0.99439	399.33	585.7	266.97	0.001	0.44714	0.23232	0.039029	2.6396e-09
310	---	chr9:136146448:D	NA	136146448	TAAGAC	T	0.98769	0.98377	406.78	582.92	262.29	0.011	0.44229	0.23031	0.039126	3.9473e-09
311	---	rs675201	NA	136146466	A	G	0.99409	0.98811	82.173	466.32	703.51	0.005	0.25186	0.1329	0.045804	0.0037135
312	---	rs182739868	NA	136146499	A	G	0.91465	0.6591	1001.1	240.33	10.487	0.045	0.10436	0.16996	0.082419	0.039189
313	---	rs550057	NA	136146597	C	T	0.99831	0.9969	646.08	506.91	99.016	0.001	0.28153	0.025541	0.044212	0.56347
314	---	rs674302	NA	136146664	T	A	0.99861	0.99795	393.03	591.17	267.8	0	0.44999	0.23382	0.03905	2.1272e-09
315	---	rs551100	NA	136146740	T	C	0.99788	0.99613	79.842	461.53	710.62	0.001	0.24809	0.13739	0.045783	0.0026921
316	---	rs187072196	NA	136146756	C	A	0.99994	0.016025	1251.9	0.074	0	0	0	ND	ND	ND
317	---	rs191709829	NA	136146850	C	T	0.9983	0.71163	1245.5	6.497	0	0	0.0025946	0.89388	0.43059	0.037899
318	---	rs663054	NA	136146920	T	G	0.99796	0.99619	79.871	461.51	710.61	0.002	0.24811	0.13736	0.045779	0.0026948
319	---	rs7046674	NA	136147012	C	T	0.99716	0.99473	612.89	523.29	115.82	0.004	0.30149	-0.16392	0.043205	0.00014828
320	---	rs554833	NA	136147160	C	T	0.99889	0.99822	393.07	591.1	267.83	0.001	0.44998	0.23369	0.039045	2.1623e-09
321	---	rs8176649	NA	136147295	G	A	0.99727	0.99545	634.51	508.13	109.36	0	0.29028	-0.1689	0.043519	0.00010398
322	---	rs141837362	NA	136147453	T	C	0.99977	0.29192	1251.7	0.292	0	0	0.00011661	ND	ND	ND
323	---	rs144458168	NA	136147539	T	C	1	1	1252	0	0	0	0	ND	ND	ND
324	---	rs660340	NA	136147553	A	G	0.99831	0.99728	270.49	597.73	383.79	0	0.45475	0.22847	0.039206	5.6276e-09
325	---	chr9:136147630:I	NA	136147630	G	GA	0.99992	0.081283	1251.9	0.1	0	0	0	ND	ND	ND
326	---	rs148402540	NA	136147666	A	G	1	1	1252	0	0	0	0	ND	ND	ND
327	---	rs581107	NA	136147702	C	T	0.99615	0.9931	265.48	596.95	389.57	0.002	0.45044	0.23367	0.039303	2.7612e-09
328	---	rs659104	NA	136147823	T	G	0.99848	0.99754	270.5	597.71	383.79	0	0.45475	0.22838	0.039203	5.6891e-09
329	---	rs150947224	NA	136147988	T	C	1	0.0009996	1252	0.001	0	0	0	ND	ND	ND
330	---	rs647800	NA	136148000	A	G	0.99857	0.9977	270.5	597.71	383.79	0.002	0.45475	0.22833	0.039201	5.7283e-09
331	---	rs473533	NA	136148035	T	C	0.99828	0.99718	270.49	597.99	383.52	0.004	0.45486	0.22862	0.039211	5.5298e-09
332	---	rs475419	NA	136148231	C	T	0.99867	0.99785	270.55	597.66	383.79	0	0.45478	0.22827	0.039197	5.7596e-09
333	---	rs476410	NA	136148368	G	C	0.99854	0.99765	270.52	597.98	383.5	0.001	0.45488	0.22849	0.039205	5.608e-09
334	---	rs645982	NA	136148409	A	G	0.99883	0.99816	270.5	597.69	383.8	0	0.45475	0.22818	0.039196	5.8326e-09
335	rs500498	rs500498	NA	136148647	T	C	1	1	271	598	383	0	0.45527	0.22834	0.039162	5.5207e-09
336	---	rs500499	NA	136148648	G	C	0.99776	0.99613	276.7	607.35	367.95	0.004	0.46356	0.23474	0.039386	2.5227e-09
337	---	rs149135480	NA	136148703	T	A	0.99985	0.018738	1251.8	0.193	0	0	0	ND	ND	ND
338	---	rs143292918	NA	136148765	G	A	1	0.0009996	1252	0.001	0	0	0	ND	ND	ND
339	---	chr9:136149095:D	NA	136149095	CA	C	0.98795	0.97957	417.68	594.87	239.43	0.016	0.42881	0.25352	0.040146	2.7024e-10
340	---	rs116552240	NA	136149098	T	A	0.98795	0.97959	417.68	594.87	239.43	0.014	0.42881	0.25351	0.040146	2.706e-10
341	---	rs8176644	NA	136149150	C	T	0.99735	0.99242	867.67	349.02	35.312	0.003	0.16759	0.39635	0.053303	1.0397e-13
342	---	rs188675313	NA	136149152	T	C	0.99998	0.005944	1252	0.021	0	0	0	ND	ND	ND
343	rs505922	rs505922	NA	136149229	T	C	1	1	393	590	269	0	0.45048	0.23278	0.038988	2.365e-09
344	---	rs507666	NA	136149399	G	A	0.9981	0.99627	647.06	505.68	99.258	0.002	0.28123	0.025847	0.044193	0.55865
345	---	rs186959044	NA	136149400	G	A	0.9973	0.31509	1247.8	4.211	0	0	0.0016817	-0.1227	0.73252	0.86698
346	---	rs529565	NA	136149500	T	C	0.99996	0.99991	392.98	591.02	268	0	0.45009	0.23294	0.039026	2.3901e-09
347	---	rs630510	NA	136149581	A	G	0.99927	0.99863	170.56	545.98	535.46	0.001	0.35427	0.11153	0.040624	0.006045
348	---	chr9:136149709:D	NA	136149709	AC	A	0.99594	0.97136	1110.5	140.35	1.126	0.002	0.056949	-0.14608	0.08835	0.098236
349	rs630014	rs630014	NA	136149722	A	G	1	1	171	546	535	0	0.35463	0.11181	0.040566	0.005846
350	---	rs532436	NA	136149830	G	A	0.99808	0.99626	647.09	505.65	99.259	0.004	0.28122	0.025874	0.044193	0.55822
351	---	rs8176639	NA	136150317	C	G	0.99965	0.08622	1251.6	0.434	0	0	0.00017332	ND	ND	ND
352	---	rs8176760	NA	136150379	G	A	0.9827	0.22758	1229.2	22.622	0.129	0.006	0.0091374	-0.75191	0.3237	0.020185
353	---	rs537895	NA	136150403	A	G	0.97813	0.95692	97.54	473.04	681.38	0.036	0.26683	0.13036	0.044824	0.003634
354	---	rs616154	NA	136150466	C	T	0.98953	0.98563	430.7	595.98	225.31	0.004	0.41798	0.060749	0.04004	0.12921
355	---	rs559723	NA	136150484	A	G	0.9905	0.98559	433.68	593.65	224.67	0.009	0.41653	0.057549	0.040098	0.15123
356	---	rs144289739	NA	136150522	C	G	0.99782	0.50843	1248.3	3.724	0	0	0.0014872	0.036687	0.58432	0.94994
357	---	rs8176638	NA	136150556	C	T	0.9655	0.54407	1177.3	73.253	1.443	0.024	0.030408	0.25521	0.15608	0.10201
358	---	rs140079020	NA	136150809	C	T	0.99889	0.078057	1250.6	1.388	0	0.001	0.00055431	ND	ND	ND
359	---	chr9:136150912:I	NA	136150912	C	CG	0.99999	0.0096102	1252	0.013	0	0	0	ND	ND	ND
360	---	rs142995738	NA	136151065	C	G	0.99533	0.69746	1237.1	14.878	0.002	0.001	0.0059433	0.25523	0.34052	0.45355
361	---	rs190049657	NA	136151107	C	T	0.99998	0.004605	1252	0.026	0	0	0	ND	ND	ND

continue

continued 3

362	---	rs566183	NA	136151198	A	G	0.99981	0.9996	80.003	460.79	711.21	0	0.24792	0.13644	0.045699	0.0028311
363	rs568203	rs568203	NA	136151445	A	C	1	1	80	461	711	0	0.248	0.13668	0.045696	0.0027801
364	---	rs600038	NA	136151806	T	C	0.99859	0.99702	647.51	505.09	99.397	0.005	0.28111	0.026237	0.044172	0.55254
365	---	rs8176635	NA	136152009	G	A	0.99781	0.99572	509.3	574.27	168.43	0	0.36387	0.093837	0.041158	0.022611
366	---	rs8176634	NA	136152070	G	C	0.9989	0.99671	822.29	369.72	59.983	0.002	0.19556	-0.20037	0.048669	3.8398e-05
367	---	rs150185195	NA	136152286	C	T	0.98223	0.82631	1155.9	96.035	0.055	0.005	0.038397	-0.26446	0.11394	0.020284
368	---	rs146783421	NA	136152336	A	C	1	0.003398	1252	0.005	0	0	0	ND	ND	ND
369	---	rs182406156	NA	136152347	C	T	0.99998	0.0061435	1252	0.026	0	0	0	ND	ND	ND
370	---	rs8176633	NA	136152354	C	T	0.9965	0.99222	800.55	385.48	65.975	0	0.20664	-0.19025	0.047796	6.8784e-05
371	---	rs145690885	NA	136152368	G	A	0.97486	0.40551	1210.5	41.39	0.098	0.004	0.016608	-0.090541	0.22581	0.68845
372	---	rs8176632	NA	136152547	C	T	0.99758	0.99186	867.35	348.72	35.923	0.005	0.16796	0.39646	0.053182	8.9986e-14
373	---	rs8176631	NA	136152594	C	T	0.99974	0.041108	1251.7	0.321	0	0	0.00012819	ND	ND	ND
374	---	rs8176630	NA	136152722	C	T	0.99683	0.98398	1023.4	219.58	9.029	0.003	0.094904	-0.02347	0.069032	0.73387
375	---	rs188389911	NA	136153355	G	A	0.99981	0.03951	1251.8	0.242	0	0	0	ND	ND	ND
376	---	rs663367	NA	136153451	A	G	0.99777	0.99649	81.231	464.11	706.66	0	0.25023	0.13739	0.045667	0.0026254
377	---	rs552148	NA	136153481	T	C	0.9846	0.97189	85.345	472.52	694.13	0.001	0.25687	0.1407	0.045575	0.0020211
378	---	rs61696148	NA	136153544	A	T	0.99658	0.98329	1026	216.96	9.047	0.001	0.093873	-0.023778	0.069321	0.73159
379	---	rs141638449	NA	136153797	C	T	0.99874	0.036203	1250.4	1.576	0	0	0.00062939	ND	ND	ND
380	---	rs651007	NA	136153875	C	T	0.99996	0.9999	647.01	504.99	100	0	0.28155	0.025238	0.044093	0.56707
381	---	rs11244061	NA	136153981	C	T	0.99201	0.57166	1234.7	17.302	0	0	0.0069097	-0.35008	0.27863	0.20896
382	---	rs187861744	NA	136154097	G	C	0.99827	0.51738	1248.4	3.554	0	0	0.0014193	-0.081551	0.63951	0.89853
383	---	rs579459	NA	136154168	T	C	0.99997	0.99993	647.01	504.99	100	0	0.28155	0.025236	0.044093	0.56709
384	---	rs9411488	NA	136154246	G	T	0.99751	0.99417	805.67	381.75	64.577	0	0.20404	-0.198	0.047976	3.6737e-05
385	---	rs649129	NA	136154304	C	T	0.99998	0.99995	647.01	504.99	100.01	0	0.28155	0.025229	0.044092	0.5672
386	---	rs10751504	NA	136154433	G	C	0.9682	0.93236	708.84	473.58	69.535	0.044	0.24468	0.3069	0.048743	3.0492e-10
387	---	chr9:136154623:D	NA	136154623	TC	T	0.99812	0.12397	1249.6	2.357	0	0	0.00094129	ND	ND	ND
388	---	chr9:136154626:D	NA	136154626	CA	C	0.99812	0.12397	1249.6	2.357	0	0	0.00094129	ND	ND	ND
389	---	rs147375567	NA	136154866	A	G	0.99898	0.50891	1249.9	2.111	0	0	0.00084305	-0.36094	0.78396	0.64523
390	rs495828	rs495828	NA	136154867	G	T	1	1	647	505	100	0	0.28155	0.025236	0.044092	0.56709
391	---	rs635634	NA	136155000	C	T	0.99998	0.99995	647.01	504.98	100.01	0	0.28155	0.025221	0.044092	0.56731
392	---	rs12683493	NA	136155063	C	T	0.99649	0.98298	1026	216.93	9.046	0.003	0.093858	-0.023794	0.069336	0.73147
393	---	rs150022816	NA	136155093	G	T	1	1	1252	0	0	0	0	ND	ND	ND
394	---	rs13298002	NA	136155127	G	A	0.99811	0.12465	1249.6	2.365	0	0	0.00094449	ND	ND	ND
395	rs500428	rs500428	NA	136155343	A	G	1	1	90	474	688	0	0.26118	0.1299	0.044787	0.0037263
396	---	rs7030248	NA	136155359	G	A	0.99743	0.99482	692.55	484.83	74.628	0	0.25323	0.29602	0.046352	1.6984e-10
397	---	rs9411490	NA	136155414	A	G	0.99744	0.99405	805.68	381.77	64.549	0.003	0.20402	-0.19798	0.047982	3.6893e-05
398	---	rs633862	NA	136155444	C	T	0.99743	0.99608	279.07	606.76	366.17	0.002	0.46522	0.23387	0.039386	2.8863e-09
399	---	rs502361	NA	136155589	C	G	0.99994	0.99985	89.994	473.97	688.04	0	0.26116	0.12993	0.044789	0.0037215
400	---	rs9411491	NA	136156015	T	C	0.99715	0.99328	805.67	381.78	64.553	0.004	0.20403	-0.19793	0.047995	3.7235e-05
401	---	rs55988407	NA	136156064	G	A	0.9809	0.47025	1218	33.835	0.149	0.003	0.013631	-0.26652	0.23535	0.25745
402	---	rs9411381	NA	136156067	T	C	0.99683	0.99246	805.65	381.78	64.556	0.017	0.20403	-0.19784	0.04801	3.7758e-05
403	---	rs78590974	NA	136156230	C	T	0.98471	0.47114	1226.6	25.202	0.241	0.002	0.010257	1.0622	0.29539	0.00032328
404	---	rs142447342	NA	136156244	G	A	1	1	1252	0	0	0	0	ND	ND	ND
405	---	rs140502769	NA	136156498	G	A	0.99999	0.0094373	1252	0.018	0	0	0	ND	ND	ND
406	---	rs144306320	NA	136156542	G	T	0.999	0.013524	1250.7	1.255	0	0.001	0.0005012	ND	ND	ND
407	---	rs189611925	NA	136156672	G	T	0.9998	0.016521	1251.8	0.246	0	0	0	ND	ND	ND
408	---	rs557317	NA	136157037	C	A	0.98391	0.9689	97.661	488.34	665.97	0.026	0.27303	0.12273	0.044781	0.0061314
409	---	rs558240	NA	136157133	G	A	0.99025	0.97154	895.23	333.18	23.573	0.012	0.15189	-0.076444	0.057078	0.18047
410	---	rs138026250	NA	136157138	C	A	0.99982	0.13924	1251.8	0.224	0	0	0	ND	ND	ND
411	---	rs146149084	NA	136157335	A	G	0.99998	0.011513	1252	0.023	0	0	0	ND	ND	ND
412	---	rs181316261	NA	136157449	C	T	0.99919	0.14459	1251	1.009	0	0	0.00040296	ND	ND	ND
413	---	rs7867220	NA	136157520	C	T	0.99949	0.11798	1251.4	0.64	0	0	0.00025559	ND	ND	ND
414	---	rs183796796	NA	136157596	C	T	0.99988	0.032044	1251.8	0.154	0	0	0	ND	ND	ND
415	---	rs142673851	NA	136157644	C	T	0.9994	0.14465	1251.3	0.746	0	0	0.00029792	ND	ND	ND
416	---	rs62574607	NA	136157654	T	C	0.99069	0.87367	1181.9	67.059	3.032	0.002	0.029203	0.040896	0.12094	0.73525
417	---	rs7867359	NA	136157656	C	G	0.99945	0.12814	1251.3	0.687	0	0	0.00027436	ND	ND	ND
418	---	rs7853492	NA	136157785	A	T	0.99945	0.14681	1251.3	0.69	0	0	0.00027556	ND	ND	ND
419	---	rs7867739	NA	136157924	C	T	0.99942	0.15659	1251.3	0.726	0	0	0.00028994	ND	ND	ND
420	---	rs7867864	NA	136158008	C	G	0.99936	0.15691	1251.2	0.8	0	0	0.00031949	ND	ND	ND
421	---	rs141952207	NA	136158026	G	C	0.99996	0.008981	1252	0.048	0	0	0	ND	ND	ND
422	---	rs149863406	NA	136158155	C	T	0.99918	0.013745	1251	1.032	0	0	0.00041214	ND	ND	ND
423	---	rs28476494	NA	136158288	T	C	0.99928	0.18331	1251.1	0.9	0	0	0.00035942	ND	ND	ND
424	---	rs7860182	NA	136158327	T	A	0.98662	0.97544	98.485	491.58	661.91	0.02	0.27499	0.13299	0.044596	0.0028616
425	---	rs28368280	NA	136158400	C	G	0.99925	0.1935	1251.1	0.942	0	0	0.0003762	ND	ND	ND
426	---	rs184855103	NA	136158490	C	T	0.99998	0.0090575	1252	0.029	0	0	0	ND	ND	ND
427	---	rs1927311	NA	136158544	T	C	0.9874	0.97617	104.32	494.88	652.79	0.01	0.28096	0.11552	0.044112	0.0088235
428	---	rs115071869	NA	136158862	G	A	0.99911	0.23694	1250.9	1.112	0	0	0.00044409	ND	ND	ND
429	---	rs507480	NA	136158964	A	G	0.99937	0.989	3	59.69	1189.3	0.001	0.026234	-0.049716	0.12077	0.68059
430	---	rs507567	NA	136158998	A	G	0.99705	0.38212	0.015	5.06	1246.9	0.003	0.0020328	0.47522	0.6297	0.45045
431	---	rs509482	NA	136159240	G	A	0.98604	0.96899	724.2	454.3	73.468	0.029	0.24012	-0.15394	0.047072	0.0010746
432	---	rs62574608	NA	136159266	A	G	0.92354	0.50729	1110.6	135.9	5.479	0.02	0.058652	-0.089473	0.12863	0.48669
433	---	chr9:136159268:D	NA	136159268	AAGGCC	A	0.92738	0.62775	1063.2	181.65	7.148	0.013	0.078252	-0.28584	0.096591	0.003084
434	---	rs146582653	NA	136159450	G	C	0.99949	0.4148	1251.3	0.684	0	0	0.00027316	ND	ND	ND
435	---	rs67318122	NA	136159538	A	G	0.9957	0.27916	1246.6	5.365	0.013	0	0.002153	-0.064268	0.66418	0.92291
436	---	rs512179	NA	136159540	C	G	0.99989	0.9997	703.95	467.03	81.018	0.001	0.25122	-0.13479	0.045602	0.0031194
437	---	rs512887	NA	136159579	G	A	0.9999	0.99973	703.96	467.03	81.016	0	0.25122	-0.1348	0.045602	0.003117
438	---	rs535855	NA	136159790	A	G	0.99996	0.99988	703.98	467.01	81.008	0	0.25121	-0.13484	0.0456	0.0031066
439	---	rs535993	NA	136159843	A	G	0.99996	0.99989	703.98	467.01	81.007	0	0.25121	-0.13484	0.0456	0.0031063
440	---	chr9:136159892:I	NA	136159892	T	TAA	0.90366	0.70227	949	287.24	15.747	0.016	0.12729	-0.048454	0.069318	0.48454
441	---	chr9:136159896:I	NA	136159896	T	TAAAAGAAATAA	0.90474	0.70343	954.66	281.97	15.353	0.016	0.12487	-0.055147	0.06979	0.42942
442	---	rs184760764	NA	136160131	G	A	0.999	0.023915	1250.7	1.254	0	0	0.0005008	ND	ND	ND
443	---	rs146174278	NA	136160165	G	A	0.99934	0.062768	1251.2	0.822	0	0	0.00032827	ND	ND	ND
444	---	rs1752338	NA	136160185	C	A	1	1	704	467	81	0	0.2512	-0.13488	0.045599	0.0030973
445	---	rs1633512	NA	136160219	T	C	1	1	704	467	81	0	0.2512	-0.13488	0.045599	0.0030973
446	rs1752337	rs1752337	NA	136160228	T	G	1	1	704	467	81	0	0.2512	-0.13488	0.045599	0.0030973
447	---	rs62574609	NA	136160311	T	C	1	1	704	467	81	0	0.2512	-0.13488	0.045599	0.0030973
448	---	rs62574610	NA	136160481	C	T	1	1	704	467	81	0	0.2512	-0.13488	0.045599	0.0030973
449	---	rs62574611	NA	136160538	G	C	1	1	704	467	81	0	0.2512	-0.13488	0.045599	0.0030973
450	---	rs9411492	NA	136160617	T	C	0.98128	0.97184	287.87	612.33	351.77	0.02	0.47448	0.08636	0.039936	0.030583
451	---	rs565126	NA	136160632	G	A	0.99638	0.9922	701.88	468.47	81.647	0.004	0.2523	-0.13283	0.04569	0.0036479
452	---	chr9:136160859:I	NA	136160859	C	CA	0.90639	0.83416	110.21	546.6	595.11	0.084	0.30634	0.016116	0.047749	0.73573
453	---	rs567898	NA	136160931	A	G	1	1	704	467	81	0	0.2512	-0.13488	0.045599	0.0030973
454	---	rs568022	NA	136160975	G	C	0.95943	0.90869	745.5	442.23	64.26	0.012	0.22794	-0.14115	0.050231	0.0049545
455	---	rs568975	NA	136161074	G	A	1	1	704	467	81	0	0.2512	-0.13488	0.045599	0.0030973
456	---	rs9411382	NA	136161274	T	C	0.99999	0.99999	81.005	467	704	0	0.2512	0.13488	0.045599	0.0030967
457	---	rs572651	NA	136161469	C	T	0.99998	0.99994	704	466.97	81.027	0	0.25121	-0.13486	0.045596	0.0030987
458	---	rs572653	NA	136161472	A	G	1	1	704	467	81	0	0.2512	-0.13488	0.045599	0.0030967
459	---	rs139740994	NA	136161579	G	C	0.99976	0.030689	1251.7	0.296	0	0	0.00011821	ND	ND	ND
460	---	rs11244064	NA	136161837	G	A	0.98501	0.96369	803.78	411.49	36.723	0.017	0.19367	0.018034	0.052617	0.73179
461	rs9411493	rs9411493	NA	136161851	A	G	1	1	102	491	659	0	0.27756	0.11794	0.043869	0.0071757
462	---	chr9:136161928:D	NA	136161928	TTTC	T	0.99708	0.95666	1186.6	62.174	3.211	0.004	0.027395	0.093779	0.12119	0.43903
463	---	rs176689	NA	136162131	G	T	1	1	704	467	81	0	0.2512	-0.13488	0.045599	0.0030967
464	---	rs68032997	NA	136162255	T	C	0.97739	0.92826	937.61	289.88	24.48	0.03	0.13532	-0.0038677	0.059862	0.94848
465	---	rs176690	NA	136162764	C	T	0.99896	0.99794	705.11	466.17	80.716	0.001	0.25064	-0.13381	0.045652	0.0033772
466	---	rs117653144	NA	136162783	G	A	0.99704	0.96021	1197.7	51.31	3	0.001	0.022887	0.063115	0.13028	0.62807
467	---	rs176691	NA	136163306	G	A	0.98172	0.95706	798.88	415.98	37.115	0.02	0.19578	0.017496	0.052644	0.73963
468	---	rs176692	NA	136163329	A	G	0.98149	0.95645	799.21	415.7	37.067	0.017	0.19562	0.017179	0.052671	0.7443
469	---	rs10901256	NA	136163447	T	C	0.97309	0.95884	317.58	609.39	325	0.034	0.49704	0.096185	0.040216	0.01677
470	---	rs10901257	NA	136163570	G	A	0.98239	0.97356	286.85	612.97	352.16	0.022	0.47392	0.087834	0.039922	0.027796
471	---	rs176693	NA	136164001	C	T	1	0.99999	704	467	81	0	0.2512	-0.13488	0.045599	0.0030974
472	---	rs150942415	NA	136164037	G	A	0.99995	0.012319	1251.9	0.064	0	0	0	ND	ND	ND
473	---	rs140667476	NA	136164095	G	T	0.99989	0.01535	1251.9	0.134	0	0	0	ND	ND	ND
474	---	rs28844968	NA	136164427	A	G	0.96493	0.8298	1053.2	192.58	6.205	0.009	0.081864	-0.27585	0.079635	0.00053222
475	---	rs176694	NA	136164593	T	G	0.99808	0.28579	1249.4	2.572	0	0	0.0010272	ND	ND	ND
476	---	rs176695	NA	136164639	A	G	1	0.99999	704	467	81	0	0.2512	-0.13488	0.045599	0.0030978
477	---	rs176696	NA	136164655	C	T	0.99976	0.9994	102.08	491.12	658.8	0.002	0.27767	0.11781	0.043874	0.0072483
478	---	rs13301309	NA	136164836	C	A	0.99571	0.2919	1246.6	5.36	0.013	0	0.002151	-0.066651	0.66737	0.92045
479	---	rs191901590	NA	136164922	T	G	1	1	1252	0	0	0	0	ND	ND	ND
480	---	rs176697	NA	136165211	T	C	0.99859	0.99719	101.56	490.13	660.31	0.005	0.27686	0.12035	0.04398	0.0062101
481	---	rs193010087	NA	136165284	C	T	0.9993	0.14647	1251.1	0.871	0	0	0.00034784	ND	ND	ND
482	---	rs2519836	NA	136165347	T	C	1	0.0009996	0	0.001	1252	0	0	ND	ND	ND
483	---	rs182507498	NA	136165348	G	A	0.99422	0.22194	1243.9	8.078	0	0.001	0.003226	-0.21131	0.48948	0.66595
484	---	rs176698	NA	136165371	A	C	0.99999	0.99999	704	467	81	0	0.2512	-0.13488	0.045599	0.003098
485	---	rs145834222	NA	136165436	C	A	0.99951	0.44019	1251.3	0.726	0	0	0.00028994	ND	ND	ND
486	---	rs187543752	NA	136165446	C	T	1	1	1252	0	0	0	0	ND	ND	ND
487	---	rs7020432	NA	136165694	G	A	0.99559	0.28675	1246.5	5.505	0.014	0	0.0022097	-0.061356	0.65179	0.925
488	rs7025162	rs7025162	NA	136166346	C	T	1	1	444	576	232	0	0.41534	-0.074571	0.03928	0.057639

* id '---' indicate that these variants were extraporated.

Table S2. Associations of imputed genotypes in the ABOlocus with ACE in Takahata population.

					Allele		average maximum		Genotypes							
index	id*	rsid	chr	pos	A	B	_posterior call	info	AA	AB	BB	Null	MAF	beta	SE	P-value
1	---	rs73562368	17	136123019	C	T	0.99972	0.0585888	1251.6	0.351	0	0	0.00014018	ND	ND	ND
2	---	rs11244049	17	136123092	T	C	0.99546	0.98526	907.73	314.42	29.845	0.008	0.14941	0.37435	0.05596	2.2379e-11
3	---	rs148031614	17	136123172	T	C	0.99928	0.25135	1251.1	0.9	0	0	0.00035942	ND	ND	ND
4	---	rs9328546	17	136123267	C	G	0.93562	0.84338	838.89	376.56	36.519	0.034	0.17956	-0.4023	0.055922	6.2907e-13
5	---	rs114204311	17	136123277	G	A	0.99987	0.083469	1251.8	0.166	0	0	0	ND	ND	ND
6	---	rs192446092	17	136123600	A	C	0.9999	0.01904	1251.9	0.131	0	0	0	ND	ND	ND
7	---	rs183830736	17	136123679	C	T	0.99982	0.10392	1251.8	0.221	0	0	0	ND	ND	ND
8	---	rs6597618	17	136123740	T	C	0.99414	0.98988	459.62	583.73	208.63	0.01	0.39976	-0.12847	0.040268	0.0014203
9	rs4363269	rs4363269	17	136123840	A	G	1	1	634	517	101	0	0.28714	-0.39307	0.04419	5.8492e-19
10	---	rs186098467	17	136123994	T	C	1	0.0029988	1252	0.003	0	0	0	ND	ND	ND
11	---	rs7025839	17	136124190	G	A	0.99694	0.99335	703.13	474.76	74.108	0.005	0.24879	-0.4253	0.046445	5.3338e-20
12	---	rs72775410	17	136124314	G	A	0.96768	0.3896	1204.1	47.501	0.434	0.002	0.019317	0.079419	0.23598	0.73645
13	---	rs73562371	17	136124326	T	C	0.99955	0.72665	1250.2	1.76	0	0	0.00070288	-1.5209	0.91603	0.096852
14	---	rs9411473	17	136124399	G	A	0.99562	0.98996	702.96	474.93	74.095	0.021	0.24885	-0.42732	0.046516	4.0619e-20
15	---	rs9411370	17	136124432	A	G	0.99538	0.98938	702.97	474.94	74.071	0.015	0.24884	-0.42765	0.04653	3.8938e-20
16	---	rs78755596	17	136124590	T	A	0.99747	0.075064	1248.8	3.171	0	0	0.0012664	ND	ND	ND
17	---	rs115370344	17	136124602	T	G	0.99982	0.017178	1251.8	0.225	0	0	0	ND	ND	ND
18	---	rs12338026	17	136124857	C	G	0.9993	0.58542	1250.4	1.624	0	0	0.00064856	ND	ND	ND
19	---	rs7047076	17	136125329	A	T	0.989	0.97695	702.56	475.81	73.599	0.031	0.24881	-0.43591	0.04683	1.2988e-20
20	---	rs193302481	17	136125592	C	T	1	0.0009992	1252	0.002	0	0	0	ND	ND	ND
21	---	rs9411474	17	136125716	C	G	0.98636	0.97321	702.37	476.16	73.432	0.031	0.24882	-0.43906	0.04693	8.3172e-21
22	---	rs61527302	17	136125994	G	A	0.98778	0.97299	813.69	388.96	49.337	0.007	0.19474	-0.40888	0.050613	6.5466e-16
23	---	rs191151099	17	136126026	T	A	1	1	1252	0	0	0	0	ND	ND	ND
24	---	rs10901251	17	136126129	A	C	0.97626	0.96443	456.22	577.46	218.27	0.044	0.40497	-0.11346	0.040487	0.0050732
25	---	rs148763789	17	136126132	C	T	0.99999	0.0058286	1252	0.012	0	0	0	ND	ND	ND
26	---	rs188658949	17	136126153	G	T	1	0.0019992	1252	0.002	0	0	0	ND	ND	ND
27	---	rs78320709	17	136126394	C	T	0.989	0.49772	1231.6	20.321	0.068	0.002	0.0081697	0.034413	0.28827	0.90498
28	---	rs34266669	17	136126495	C	T	0.98406	0.96903	702.47	476.4	73.112	0.022	0.24866	-0.44461	0.047076	3.5709e-21
29	---	rs151316644	17	136126625	G	A	0.99948	0.039007	1251.3	0.653	0	0	0.00026078	ND	ND	ND
30	---	rs3205136	17	136126631	C	A	0.98673	0.96021	895.8	322.73	33.451	0.018	0.15561	0.41364	0.055894	1.3571e-13
31	---	rs7849280	17	136126636	A	G	0.94202	0.87242	783.14	416.66	52.162	0.033	0.20807	-0.4348	0.052112	7.217e-17
32	---	rs116958345	17	136127122	A	C	0.99974	0.013815	1251.7	0.33	0	0	0.00013179	ND	ND	ND
33	---	rs9411475	17	136127268	T	C	0.95823	0.92214	649.98	507.3	94.676	0.044	0.27823	-0.43752	0.046433	4.4015e-21
34	---	rs118065674	17	136127346	C	T	0.99999	0.0019952	1252	0.012	0	0	0	ND	ND	ND
35	---	rs12216891	17	136127366	C	T	0.97965	0.94627	834.13	376.17	41.661	0.04	0.18351	0.45304	0.053218	1.6973e-17
36	---	chr9:136127382:I	17	136127382	A	AGCCTC	0.99991	0.015581	1251.9	0.115	0	0	0	ND	ND	ND
37	---	rs13291798	17	136127481	A	G	0.99227	0.25953	1242.2	9.761	0	0.002	0.0038982	-0.28934	0.413	0.48356
38	---	rs186319566	17	136127534	C	A	1	1	1252	0	0	0	0	ND	ND	ND
39	---	rs9411476	17	136127601	G	A	0.98081	0.95873	741.55	443.51	66.925	0.017	0.23058	-0.42418	0.047984	9.571e-19
40	---	rs34085694	17	136127605	C	G	0.99157	0.24806	1241.4	10.634	0.003	0.004	0.0042492	-0.35296	0.41426	0.3942
41	---	rs4962113	17	136127641	T	C	0.90386	0.84096	575.07	543.82	133.01	0.099	0.32345	0.10505	0.046389	0.023539
42	---	rs112134200	17	136127689	A	G	0.99849	0.18823	1250.1	1.89	0	0	0.00075479	ND	ND	ND
43	---	rs146579922	17	136127736	C	T	0.99748	0.11506	1248.8	3.154	0	0	0.0012596	ND	ND	ND
44	---	rs115413527	17	136127737	G	A	0.98845	0.26706	1236.9	15.091	0	0	0.0060268	-0.91639	0.4121	0.026168
45	---	rs62574563	17	136127764	C	T	0.96103	0.91922	675.29	484.63	92.006	0.071	0.26704	0.091014	0.046916	0.052387
46	---	rs62574564	17	136127811	G	A	0.96476	0.92562	683.55	482.47	85.92	0.057	0.26132	0.081085	0.047165	0.085579
47	---	rs73660468	17	136127907	G	A	0.99996	0.012682	1251.9	0.054	0	0	0	ND	ND	ND
48	---	rs189931088	17	136127934	C	A	0.99995	0.033943	1251.9	0.058	0	0	0	ND	ND	ND
49	---	rs34039247	17	136127956	A	C	0.99135	0.23459	1241.2	10.828	0.001	0.001	0.0043251	-0.31627	0.40805	0.43829
50	---	rs10901252	17	136128000	G	C	0.99201	0.97662	838.99	369.68	43.306	0.017	0.18223	0.45201	0.052106	4.1441e-18
51	---	rs141318395	17	136128175	G	A	0.99976	0.008487	1251.7	0.299	0	0	0.00011941	ND	ND	ND
52	---	rs62574565	17	136128259	G	A	0.98307	0.9609	706.68	467.13	78.136	0.054	0.24897	0.084741	0.047097	0.071975
53	---	rs58081338	17	136128329	T	C	0.98293	0.96091	705.84	467.9	78.204	0.057	0.24933	0.084609	0.047075	0.072283
54	---	rs62574567	17	136128421	C	G	0.98235	0.95978	706.69	467.16	78.094	0.05	0.24895	0.084946	0.0471	0.071302
55	---	rs60484807	17	136128467	A	G	0.98558	0.97444	425.78	609.68	216.5	0.04	0.41642	0.36313	0.041006	8.3212e-19
56	---	rs7857390	17	136128546	A	G	0.97543	0.94996	111.38	510.4	630.16	0.056	0.29281	-0.014598	0.044559	0.74321
57	---	rs77693339	17	136128558	T	C	0.99453	0.98277	868.78	348.12	35.085	0.016	0.16705	0.51834	0.054001	8.0937e-22
58	---	rs12554580	17	136128603	T	C	0.9858	0.97488	425.82	609.68	216.45	0.043	0.41639	0.36301	0.040996	8.3948e-19
59	---	rs12554336	17	136128663	A	G	0.96931	0.93122	719.48	460.02	72.445	0.053	0.24159	0.092701	0.048318	0.055043
60	---	rs12554595	17	136128664	T	C	0.96954	0.93141	720.32	459.28	72.357	0.049	0.24122	0.092846	0.048342	0.054783
61	---	rs11244051	17	136128731	G	A	0.99583	0.14038	1246.8	5.212	0.003	0	0.0020839	ND	ND	ND
62	---	rs12554339	17	136128737	A	C	0.98236	0.95916	705.65	467.64	78.678	0.038	0.2496	0.084483	0.04707	0.072679
63	---	rs10901253	17	136128772	T	C	0.98353	0.96237	706.03	467.75	78.166	0.047	0.24924	0.08463	0.047043	0.072019
64	---	rs10751502	17	136129079	A	G	0.98545	0.97436	425.26	609.23	217.47	0.042	0.41701	0.36152	0.040954	1.0705e-18
65	---	rs35698328	17	136129112	C	T	0.99154	0.18325	1240.6	11.35	0.009	0.007	0.00454	-0.90886	0.71079	0.20102
66	---	rs10793962	17	136129115	A	T	0.99495	0.98413	868.71	348.23	35.047	0.011	0.16706	0.51799	0.053964	8.0877e-22
67	---	rs11244052	17	136129125	C	A	0.98427	0.96382	706.97	466.93	78.048	0.053	0.24882	0.084778	0.047033	0.071464
68	---	rs11244053	17	136129360	A	G	0.98434	0.96435	706.28	467.59	78.091	0.038	0.24912	0.084566	0.046998	0.071963
69	---	rs4962114	17	136129611	G	A	0.98237	0.95956	709.96	464.38	77.611	0.05	0.24745	0.084934	0.047145	0.07162
70	---	rs4962115	17	136129642	T	G	0.98471	0.9653	706.4	467.49	78.071	0.04	0.24906	0.084551	0.046976	0.071879
71	---	rs8176759	17	136129647	G	A	0.99532	0.98533	868.68	348.31	35.003	0.015	0.16706	0.5176	0.053932	8.2031e-22
72	---	rs1137827	17	136129715	C	T	0.99537	0.98551	868.67	348.32	34.997	0.015	0.16706	0.51758	0.053928	8.1815e-22
73	---	rs4962116	17	136129716	G	A	0.9848	0.96551	706.42	467.47	78.062	0.048	0.24905	0.084559	0.046971	0.071825
74	---	rs188682222	17	136129731	G	A	0.99938	0.028384	1251.2	0.776	0	0.001	0.0003099	ND	ND	ND
75	---	rs8176757	17	136130012	A	C	0.9881	0.97928	426.23	609.66	216.07	0.034	0.41607	0.36198	0.0409	8.7206e-19
76	---	rs117308290	17	136130382	G	A	0.99967	0.23179	1251.6	0.411	0	0	0.00016414	ND	ND	ND
77	---	rs62641783	17	136130653	G	C	0.88545	0.59613	1003.7	236.21	12.038	0.033	0.10395	0.16343	0.086496	0.058827
78	---	rs62641784	17	136130665	G	A	0.91516	0.55494	1077.8	169.01	5.2	0.024	0.07165	0.11004	0.10586	0.29857
79	---	rs62641785	17	136130669	T	C	0.89227	0.71802	888.33	334.16	29.466	0.048	0.15699	0.07006	0.06539	0.28398
80	---	rs62641786	17	136130677	T	C	0.89017	0.66483	963.82	269.33	18.851	0.005	0.12262	0.053855	0.074194	0.46792
81	---	rs184116305	17	136130686	C	G	0.96079	0.4347	1184.9	66.245	0.872	0.001	0.027152	0.27136	0.19231	0.15824
82	---	rs187645401	17	136130689	A	G	0.93208	0.52788	1119.8	128.06	4.125	0.014	0.054437	0.022807	0.11982	0.84904
83	---	rs192412098	17	136130694	C	G	0.94028	0.44951	1150.2	99.31	2.445	0.012	0.041614	0.095911	0.14612	0.51157
84	---	rs62636487	17	136130702	C	T	0.93479	0.62416	1086.1	159	6.873	0.018	0.068988	-0.013765	0.098536	0.8889
85	---	rs141472448	17	136130703	G	A	1	0.0019992	1252	0.002	0	0	0	ND	ND	ND
86	---	rs7469795	17	136130893	T	C	0.97592	0.96017	468.91	593.26	189.78	0.047	0.38852	0.38122	0.041634	5.3636e-20
87	---	rs8176751	17	136131022	C	T	0.99942	0.99823	867.13	349.79	35.078	0.004	0.16771	0.51329	0.053351	6.5243e-22
88	---	chr9:136131056:D	17	136131056	CG	C	0.99846	0.14736	1250.1	1.921	0	0.001	0.00076717	ND	ND	ND
89	---	rs183748371	17	136131065	G	A	1	1	1252	0	0	0	0	ND	ND	ND
90	rs8176749	rs8176749	17	136131188	C	T	1	1	881	338	33	0	0.16134	0.50652	0.054049	7.1554e-21
91	---	rs8176748	17	136131289	C	T	0.99751	0.99449	710.81	462.39	78.797	0.009	0.2476	0.072408	0.046008	0.11553
92	---	rs8176747	17	136131315	C	G	0.99997	0.99989	867.04	349.96	35	0	0.16772	0.51361	0.053316	5.7887e-22
93	---	rs41302905	17	136131316	C	T	0.99999	0.0058286	1252	0.012	0	0	0	ND	ND	ND
94	rs8176746	rs8176746	17	136131322	G	T	1	1	867	350	35	0	0.16773	0.51365	0.053311	5.6972e-22
95	---	rs8176745	17	136131347	G	A	0.99765	0.99502	710.91	462.38	78.694	0.011	0.24751	0.072484	0.046	0.11509
96	---	rs8176744	17	136131350	G	T	0.98229	0.29554	1228.2	23.766	0.027	0	0.0095128	0.3676	0.48119	0.44491
97	---	rs8176743	17	136131415	C	T	0.9978	0.99462	861.32	352.08	38.607	0	0.17144	0.49413	0.0526	5.7768e-21
98	---	rs56116432	17	136131429	C	T	1	1	1252	0	0	0	0	ND	ND	ND
99	---	rs8176742	17	136131437	C	T	0.9935	0.98443	722.51	454.38	75.088	0.021	0.24144	0.077379	0.046555	0.096492
100	---	rs8176741	17	136131461	G	A	0.99775	0.9945	861.25	352.1	38.648	0.001	0.17149	0.49389	0.052593	5.9478e-21
101	---	rs190299333	17	136131469	G	A	0.99712	0.096925	1248.4	3.601	0	0	0.0014381	ND	ND	ND
102	---	rs8176740	17	136131472	A	T	0.99776	0.9953	718.08	456.4	77.508	0.009	0.24418	0.076969	0.04611	0.095066
103	---	rs8176739	17	136131523	G	A	0.9999	0.051417	1251.9	0.121	0	0	0	ND	ND	ND
104	---	rs184446112	17	136131556	G	T	0.99978	0.051382	1251.7	0.272	0	0	0.00010863	ND	ND	ND
105	---	rs55727303	17	136131576	C	T	0.98865	0.6437	1223.4	27.543	1.08	0	0.011862	-0.014439	0.20897	0.94492
106	---	rs55687199	17	136131589	C	T	0.99841	0.46096	1248.5	3.488	0	0.002	0.001393	-0.008709	0.67277	0.98967
107	---	rs7853989	17	136131592	G	C	0.99774	0.99454	861.24	352.09	38.676	0	0.1715	0.49375	0.052586	6.0358e-21
108	---	rs1053878	17	136131651	G	A	0.99077	0.97859	758.22	425.4	68.374	0.01	0.2245	-0.43156	0.047585	1.1981e-19
109	---	rs8176737	17	136131783	C	T	0.99711	0.99388	717.74	456.23	78.024	0.008	0.24452	0.077378	0.046074	0.09307
110	---	rs8176736	17	136131785	T	C	0.99528	0.98945	720.11	454.62	77.263	0.012	0.24327	0.077295	0.046264	0.094773
111	---	rs7873416	17	136131846	T	C	0.99723	0.99538	428.83	606.89	216.26	0.012	0.41511	0.35046	0.040427	4.3643e-18
112	---	rs7855255	17	136131895	C	T	0.99772	0.99454	861.19	352.08	38.729	0.001	0.17154	0.49345	0.052576	6.2617e-21
113	---	rs7873522	17	136131905	T	C	0.99571	0.99233	427.91	606.8	217.29	0.011	0.41589	0.3515	0.040464	3.7329e-18
114	---	rs114820734	17	136132010	T	C	0.99727	0.99542	428.86	606.84	216.29	0.007	0.41511	0.3505	0.040426	4.3153e-18
115	---	rs7873635	17	136132012	T	C	0.9938	0.98724	110.36	513.6	628.03	0.009	0.29326	-0.027255	0.043712	0.53295
116	---	rs113209447	17	136132079	C	T	0.9961	0.99181	719.32	456.25	76.416	0.01	0.24325	0.077027	0.046289	0.096105
117	---	rs112685218	17	136132168	T	C	0.99768	0.99454	861.15	352.08	38.777	0	0.17158	0.49319	0.052566	6.4536e-21
118	---	rs181890403	17	136132205	G	A	0.9998	0.016255	1251.7	0.254	0	0	0.00010144	ND	ND	ND
119	---	rs8176732	17	136132303	A	G	0.99383	0.98643	716.86	457.83	77.302	0.012	0.24458	0.075442	0.046269	0.103
120	---	rs8176731	17	136132350	T	C	0.99741	0.99567	428.81	606.82	216.35	0.015	0.41515	0.35032	0.04042	4.4358e-18
121	---	rs2073823	17	136132516	G	A	0.99758	0.99453	860.98	352.1	38.922	0	0.1717	0.49238	0.052537	7.105e-21
122	---	rs8176730	17	136132525	T	C	0.99761	0.99463	860.99	352.07	38.936	0	0.1717	0.4923	0.052533	7.1643e-21
123	---	rs8176728	17	136132561	G	C	0.99785	0.99567	718.21	456.02	77.765	0.005	0.24423	0.077216	0.046065	0.093692
124	---	rs8176727	17	136132570	G	A	0.99823	0.99639	718.31	456.29	77.398	0.008	0.24405	0.077354	0.046098	0.093342

continue

continued 1

125	---	rs112655244	17	136132608	C	T	0.99795	0.9959	718.35	455.96	77.685	0.007	0.24414	0.077367	0.046072	0.093104
126	---	rs8176725	17	136132617	G	A	0.99526	0.99115	721.82	446.91	83.272	0	0.24499	0.21677	0.045652	2.0507e-06
127	rs2073824	rs2073824	17	136132633	A	G	1	1	327	609	316	0	0.49561	0.22886	0.039285	5.6917e-09
128	---	rs2073825	17	136132707	A	T	0.99883	0.99748	718.55	456.18	77.27	0.001	0.2439	0.077833	0.046096	0.091316
129	---	rs8176722	17	136132754	C	A	0.99867	0.99629	860.62	351.83	39.546	0.001	0.1721	0.4888	0.052404	1.084e-20
130	---	rs8176721	17	136132852	G	A	0.99934	0.062922	1251.2	0.82	0	0.001	0.00032748	ND	ND	ND
131	---	rs8176720	17	136132873	T	C	0.99964	0.9993	428.06	606.21	217.73	0	0.416	0.34913	0.04032	4.7599e-18
132	---	chr9:136132908:I	17	136132908	T	TC	0.99875	0.99767	387.31	595.14	269.54	0.002	0.45297	-0.087771	0.03922	0.025227
133	---	rs75179845	17	136132954	T	C	0.99743	0.99301	862.35	350.51	39.138	0.003	0.17124	0.488	0.052745	2.2012e-20
134	---	rs8176718	17	136132957	C	T	0.99986	0.99962	718.94	456.01	77.051	0	0.24365	0.078603	0.046101	0.088188
135	---	rs8176717	17	136133034	G	T	0.99933	0.99837	719.63	455.38	76.989	0	0.24335	0.078788	0.046125	0.087613
136	---	rs8176716	17	136133065	G	T	0.99863	0.15653	1250.3	1.712	0	0	0.00068371	ND	ND	ND
137	---	rs8176715	17	136133148	T	C	0.98802	0.97381	83.13	476.46	692.39	0.02	0.25668	-0.035566	0.045948	0.4389
138	---	rs191976170	17	136133149	G	A	0.99921	0.24201	1251	0.989	0	0	0.00039497	ND	ND	ND
139	---	rs8176714	17	136133178	G	A	0.99356	0.98601	648.13	505.84	98.016	0.01	0.2803	0.069039	0.044583	0.12149
140	---	rs115166192	17	136133218	C	T	0.99965	0.0086304	1251.6	0.441	0	0	0.00017612	ND	ND	ND
141	---	rs182985414	17	136133346	A	C	1	1	1252	0	0	0	0	ND	ND	ND
142	---	chr9:136133380:D	17	136133380	CGGG	C	0.99867	0.99649	76.608	455.3	720.09	0.004	0.24302	-0.07819	0.046199	0.090558
143	---	rs45610939	17	136133396	A	G	0.9792	0.8157	1146.1	105.73	0.204	0.003	0.042387	-0.055135	0.10946	0.61446
144	rs512770	rs512770	17	136133506	A	G	1	1	77	456	719	0	0.24361	-0.078689	0.046104	0.087862
145	---	rs4962040	17	136133531	A	G	0.99764	0.99606	629.73	513.26	109.01	0.008	0.29204	0.028954	0.043672	0.50734
146	---	chr9:136133582:D	17	136133582	AG	A	0.99258	0.98658	91.997	483.41	676.59	0.002	0.26654	-0.12006	0.045053	0.0077009
147	---	rs641959	17	136133699	C	A	0.99758	0.99575	89.25	468.68	694.07	0.001	0.25846	-0.092724	0.045021	0.039439
148	---	rs641943	17	136133714	G	A	0.99778	0.99624	89.165	468.59	694.24	0.001	0.25836	-0.09265	0.045023	0.039609
149	---	rs514708	17	136133743	T	C	0.99778	0.99625	89.165	468.59	694.24	0	0.25836	-0.092649	0.045023	0.039611
150	---	rs517414	17	136134034	A	G	0.9996	0.99919	80.042	459.41	712.54	0	0.2474	-0.086682	0.045827	0.058558
151	---	rs9411372	17	136134068	G	A	0.97122	0.94646	730.07	444.16	77.763	0.012	0.23949	-0.4368	0.047436	3.3133e-20
152	---	rs138164693	17	136134118	G	A	1	1	1252	0	0	0	0	ND	ND	ND
153	---	rs638756	17	136134472	C	A	0.99787	0.99641	89.116	468.64	694.24	0	0.25834	-0.092673	0.045026	0.039568
154	---	rs191050088	17	136134650	C	A	0.99671	0.14604	1247.9	4.122	0	0	0.0016462	ND	ND	ND
155	---	rs183228393	17	136134835	G	A	1	0.0033309	1252	0.006	0	0	0	ND	ND	ND
156	---	rs626792	17	136134861	C	A	0.93522	0.87801	103.9	510.59	637.49	0.026	0.2869	-0.051962	0.046684	0.26568
157	---	rs626035	17	136134994	G	T	0.97273	0.94743	99.363	483.5	669.14	0.006	0.27245	-0.1037	0.045359	0.022244
158	---	rs547495	17	136134995	C	T	0.97273	0.94743	99.363	483.49	669.14	0.006	0.27245	-0.1037	0.045359	0.022241
159	---	rs547643	17	136135047	T	C	1	1	80.001	459	713	0	0.2472	-0.085481	0.045802	0.061997
160	---	rs625593	17	136135096	A	G	0.99998	0.99995	80.002	459.02	712.98	0	0.24721	-0.085472	0.045802	0.062026
161	---	rs8176707	17	136135108	G	T	0.9361	0.7539	1021.6	214.61	15.814	0.015	0.098338	-0.045788	0.075941	0.54655
162	---	chr9:136135137:D	17	136135137	GCTTCTGTCCACAC	G	0.98818	0.97538	85.311	466.49	700.19	0.007	0.25444	-0.077415	0.045792	0.09092
163	---	rs138374634	17	136135194	G	A	0.99916	0.84615	1246.3	5.664	0	0	0.002262	-0.049472	0.43827	0.91013
164	---	rs549331	17	136135195	G	C	0.99799	0.99664	89.053	468.71	694.24	0	0.25831	-0.092707	0.045029	0.039509
165	---	rs549443	17	136135237	A	G	0.89151	0.7955	120.08	528.79	603.05	0.085	0.30711	-0.13429	0.048546	0.0056698
166	---	rs549446	17	136135238	T	C	0.88939	0.79851	130.45	531.66	589.83	0.055	0.31653	-0.13087	0.047747	0.0061278
167	---	rs149279023	17	136135321	G	A	1	1	1252	0	0	0	0	ND	ND	ND
168	---	rs624601	17	136135365	A	G	0.99799	0.99664	89.053	468.71	694.24	0	0.25831	-0.092707	0.045029	0.039509
169	---	rs551322	17	136135444	G	A	1	1	80	459	713	0	0.2472	-0.085481	0.045802	0.061997
170	---	rs192192638	17	136135445	C	T	0.96586	0.37961	1202.7	49.017	0.258	0.008	0.019782	1.0383	0.26064	6.7827e-05
171	---	rs188088103	17	136135472	C	T	0.99846	0.029421	1250.1	1.927	0	0.002	0.00076957	ND	ND	ND
172	---	rs613423	17	136135478	A	G	1	1	80	459	713	0	0.2472	-0.085481	0.045802	0.061997
173	---	rs8176704	17	136135552	G	A	0.99874	0.1339	1250.4	1.574	0	0	0.00062859	ND	ND	ND
174	---	chr9:136135625:D	17	136135625	CTG	C	1	1	1252	0	0	0	0	ND	ND	ND
175	rs574347	rs574347	17	136135659	C	T	1	1	80	459	713	0	0.2472	-0.085481	0.045802	0.061997
176	---	rs575259	17	136135752	G	A	0.99798	0.99664	89.052	468.71	694.24	0.001	0.25831	-0.092713	0.045029	0.039497
177	---	rs8176703	17	136135863	G	T	0.99894	0.21781	1250.7	1.35	0	0	0.00053914	ND	ND	ND
178	---	rs8176702	17	136136146	G	A	0.9983	0.99712	630.2	512.79	109.01	0.001	0.29186	0.029119	0.043645	0.50466
179	---	rs579483	17	136136196	A	T	0.99999	0.99998	80	459.01	712.99	0	0.24721	-0.085506	0.045803	0.061924
180	---	rs579622	17	136136242	A	G	0.99999	0.99997	80	459.01	712.99	0	0.24721	-0.085508	0.045803	0.061918
181	---	rs140166545	17	136136367	C	T	0.99989	0.058102	1251.9	0.137	0	0	0	ND	ND	ND
182	---	rs493211	17	136136516	A	G	0.99977	0.9994	79.938	459.06	713	0.002	0.24718	-0.085489	0.045825	0.062106
183	---	rs8176698	17	136136593	T	C	0.9993	0.065935	1251.1	0.874	0	0	0.00034904	ND	ND	ND
184	---	rs688976	17	136136770	A	C	0.99996	0.99989	80.015	459.02	712.96	0	0.24722	-0.08554	0.045802	0.06182
185	---	rs8176696	17	136136773	C	T	0.9993	0.065935	1251.1	0.874	0	0	0.00034904	ND	ND	ND
186	---	rs2073826	17	136136963	G	T	0.99779	0.99642	629.42	513.48	109.1	0.002	0.2922	0.029354	0.043658	0.50135
187	---	rs112565602	17	136137050	G	A	0.9982	0.21075	1249.7	2.259	0	0	0.00090216	ND	ND	ND
188	---	rs687621	17	136137065	A	G	0.99796	0.99735	378.17	594.14	279.69	0.002	0.46067	-0.085673	0.03911	0.028483
189	---	rs184686147	17	136137088	G	A	0.99996	0.020119	1252	0.044	0	0	0	ND	ND	ND
190	---	rs687289	17	136137106	G	A	0.99985	0.99973	393.1	591.97	266.92	0	0.44961	-0.093509	0.039153	0.016926
191	---	rs2073827	17	136137133	G	C	1	1	609	527	116	0	0.30312	0.037734	0.043232	0.38275
192	rs2073828	rs2073828	17	136137140	G	A	1	1	609	527	116	0	0.30312	0.037734	0.043232	0.38275
193	---	rs189048957	17	136137228	G	A	0.99997	0.019153	1252	0.036	0	0	0	ND	ND	ND
194	---	rs150021186	17	136137293	G	A	1	1	1252	0	0	0	0	ND	ND	ND
195	---	rs77513793	17	136137296	T	C	0.99874	0.38576	1249.6	2.428	0	0	0.00096965	ND	ND	ND
196	---	chr9:136137375:I	17	136137375	A	AT	1	1	1252	0	0	0	0	ND	ND	ND
197	---	rs55876802	17	136137547	C	A	1	1	1252	0	0	0	0	ND	ND	ND
198	---	rs143174772	17	136137600	C	T	1	1	1252	0	0	0	0	ND	ND	ND
199	---	rs151155628	17	136137641	C	T	1	1	1252	0	0	0	0	ND	ND	ND
200	rs8176694	rs8176694	17	136137646	T	C	1	1	963	273	16	0	0.12181	0.086651	0.061457	0.15855
201	---	rs8176693	17	136137657	C	T	0.99885	0.99686	867.98	346.46	37.558	0.001	0.16836	0.48719	0.052926	3.409e-20
202	---	rs673578	17	136137857	G	T	0.99739	0.99552	88.963	468.74	694.3	0.001	0.25825	-0.092876	0.045057	0.039275
203	---	rs8176692	17	136137937	T	C	0.99811	0.9461	1225.3	25.718	0.996	0.001	0.011066	0.17829	0.18916	0.34594
204	---	rs145090216	17	136138074	A	G	1	1	1252	0	0	0	0	ND	ND	ND
205	---	rs672316	17	136138125	G	T	0.99727	0.99545	88.942	468.68	694.38	0.002	0.25821	-0.09287	0.04506	0.039303
206	---	rs8176691	17	136138229	C	T	0.99753	0.99578	629.41	513.17	109.41	0.002	0.29233	0.029205	0.043636	0.50332
207	---	rs8176690	17	136138317	A	G	0.99914	0.99846	608.21	527.37	116.42	0.002	0.3036	0.037849	0.043227	0.38126
208	---	rs189530394	17	136138484	A	C	0.99787	0.18975	1249.3	2.673	0	0	0.0010675	ND	ND	ND
209	---	chr9:136138557:I	17	136138557	A	AT	0.97855	0.32172	1219	32.845	0.161	0.019	0.013246	-0.37308	0.30051	0.21442
210	---	rs8176688	17	136138583	C	T	0.99895	0.22039	1250.7	1.332	0	0	0.00053195	ND	ND	ND
211	---	chr9:136138765:D	17	136138765	GCGCCCACCACTA	G	0.99123	0.93635	1118.2	132.71	1.127	0.007	0.053901	-0.35689	0.092517	0.00011454
212	---	rs8176685	17	136138776	T	C	0.96729	0.93963	624.46	519.27	108.25	0.02	0.29384	0.047067	0.045054	0.29618
213	---	rs186003835	17	136138804	G	A	0.99905	0.092474	1250.8	1.188	0.002	0	0.00047604	ND	ND	ND
214	---	rs8176684	17	136138874	G	A	0.99907	0.058687	1250.8	1.165	0	0.001	0.00046526	ND	ND	ND
215	---	rs147279040	17	136138978	C	T	1	1	1252	0	0	0	0	ND	ND	ND
216	---	rs140783133	17	136139110	A	C	0.99943	0.0989	1251.3	0.714	0	0	0.00028514	ND	ND	ND
217	---	rs147622835	17	136139238	G	A	0.98897	0.66567	1221	29.835	1.121	0.003	0.01281	-0.019081	0.19989	0.92395
218	rs657152	rs657152	17	136139265	C	A	1	1	400	601	251	0	0.4405	-0.11187	0.039583	0.0047109
219	---	rs8176682	17	136139297	C	T	0.99739	0.99546	616.32	520.13	115.55	0.001	0.30001	0.046254	0.043285	0.28525
220	---	rs139568229	17	136139383	G	T	1	1	1252	0	0	0	0	ND	ND	ND
221	---	rs149756392	17	136139395	G	C	1	1	1252	0	0	0	0	ND	ND	ND
222	---	rs145595975	17	136139460	G	A	0.99811	0.94713	1225.3	25.67	0.998	0.001	0.011049	0.17856	0.18914	0.34513
223	---	rs148824570	17	136139589	C	A	1	1	1252	0	0	0	0	ND	ND	ND
224	---	rs474279	17	136139617	T	C	0.99886	0.99784	81.091	458.96	711.95	0	0.24806	-0.086789	0.045736	0.05775
225	---	rs146458069	17	136139672	C	T	1	1	1252	0	0	0	0	ND	ND	ND
226	---	rs8176765	17	136139694	A	C	0.9988	0.36712	1249.9	2.121	0	0	0.00084704	ND	ND	ND
227	---	rs191325486	17	136139702	G	A	0.99482	0.30152	1244.5	7.535	0.001	0	0.00301	-0.32817	0.55801	0.55646
228	---	rs8176681	17	136139754	T	C	0.99692	0.99523	615.6	520.78	115.62	0.001	0.30033	0.046591	0.043282	0.28172
229	---	rs144649415	17	136139775	G	T	0.99323	0.9169	1164.4	83.111	4.491	0.009	0.036779	-0.025694	0.10571	0.80796
230	---	rs141515001	17	136139833	T	C	1	1	1252	0	0	0	0	ND	ND	ND
231	---	chr9:136139907:D	17	136139907	GAAACTGCC	G	0.99662	0.9925	649.03	505.2	97.761	0.004	0.27984	-0.46331	0.044479	2.0858e-25
232	---	rs186048848	17	136140035	G	A	0.9977	0.11318	1249.1	2.875	0	0	0.0011482	ND	ND	ND
233	---	rs1633513	17	136140462	C	T	0.99669	0.99412	90.333	468.89	692.78	0	0.25941	-0.093092	0.04497	0.038442
234	---	rs149567216	17	136140469	C	T	1	1	1252	0	0	0	0	ND	ND	ND
235	---	rs1633514	17	136140513	T	C	0.99827	0.99689	81.247	458.84	711.91	0	0.24814	-0.085837	0.045753	0.06064
236	---	rs143159728	17	136140613	T	G	1	1	1252	0	0	0	0	ND	ND	ND
237	---	rs115325682	17	136140759	G	A	0.99914	0.4883	1250.1	1.943	0	0	0.00077596	ND	ND	ND
238	---	rs1752339	17	136141135	C	T	0.99063	0.98026	81.109	449.2	721.69	0.006	0.24418	-0.08876	0.046057	0.053954
239	---	rs142141716	17	136141226	G	C	1	1	1252	0	0	0	0	ND	ND	ND
240	---	rs13299342	17	136141504	G	A	0.99527	0.98648	872.75	342.77	36.481	0.003	0.16603	0.47865	0.053521	3.7761e-19
241	---	rs2519093	17	136141870	C	T	0.99806	0.99625	647.5	505.95	98.545	0.004	0.28077	-0.46247	0.044343	1.8217e-25
242	---	rs144140881	17	136141877	G	T	1	0.0059976	1252	0.006	0	0	0	ND	ND	ND
243	---	rs113423093	17	136142000	G	C	0.96439	0.93239	103.39	487.25	661.33	0.03	0.27718	-0.090103	0.04523	0.046358
244	---	rs8176672	17	136142185	C	T	0.99786	0.99415	874.05	341.66	36.29	0.001	0.16543	0.48038	0.053372	2.245e-19
245	---	rs514659	17	136142203	A	C	0.99836	0.99762	397.73	589.42	264.85	0	0.44693	-0.10224	0.03916	0.0090332
246	---	rs644234	17	136142217	T	G	0.99837	0.99763	397.73	589.42	264.85	0	0.44693	-0.10225	0.03916	0.009027
247	---	rs182705402	17	136142293	C	T	1	1	1252	0	0	0	0	ND	ND	ND
248	---	rs140796254	17	136142304	A	T	1	1	1252	0	0	0	0	ND	ND	ND
249	---	rs8176671	17	136142313	A	C	0.9968	0.99114	874.8	341.48	35.716	0.002	0.1649	0.4812	0.053612	2.8219e-19
250	---	rs143309559	17	136142339	C	T	1	1	1252	0	0	0	0	ND	ND	ND
251	---	rs643434	17	136142355	G	A	0.99816	0.99722	397.68	589.26	265.06	0.001	0.44704	-0.10231	0.039158	0.0089809

continued

continued 2

252	---	rs8176669	17	136142463	A	C	0.99711	0.99461	707.9	460.87	83.226	0.005	0.25053	0.09383	0.045625	0.039729
253	---	rs148987354	17	136142465	G	A	0.9999	0.020262	1251.9	0.128	0	0	0	ND	ND	ND
254	---	chr9:136142521:I	17	136142521	C	CT	0.99487	0.89609	1204.1	47.878	0	0.004	0.019121	-0.50178	0.15256	0.0010056
255	---	chr9:136142540:I	17	136142540	T	TTG	1	1	1252	0	0	0	0	ND	ND	ND
256	---	rs143746661	17	136142711	A	G	1	1	1252	0	0	0	0	ND	ND	ND
257	---	rs187821258	17	136142968	T	C	0.99742	0.11314	1248.8	3.226	0	0	0.0012883	ND	ND	ND
258	---	rs543040	17	136143000	A	T	0.99582	0.9934	396.75	589.54	265.71	0.001	0.44767	-0.10259	0.039183	0.0088412
259	---	rs146903770	17	136143055	A	G	0.99111	0.28893	1240.3	11.683	0	0	0.0046657	-1.2328	0.50413	0.014472
260	---	rs613534	17	136143120	A	G	0.99583	0.99385	399.27	589.45	263.29	0.001	0.4457	-0.10427	0.039263	0.007913
261	---	rs543968	17	136143121	T	C	0.99437	0.9912	400.49	588.95	262.56	0.002	0.44492	-0.10659	0.039365	0.0067754
262	---	rs139081859	17	136143192	G	T	0.99825	0.077245	1249.8	2.196	0	0.001	0.000877	ND	ND	ND
263	---	rs544873	17	136143212	G	A	0.99502	0.9917	402.45	586.52	263.02	0	0.44432	-0.10442	0.039194	0.0077153
264	---	rs142956930	17	136143330	A	G	0.99889	0.098024	1250.6	1.391	0	0	0.00055551	ND	ND	ND
265	---	rs545971	17	136143372	C	T	0.99838	0.99763	397.73	589.42	264.85	0	0.44693	-0.10225	0.039161	0.0090282
266	---	rs138194969	17	136143391	G	A	1	1	1252	0	0	0	0	ND	ND	ND
267	---	rs612169	17	136143442	A	G	0.99839	0.99764	397.73	589.42	264.85	0	0.44693	-0.10225	0.03916	0.0090285
268	---	rs144566139	17	136143919	G	A	1	1	1252	0	0	0	0	ND	ND	ND
269	---	chr9:136144000:D	17	136144000	AT	A	0.98756	0.54188	1223.5	28.432	0.053	0.001	0.011397	0.41596	0.25184	0.098597
270	---	rs8176668	17	136144059	A	T	0.99815	0.9973	611.89	523.75	116.36	0	0.3021	0.039923	0.043211	0.35553
271	---	rs574311	17	136144110	G	A	0.99799	0.99662	83.583	461.25	707.17	0	0.25096	-0.093923	0.045539	0.039165
272	---	rs187551277	17	136144172	G	A	0.99825	0.077245	1249.8	2.196	0	0.001	0.000877	ND	ND	ND
273	---	rs597988	17	136144284	T	A	0.97199	0.95467	459.93	572.81	219.24	0.019	0.40388	-0.092999	0.040572	0.021895
274	---	rs597974	17	136144297	A	G	0.96877	0.94921	462.62	571.57	217.79	0.018	0.40222	-0.093592	0.040683	0.021418
275	---	rs140114497	17	136144308	T	C	0.96718	0.9468	463.77	571.11	217.11	0.017	0.40149	-0.096203	0.040787	0.01834
276	---	rs576125	17	136144309	G	A	0.96718	0.94681	463.77	571.1	217.12	0.017	0.4015	-0.0962	0.040786	0.018343
277	---	rs8176663	17	136144427	T	C	0.99836	0.99757	397.61	589.5	264.89	0	0.447	-0.10235	0.039164	0.0089644
278	---	rs8176662	17	136144454	A	G	0.99669	0.9908	874.92	341.45	35.619	0.004	0.16481	0.48185	0.053637	2.6219e-19
279	---	rs149899707	17	136144521	C	T	1	1	1252	0	0	0	0	ND	ND	ND
280	---	rs488775	17	136144534	G	A	0.9979	0.99644	83.591	461.14	707.27	0.001	0.25093	-0.094063	0.045543	0.038888
281	---	rs66697526	17	136144593	G	T	0.99595	0.98605	958.35	272.93	20.717	0.007	0.12554	0.061971	0.060295	0.30405
282	---	rs7036642	17	136144626	G	A	0.9981	0.99718	611.96	523.69	116.36	0	0.30208	0.039927	0.043211	0.35549
283	---	rs8176661	17	136144640	A	G	0.99996	0.0086711	1252	0.045	0	0	0	ND	ND	ND
284	---	rs596141	17	136144689	T	G	0.99816	0.99701	79.694	461.37	710.94	0	0.24791	-0.083621	0.045896	0.068458
285	---	rs146907040	17	136144771	A	G	1	0.0039984	1252	0.004	0	0	0	ND	ND	ND
286	---	rs491626	17	136144873	C	T	0.99109	0.98649	411.75	583.83	256.42	0.001	0.43797	-0.11815	0.039366	0.0026882
287	---	rs492488	17	136144960	G	A	0.99656	0.99462	391.81	591.2	269	0	0.45095	-0.094233	0.039206	0.016237
288	---	rs493246	17	136144994	G	A	0.98929	0.982	384.57	593.12	274.3	0.01	0.45596	-0.097317	0.039427	0.013576
289	---	rs193238931	17	136145029	C	T	0.99979	0.10628	1251.7	0.261	0	0	0.00010423	ND	ND	ND
290	---	rs494242	17	136145118	C	T	0.99587	0.99328	391.13	591.58	269.29	0.001	0.45134	-0.094149	0.039245	0.016439
291	---	rs495203	17	136145240	C	T	0.99587	0.99328	391.13	591.58	269.29	0.001	0.45134	-0.094146	0.039244	0.016441
292	---	chr9:136145403:D	17	136145403	AC	A	0.89421	0.82553	562.28	557.59	132.08	0.05	0.32819	-0.27512	0.047402	6.4719e-09
293	---	chr9:136145414:I	17	136145414	C	CA	0.90476	0.60485	1022.7	217.57	11.653	0.032	0.096197	0.53079	0.090195	3.9815e-09
294	---	chr9:136145418:D	17	136145418	AC	A	0.88378	0.80486	584.03	533.39	134.53	0.044	0.32048	-0.22009	0.047346	3.3413e-06
295	---	chr9:136145424:D	17	136145424	AC	A	0.96664	0.92861	684.01	483.64	84.323	0.03	0.2605	-0.46783	0.04682	1.6535e-23
296	---	rs9411378	17	136145425	C	A	0.96466	0.92381	684.98	482.46	84.528	0.038	0.2602	-0.46476	0.046865	3.5102e-23
297	---	rs582118	17	136145471	A	G	0.99785	0.9965	392.39	591.52	268.09	0	0.45036	-0.093554	0.039192	0.016983
298	---	rs582094	17	136145484	A	T	0.99785	0.9965	392.39	591.52	268.09	0	0.45036	-0.093554	0.039192	0.016983
299	---	rs189022067	17	136145717	A	G	0.99986	0.035747	1251.8	0.179	0	0	0	ND	ND	ND
300	---	chr9:136145907:I	17	136145907	G	GA	0.99609	0.99346	395.16	590.05	266.79	0.001	0.44874	-0.092758	0.03919	0.017939
301	---	rs2769071	17	136145974	A	G	0.99601	0.99357	392.38	591.18	268.44	0.004	0.4505	-0.09383	0.039183	0.016635
302	---	rs147027134	17	136145979	A	G	1	1	1252	0	0	0	0	ND	ND	ND
303	---	rs677355	17	136146046	G	A	0.99691	0.99484	394.01	590.7	267.29	0.003	0.44939	-0.093172	0.039196	0.017449
304	---	chr9:136146061:I	17	136146061	T	TA	0.99429	0.99034	398.01	585.8	268.18	0.009	0.44815	-0.096895	0.039108	0.013225
305	---	chr9:136146068:I	17	136146068	A	AT	0.99726	0.99542	393.77	590.73	267.49	0.005	0.44957	-0.093428	0.039176	0.017087
306	---	rs676996	17	136146077	T	G	0.99628	0.99354	399.35	585.54	267.11	0.005	0.44719	-0.096516	0.039087	0.01354
307	---	rs676457	17	136146227	A	T	0.99785	0.99652	393.66	590.84	267.5	0.002	0.44962	-0.093485	0.039164	0.016985
308	---	rs79343853	17	136146310	T	C	0.99653	0.99042	867.38	349.01	35.609	0.006	0.16782	0.49488	0.053462	2.1116e-20
309	---	rs527210	17	136146431	C	T	0.99668	0.99439	399.33	585.7	266.97	0.001	0.44714	-0.095837	0.039105	0.014254
310	---	chr9:136146448:D	17	136146448	TAAGAC	T	0.98769	0.98377	406.78	582.92	262.29	0.011	0.44229	-0.10486	0.039277	0.007592
311	---	rs675201	17	136146466	A	G	0.99409	0.98811	82.173	466.32	703.51	0.005	0.25186	-0.095002	0.045949	0.038682
312	---	rs182739868	17	136146499	A	G	0.91465	0.6591	1001.1	240.33	10.487	0.045	0.10436	-0.0047794	0.082899	0.95403
313	---	rs550057	17	136146597	C	T	0.99831	0.9969	646.08	506.91	99.016	0.001	0.28153	-0.46233	0.044317	1.7678e-25
314	---	rs674302	17	136146664	T	A	0.99861	0.99795	393.03	591.17	267.8	0	0.44999	-0.093631	0.039154	0.016786
315	---	rs551100	17	136146740	T	C	0.99788	0.99613	79.842	461.53	710.62	0.001	0.24809	-0.083565	0.045895	0.068641
316	---	rs187072196	17	136146756	C	A	0.99994	0.016025	1251.9	0.074	0	0	0	ND	ND	ND
317	---	rs191709829	17	136146850	C	T	0.9983	0.71163	1245.5	6.497	0	0	0.0025946	0.56353	0.44106	0.20137
318	---	rs663054	17	136146920	T	G	0.99796	0.99619	79.871	461.51	710.61	0.002	0.24811	-0.083391	0.045889	0.069184
319	---	rs7046674	17	136147012	C	T	0.99716	0.99473	612.89	523.29	115.82	0.004	0.30149	0.040452	0.043295	0.35012
320	---	rs554833	17	136147160	C	T	0.99889	0.99822	393.07	591.1	267.83	0.001	0.44998	-0.093501	0.039145	0.016913
321	---	rs8176649	17	136147295	G	A	0.99727	0.99545	634.51	508.13	109.36	0	0.29028	0.031708	0.043596	0.46704
322	---	rs141837362	17	136147453	T	C	0.99977	0.29192	1251.7	0.292	0	0	0.00011661	ND	ND	ND
323	---	rs144458168	17	136147539	T	C	1	1	1252	0	0	0	0	ND	ND	ND
324	---	rs660340	17	136147553	A	G	0.99831	0.99728	270.49	597.73	383.79	0	0.45475	-0.078192	0.039294	0.046601
325	---	chr9:136147630:I	17	136147630	G	GA	0.99992	0.081283	1251.9	0.1	0	0	0	ND	ND	ND
326	---	rs148402540	17	136147666	A	G	1	1	1252	0	0	0	0	ND	ND	ND
327	---	rs581107	17	136147702	C	T	0.99615	0.9931	265.48	596.95	389.57	0.002	0.45044	-0.076468	0.039396	0.052255
328	---	rs659104	17	136147823	T	G	0.99848	0.99754	270.5	597.71	383.79	0	0.45475	-0.078122	0.039289	0.04677
329	---	rs150947224	17	136147988	T	C	1	0.0009996	1252	0.001	0	0	0	ND	ND	ND
330	---	rs647800	17	136148000	A	G	0.99857	0.9977	270.5	597.71	383.79	0.002	0.45475	-0.07808	0.039287	0.046875
331	---	rs473533	17	136148035	T	C	0.99828	0.99718	270.49	597.99	383.52	0.004	0.45486	-0.078021	0.039299	0.047109
332	---	rs475419	17	136148231	C	T	0.99867	0.99785	270.55	597.66	383.79	0	0.45478	-0.078008	0.039281	0.047046
333	---	rs476410	17	136148368	G	C	0.99854	0.99765	270.52	597.98	383.5	0.001	0.45488	-0.077914	0.039289	0.047356
334	---	rs645982	17	136148409	A	G	0.99883	0.99816	270.5	597.69	383.8	0	0.45475	-0.077955	0.039278	0.04718
335	rs500498	rs500498	17	136148647	T	C	1	1	271	598	383	0	0.45527	-0.078134	0.039252	0.046527
336	---	rs500499	17	136148648	G	C	0.99776	0.99613	276.7	607.35	367.95	0.004	0.46356	-0.093388	0.039504	0.018079
337	---	rs149135480	17	136148703	T	A	0.99985	0.018738	1251.8	0.193	0	0	0	ND	ND	ND
338	---	rs143292918	17	136148765	G	A	1	0.0009996	1252	0.001	0	0	0	ND	ND	ND
339	---	chr9:136149095:D	17	136149095	CA	C	0.98795	0.97957	417.68	594.87	239.43	0.016	0.42881	-0.059622	0.039999	0.13607
340	---	rs116552240	17	136149098	T	A	0.98795	0.97959	417.68	594.87	239.43	0.014	0.42881	-0.059621	0.039999	0.13607
341	---	rs8176644	17	136149150	C	T	0.99735	0.99242	867.67	349.02	35.312	0.003	0.16759	0.49636	0.053482	1.6808e-20
342	---	rs188675313	17	136149152	T	C	0.99998	0.005944	1252	0.021	0	0	0	ND	ND	ND
343	rs505922	rs505922	17	136149229	T	C	1	1	393	590	269	0	0.45048	-0.093823	0.039078	0.016355
344	---	rs507666	17	136149399	G	A	0.9981	0.99627	647.06	505.68	99.258	0.002	0.28123	-0.46225	0.044289	1.6813e-25
345	---	rs186959044	17	136149400	G	A	0.9973	0.31509	1247.8	4.211	0	0	0.0016817	-0.11501	1.092	0.91612
346	---	rs529565	17	136149500	T	C	0.99996	0.99991	392.98	591.02	268	0	0.45009	-0.093149	0.039116	0.01725
347	---	rs630510	17	136149581	A	G	0.99927	0.99863	170.56	545.98	535.46	0.001	0.35427	-0.088794	0.040697	0.029121
348	---	chr9:136149709:D	17	136149709	AC	A	0.99594	0.97136	1110.5	140.35	1.126	0.002	0.056949	-0.34739	0.089164	9.7741e-05
349	rs630014	rs630014	17	136149722	A	G	1	1	171	546	535	0	0.35463	-0.08832	0.040659	0.02984
350	---	rs532436	17	136149830	G	A	0.99808	0.99626	647.09	505.65	99.259	0.004	0.28122	-0.46229	0.044289	1.6631e-25
351	---	rs8176639	17	136150317	C	G	0.99965	0.08622	1251.6	0.434	0	0	0.00017332	ND	ND	ND
352	---	rs8176760	17	136150379	G	A	0.9827	0.22758	1229.2	22.622	0.129	0.006	0.0091374	-0.020973	0.40913	0.95912
353	---	rs537895	17	136150403	A	G	0.97813	0.95692	97.54	473.04	681.38	0.036	0.26683	-0.032909	0.045139	0.46597
354	---	rs616154	17	136150466	C	T	0.98953	0.98563	430.7	595.98	225.31	0.004	0.41798	0.21997	0.04023	4.5551e-08
355	---	rs559723	17	136150484	A	G	0.9905	0.98559	433.68	593.65	224.67	0.009	0.41653	0.21595	0.040348	8.687e-08
356	---	rs144289739	17	136150522	C	G	0.99782	0.50843	1248.3	3.724	0	0	0.0014872	0.98139	0.96362	0.21812
357	---	rs8176638	17	136150556	C	T	0.9655	0.54407	1177.3	73.253	1.443	0.024	0.030408	0.6064	0.16252	0.00019051
358	---	rs140079020	17	136150809	C	T	0.99889	0.078057	1250.6	1.388	0	0.001	0.00055431	ND	ND	ND
359	---	chr9:136150912:I	17	136150912	C	CG	0.99999	0.0096102	1252	0.013	0	0	0	ND	ND	ND
360	---	rs142995738	17	136151065	C	G	0.99533	0.69746	1237.1	14.878	0.002	0.001	0.0059433	-0.23342	0.28963	0.42028
361	---	rs190049657	17	136151107	C	T	0.99998	0.004605	1252	0.026	0	0	0	ND	ND	ND
362	---	rs566183	17	136151198	A	G	0.99981	0.9996	80.003	460.79	711.21	0	0.24792	-0.081051	0.045805	0.076815
363	rs568203	rs568203	17	136151445	A	C	1	1	80	461	711	0	0.248	-0.081362	0.045801	0.075662
364	---	rs600038	17	136151806	T	C	0.99859	0.99702	647.51	505.09	99.397	0.005	0.28111	-0.46281	0.044268	1.3914e-25
365	---	rs8176635	17	136152009	G	A	0.99781	0.99572	509.3	574.27	168.43	0	0.36387	0.30993	0.041281	6.0158e-14
366	---	rs8176634	17	136152070	G	C	0.9989	0.99671	822.29	369.72	59.983	0.002	0.19556	0.017997	0.048815	0.71237
367	---	rs150185195	17	136152286	C	T	0.98223	0.82631	1155.9	96.035	0.055	0.005	0.038397	-0.085406	0.1143	0.45494
368	---	rs146783421	17	136152336	A	C	1	0.003398	1252	0.005	0	0	0	ND	ND	ND
369	---	rs182406156	17	136152347	C	T	0.99998	0.0061435	1252	0.026	0	0	0	ND	ND	ND
370	---	rs8176633	17	136152354	C	T	0.9965	0.99222	800.55	385.48	65.975	0	0.20664	0.029077	0.047933	0.5441
371	---	rs145690885	17	136152368	G	A	0.97486	0.40551	1210.5	41.39	0.098	0.004	0.016608	0.24507	0.22969	0.28598
372	---	rs8176632	17	136152547	C	T	0.99758	0.99186	867.35	348.72	35.923	0.005	0.16796	0.49687	0.05342	1.388e-20
373	---	rs8176631	17	136152594	C	T	0.99974	0.041108	1251.7	0.321	0	0	0.00012819	ND	ND	ND
374	---	rs8176630	17	136152722	C	T	0.99683	0.98398	1023.4	219.58	9.029	0.003	0.094904	0.053482	0.069485	0.44148
375	---	rs188389911	17	136153355	G	A	0.99981	0.03951	1251.8	0.242	0	0	0	ND	ND	ND
376	---	rs663367	17	136153451	A	G	0.99777	0.99649	81.231	464.11	706.66	0	0.25023	-0.079597	0.045747	0.081872
377	---	rs552148	17	136153481	T	C	0.9846	0.97189	85.345	472.52	694.13	0.001	0.25687	-0.073316	0.045666	0.10838

continued

continued 3

378	---	rs61696148	17	136153544	A	T	0.99658	0.98329	1026	216.96	9.047	0.001	0.093873	0.059061	0.069996	0.39879
379	---	rs141638449	17	136153797	C	T	0.99874	0.036203	1250.4	1.576	0	0	0.00062939	ND	ND	ND
380	---	rs651007	17	136153875	C	T	0.99996	0.9999	647.01	504.99	100	0	0.28155	-0.46236	0.044194	1.2925e-25
381	---	rs11244061	17	136153981	C	T	0.99201	0.57166	1234.7	17.302	0	0	0.0069097	-0.47242	0.30302	0.11898
382	---	rs187861744	17	136154097	G	C	0.99827	0.51738	1248.4	3.554	0	0	0.0014193	4.8627	1.0473	0.0001943
383	---	rs579459	17	136154168	T	C	0.99997	0.99993	647.01	504.99	100	0	0.28155	-0.46236	0.044194	1.2893e-25
384	---	rs9411488	17	136154246	G	T	0.99751	0.99417	805.67	381.75	64.577	0	0.20404	0.040535	0.048138	0.39975
385	---	rs649129	17	136154304	C	T	0.99998	0.99995	647.01	504.99	100.01	0	0.28155	-0.46236	0.044193	1.2891e-25
386	---	rs10751504	17	136154433	G	C	0.9682	0.93236	708.84	473.58	69.535	0.044	0.24468	0.38052	0.049077	8.9352e-15
387	---	chr9:136154623:D	17	136154623	TC	T	0.99812	0.12397	1249.6	2.357	0	0	0.00094129	ND	ND	ND
388	---	chr9:136154626:D	17	136154626	CA	C	0.99812	0.12397	1249.6	2.357	0	0	0.00094129	ND	ND	ND
389	---	rs147375567	17	136154866	A	G	0.99898	0.50891	1249.9	2.111	0	0	0.00084305	-0.24873	1.0283	0.80887
390	rs495828	rs495828	17	136154867	G	T	1	1	647	505	100	0	0.28155	-0.46238	0.044194	1.283e-25
391	---	rs635634	17	136155000	C	T	0.99998	0.99995	647.01	504.98	100.01	0	0.28155	-0.46236	0.044193	1.288e-25
392	---	rs12683493	17	136155063	C	T	0.99649	0.98298	1026	216.93	9.046	0.003	0.093858	0.05928	0.07002	0.39721
393	---	rs150022816	17	136155093	G	T	1	1	1252	0	0	0	0	ND	ND	ND
394	---	rs13298002	17	136155127	G	A	0.99811	0.12465	1249.6	2.365	0	0	0.00094449	ND	ND	ND
395	rs500428	rs500428	17	136155343	A	G	1	1	90	474	688	0	0.26118	-0.086892	0.04489	0.052908
396	---	rs7030248	17	136155359	G	A	0.99743	0.99482	692.55	484.83	74.628	0	0.25323	0.38089	0.046505	2.6058e-16
397	---	rs9411490	17	136155414	A	G	0.99744	0.99405	805.68	381.77	64.549	0.003	0.20402	0.040483	0.048145	0.40043
398	---	rs633862	17	136155444	C	T	0.99743	0.99608	279.07	606.76	366.17	0.002	0.46522	-0.094595	0.039506	0.016647
399	---	rs502361	17	136155589	C	G	0.99994	0.99985	89.994	473.97	688.04	0	0.26116	-0.086917	0.044892	0.052853
400	---	rs9411491	17	136156015	T	C	0.99715	0.99328	805.67	381.78	64.553	0.004	0.20403	0.040355	0.048159	0.40206
401	---	rs55988407	17	136156064	G	A	0.9809	0.47025	1218	33.835	0.149	0.003	0.013631	0.44056	0.24649	0.073883
402	---	rs9411381	17	136156067	T	C	0.99683	0.99246	805.65	381.78	64.556	0.017	0.20403	0.040234	0.048177	0.40365
403	---	rs78590974	17	136156230	C	T	0.98471	0.47114	1226.6	25.202	0.241	0.002	0.010257	2.0613	0.3907	1.3213e-07
404	---	rs142447342	17	136156244	G	A	1	1	1252	0	0	0	0	ND	ND	ND
405	---	rs140502769	17	136156498	G	A	0.99999	0.0094373	1252	0.018	0	0	0	ND	ND	ND
406	---	rs144306320	17	136156542	G	T	0.999	0.013524	1250.7	1.255	0	0.001	0.0005012	ND	ND	ND
407	---	rs189611925	17	136156672	G	T	0.9998	0.016521	1251.8	0.246	0	0	0	ND	ND	ND
408	---	rs557317	17	136157037	C	A	0.98391	0.9689	97.661	488.34	665.97	0.026	0.27303	-0.09539	0.045036	0.034167
409	---	rs558240	17	136157133	G	A	0.99025	0.97154	895.23	333.18	23.573	0.012	0.15189	-0.10138	0.057695	0.078881
410	---	rs138026250	17	136157138	C	A	0.99982	0.13924	1251.8	0.224	0	0	0	ND	ND	ND
411	---	rs146149084	17	136157335	A	G	0.99998	0.011513	1252	0.023	0	0	0	ND	ND	ND
412	---	rs181316261	17	136157449	C	T	0.99919	0.14459	1251	1.009	0	0	0.00040296	ND	ND	ND
413	---	rs7867220	17	136157520	C	T	0.99949	0.11798	1251.4	0.64	0	0	0.00025559	ND	ND	ND
414	---	rs183796796	17	136157596	C	T	0.99988	0.032044	1251.8	0.154	0	0	0	ND	ND	ND
415	---	rs142673851	17	136157644	C	T	0.9994	0.14465	1251.3	0.746	0	0	0.00029792	ND	ND	ND
416	---	rs62574607	17	136157654	T	C	0.99069	0.87367	1181.9	67.059	3.032	0.002	0.029203	0.17883	0.12453	0.15097
417	---	rs7867359	17	136157656	C	G	0.99945	0.12814	1251.3	0.687	0	0	0.00027436	ND	ND	ND
418	---	rs7853492	17	136157785	A	T	0.99945	0.14681	1251.3	0.69	0	0	0.00027556	ND	ND	ND
419	---	rs7867739	17	136157924	C	T	0.99942	0.15659	1251.3	0.726	0	0	0.00028994	ND	ND	ND
420	---	rs7867864	17	136158008	C	G	0.99936	0.15691	1251.2	0.8	0	0	0.00031949	ND	ND	ND
421	---	rs141952207	17	136158026	G	C	0.99996	0.008981	1252	0.048	0	0	0	ND	ND	ND
422	---	rs149863406	17	136158155	C	T	0.99918	0.013745	1251	1.032	0	0	0.00041214	ND	ND	ND
423	---	rs28476494	17	136158288	T	C	0.99928	0.18331	1251.1	0.9	0	0	0.00035942	ND	ND	ND
424	---	rs7860182	17	136158327	T	A	0.98662	0.97544	98.485	491.58	661.91	0.02	0.27499	-0.090135	0.044889	0.044647
425	---	rs28368280	17	136158400	C	G	0.99925	0.1935	1251.1	0.942	0	0	0.0003762	ND	ND	ND
426	---	rs184855103	17	136158490	C	T	0.99998	0.0090575	1252	0.029	0	0	0	ND	ND	ND
427	---	rs1927311	17	136158544	T	C	0.9874	0.97617	104.32	494.88	652.79	0.01	0.28096	-0.094887	0.044506	0.033006
428	---	rs115071869	17	136158862	G	A	0.99911	0.23694	1250.9	1.112	0	0	0.00044409	ND	ND	ND
429	---	rs507480	17	136158964	A	G	0.99937	0.989	3	59.69	1189.3	0.001	0.026234	-0.13081	0.12101	0.27972
430	---	rs507567	17	136158998	A	G	0.99705	0.38212	0.015	5.06	1246.9	0.003	0.0020328	-0.51462	0.57591	0.37155
431	---	rs509482	17	136159240	G	A	0.98604	0.96899	724.2	454.3	73.468	0.029	0.24012	0.067143	0.047084	0.15386
432	---	rs62574608	17	136159266	A	G	0.92354	0.50729	1110.6	135.9	5.479	0.02	0.058652	0.22538	0.11535	0.050713
433	---	chr9:136159268:D	17	136159268	AAGGCC	A	0.92738	0.62775	1063.2	181.65	7.148	0.013	0.078252	0.0096289	0.093572	0.91804
434	---	rs146582653	17	136159450	G	C	0.99949	0.4148	1251.3	0.684	0	0	0.00027316	ND	ND	ND
435	---	rs67318122	17	136159538	A	G	0.9957	0.27916	1246.6	5.365	0.013	0	0.002153	3.5202	0.73093	1.0627e-06
436	---	rs512179	17	136159540	C	G	0.99989	0.9997	703.95	467.03	81.018	0.001	0.25122	0.077437	0.045708	0.090234
437	---	rs512887	17	136159579	G	A	0.9999	0.99973	703.96	467.03	81.016	0	0.25122	0.077438	0.045708	0.090232
438	---	rs535855	17	136159790	A	G	0.99996	0.99988	703.98	467.01	81.008	0	0.25121	0.077425	0.045705	0.090264
439	---	rs535993	17	136159843	A	G	0.99996	0.99989	703.98	467.01	81.007	0	0.25121	0.077422	0.045705	0.090276
440	---	chr9:136159892:I	17	136159892	T	TAA	0.90366	0.70227	949	287.24	15.747	0.016	0.12729	-0.05678	0.073102	0.43733
441	---	chr9:136159896:I	17	136159896	T	TAAAAGAAATAA	0.90474	0.70343	954.66	281.97	15.353	0.016	0.12487	-0.077352	0.073269	0.29109
442	---	rs184760764	17	136160131	G	A	0.999	0.023915	1250.7	1.254	0	0	0.0005008	ND	ND	ND
443	---	rs146174278	17	136160165	G	A	0.99934	0.062768	1251.2	0.822	0	0	0.00032827	ND	ND	ND
444	---	rs1752338	17	136160185	C	A	1	1	704	467	81	0	0.2512	0.077404	0.045704	0.090341
445	---	rs1633512	17	136160219	T	C	1	1	704	467	81	0	0.2512	0.077404	0.045704	0.090341
446	rs1752337	rs1752337	17	136160228	T	G	1	1	704	467	81	0	0.2512	0.077404	0.045704	0.090341
447	---	rs62574609	17	136160311	T	C	1	1	704	467	81	0	0.2512	0.077404	0.045704	0.090341
448	---	rs62574610	17	136160481	C	T	1	1	704	467	81	0	0.2512	0.077404	0.045704	0.090341
449	---	rs62574611	17	136160538	G	C	1	1	704	467	81	0	0.2512	0.077404	0.045704	0.090341
450	---	rs9411492	17	136160617	T	C	0.98128	0.97184	287.87	612.33	351.77	0.02	0.47448	-0.098358	0.040567	0.015325
451	---	rs565126	17	136160632	G	A	0.99638	0.9922	701.88	468.47	81.647	0.004	0.2523	0.07675	0.045854	0.094176
452	---	chr9:136160859:I	17	136160859	C	CA	0.90639	0.83416	110.21	546.6	595.11	0.084	0.30634	-0.10272	0.048515	0.034245
453	---	rs567898	17	136160931	A	G	1	1	704	467	81	0	0.2512	0.077404	0.045704	0.090341
454	---	rs568022	17	136160975	G	C	0.95943	0.90869	745.5	442.23	64.26	0.012	0.22794	0.07899	0.049758	0.11241
455	---	rs568975	17	136161074	G	A	1	1	704	467	81	0	0.2512	0.077404	0.045704	0.090341
456	---	rs9411382	17	136161274	T	C	0.99999	0.99999	81.005	467	704	0	0.2512	-0.077414	0.045704	0.090302
457	---	rs572651	17	136161469	C	T	0.99998	0.99994	704	466.97	81.027	0	0.25121	0.077346	0.045702	0.090573
458	---	rs572653	17	136161472	A	G	1	1	704	467	81	0	0.2512	0.077402	0.045704	0.090351
459	---	rs139740994	17	136161579	G	C	0.99976	0.030689	1251.7	0.296	0	0	0.00011821	ND	ND	ND
460	---	rs11244064	17	136161837	G	A	0.98501	0.96369	803.78	411.49	36.723	0.017	0.19367	0.02578	0.053656	0.63089
461	rs9411493	rs9411493	17	136161851	A	G	1	1	102	491	659	0	0.27756	-0.088516	0.04397	0.044103
462	---	chr9:136161928:D	17	136161928	TTTC	T	0.99708	0.95666	1186.6	62.174	3.211	0.004	0.027395	0.14677	0.12289	0.23233
463	---	rs176689	17	136162131	G	T	1	1	704	467	81	0	0.2512	0.077402	0.045704	0.090351
464	---	rs68032997	17	136162255	T	C	0.97739	0.92826	937.61	289.88	24.48	0.03	0.13532	0.08326	0.060218	0.16677
465	---	rs176690	17	136162764	C	T	0.99896	0.99794	705.11	466.17	80.716	0.001	0.25064	0.077661	0.045858	0.090362
466	---	rs117653144	17	136162783	G	A	0.99704	0.96021	1197.7	51.31	3	0.001	0.022887	0.15594	0.13015	0.23086
467	---	rs176691	17	136163306	G	A	0.98172	0.95706	798.88	415.98	37.115	0.02	0.19578	0.033599	0.054045	0.53416
468	---	rs176692	17	136163329	A	G	0.98149	0.95645	799.21	415.7	37.067	0.017	0.19562	0.033273	0.054077	0.53837
469	---	rs10901256	17	136163447	T	C	0.97309	0.95884	317.58	609.39	325	0.034	0.49704	-0.059573	0.040579	0.14209
470	---	rs10901257	17	136163570	G	A	0.98239	0.97356	286.85	612.97	352.16	0.022	0.47392	-0.096162	0.040535	0.017678
471	---	rs176693	17	136164001	C	T	1	0.99999	704	467	81	0	0.2512	0.0774	0.045704	0.090359
472	---	rs150942415	17	136164037	G	A	0.99995	0.012319	1251.9	0.064	0	0	0	ND	ND	ND
473	---	rs140667476	17	136164095	G	T	0.99989	0.01535	1251.9	0.134	0	0	0	ND	ND	ND
474	---	rs28844968	17	136164427	A	G	0.96493	0.8298	1053.2	192.58	6.205	0.009	0.081864	-0.0153	0.080769	0.84976
475	---	rs176694	17	136164593	T	G	0.99808	0.28579	1249.4	2.572	0	0	0.0010272	ND	ND	ND
476	---	rs176695	17	136164639	A	G	1	0.99999	704	467	81	0	0.2512	0.0774	0.045704	0.090361
477	---	rs176696	17	136164655	C	T	0.99976	0.9994	102.08	491.12	658.8	0.002	0.27767	-0.088758	0.043989	0.043619
478	---	rs13301309	17	136164836	C	A	0.99571	0.2919	1246.6	5.36	0.013	0	0.002151	2.9113	0.66463	5.7555e-05
479	---	rs191901590	17	136164922	T	G	1	1	1252	0	0	0	0	ND	ND	ND
480	---	rs176697	17	136165211	T	C	0.99859	0.99719	101.56	490.13	660.31	0.005	0.27686	-0.08956	0.044031	0.041951
481	---	rs193010087	17	136165284	C	T	0.9993	0.14647	1251.1	0.871	0	0	0.00034784	ND	ND	ND
482	---	rs2519836	17	136165347	T	C	1	0.0009996	0	0.001	1252	0	0	ND	ND	ND
483	---	rs182507498	17	136165348	G	A	0.99422	0.22194	1243.9	8.078	0	0.001	0.003226	-0.26532	0.64619	0.68137
484	---	rs176698	17	136165371	A	C	0.99999	0.99999	704	467	81	0	0.2512	0.077398	0.045704	0.090367
485	---	rs145834222	17	136165436	C	A	0.99951	0.44019	1251.3	0.726	0	0	0.00028994	ND	ND	ND
486	---	rs187543752	17	136165446	C	T	1	1	1252	0	0	0	0	ND	ND	ND
487	---	rs7020432	17	136165694	G	A	0.99559	0.28675	1246.5	5.505	0.014	0	0.0022097	3.4798	0.72557	1.1803e-06
488	rs7025162	rs7025162	17	136166346	C	T	1	1	444	576	232	0	0.41534	-0.28097	0.039371	9.5694e-13

* id '---' indicate that these variants were extraporated.

Table S3. Associations of ABO gene in 9q32 with P-LIP or ACE levels after adjustment by smoking or Box-Cox transformation of trait values

				P-LIP						ACE					
SNPs		minor		adjusted by alcohol intake			Box-Cox transformation***			adjusted by smoking			Box-Cox transformation***		
(position)	stage*	allele	MAF	beta**	SE	p value	beta**	SE	p value	beta**	SE	p value	beta**	SE	p value
rs4363269	1st	G	0.287	0.0849	0.500	> 1e-7	0.00272	0.003	> 1e-7	-2.01	0.218	9.85e-20	-1.12	0.125	1.20e-18
(136123840)	2nd	G	0.269	-0.220	0.511	> 1e-7	0.000446	0.003	> 1e-7	-1.99	0.192	1.91e-24	-1.14	0.106	3.56e-26
rs8176749	1st	A	0.161	4.18	0.601	5.55e-12	0.0336	0.004	1.85e-16	2.57	0.266	2.67e-21	1.46	0.153	5.77e-21
(136131188)	2nd	A	0.188	5.12	0.576	1.61e-18	0.0396	0.004	6.45e-27	2.13	0.219	7.15e-22	1.28	0.123	1.03e-24
rs8176746	1st	A	0.167	4.16	0.593	3.75e-12	0.0476	0.004	4.35e-17	2.58	0.261	3.22e-22	1.47	0.15	7.39e-22
(136131322)	2nd	A	0.198	5.42	0.562	2.04e-21	0.0580	0.004	3.10e-30	2.11	0.214	2.74e-22	1.26	0.12	8.67e-25
rs2073824	1st	G	0.496	1.29	0.447	> 1e-7	0.00990	0.004	> 1e-7	1.17	0.199	6.25e-9	0.660	0.114	8.21e-9
(136132633)	2nd	A	0.475	-1.82	0.462	> 1e-7	-0.0127	0.003	> 1e-7	-1.17	0.176	4.00e-11	-0.622	0.099	3.68e-10
rs657152	1st	A	0.440	2.75	0.440	5.63e-10	0.0225	0.003	4.97e-14	-0.593	0.201	> 1e-7	-0.324	0.115	> 1e-7
(136139265)	2nd	A	0.454	3.38	0.455	1.81e-13	0.0276	0.003	2.47e-21	-0.685	0.178	> 1e-7	-0.417	0.099	> 1e-7
rs500498	1st	A	0.455	-2.71	0.438	8.70e-10	-0.0215	0.003	3.56e-13	0.371	0.2	> 1e-7	0.228	0.114	> 1e-7
(136148647)	2nd	A	0.445	-3.01	0.456	5.77e-11	-0.0247	0.003	2.28e-17	0.428	0.179	> 1e-7	0.260	0.099	> 1e-7
rs505922	1st	G	0.450	2.62	0.433	1.92e-9	0.0220	0.003	8.64e-14	-0.503	0.199	> 1e-7	-0.274	0.114	> 1e-7
(136149229)	2nd	G	0.458	3.57	0.452	4.97e-15	0.0294	0.003	3.02e-24	-0.630	0.178	> 1e-7	-0.389	0.099	> 1e-7
rs495828	1st	A	0.281	0.536	0.499	> 1e-7	0.00473	0.003	> 1e-7	-2.34	0.215	2.14e-26	-1.31	0.123	2.30e-25
(136154867)	2nd	A	0.264	-0.0315	0.509	> 1e-7	0.00214	0.003	> 1e-7	-2.37	0.188	9.84e-35	-1.39	0.104	1.80e-38
rs7025162	1st	A	0.415	-0.834	0.445	> 1e-7	-0.00565	0.003	> 1e-7	-1.50	0.197	5.83e-14	-0.810	0.113	1.11e-12
(136166346)	2nd	A	0.398	-0.667	0.467	> 1e-7	-0.00632	0.003	> 1e-7	-1.27	0.178	1.52e-12	-0.738	0.099	1.29e-13

* 1st population from the Takahata cohort, 2nd from the Yamagata cohort

** Regression coefficient

*** Optimal lambda value in Box-Cox transformation for P-LIP is -0.4 for all except rs8176746 that has -0.3, whereas that for ACE is 0.8 for all SNPs.

Table S4. Associations of ACE1 in 17q23.3 with ACE level after adjustment by smoking or Box-Cox transformation of trait values

				ACE					
				adjusted by smoking			Box-Cox transformation****		
SNPs (position)	stage*	minor allele	MAF	beta**	SE	p value	beta**	SE	p value
rs4459609	1st	C	0.371	3.06	0.192	3.91e-52	1.82	0.110	3.87e-56
(61548948)	2nd	C	0.356	2.48	0.177	8.81e-42	1.46	0.098	1.28e-47
rs4309	1st	G	0.426	3.31	0.181	1.03e-65	1.95	0.104	3.83e-69
(61559923)	2nd	G	0.409	3.27	0.163	1.52e-76	1.87	0.091	7.51e-85
rs4311	1st	A	0.349	3.25	0.189	5.08e-59	1.91	0.109	4.21e-62
(61560763)	2nd	A	0.346	2.54	0.180	2.99e-42	1.53	0.099	2.80e-50
rs4329	1st	A	0.358	3.25	0.189	2.10e-59	1.92	0.108	2.45e-63
(61563458)	2nd	A	0.342	2.64	0.176	1.00e-47	1.58	0.097	3.11e-55
rs4343	1st	G	0.360	3.23	0.189	9.54e-59	1.91	0.108	1.09e-62
(61566031)	2nd	G	0.342	2.65	0.176	6.93e-48	1.58	0.097	2.03e-55
rs4353	1st	A	0.440	3.91	0.173	4.09e-94	2.32	0.098	6.72e-102
(61570422)	2nd	A	0.422	3.51	0.158	1.07e-94	2.04	0.088	4.47e-104
rs4362	1st	A	0.442	3.94	0.173	1.55e-95	2.33	0.098	3.43e-103
(61573761)	2nd	A	0.426	3.54	0.157	8.97e-97	2.05	0.087	5.77e-106
rs4461142	1st	A	0.434	2.00	0.194	6.56e-24	1.16	0.111	7.77e-25
(61578048)	2nd	A	0.433	1.87	0.170	6.41e-27	1.09	0.095	5.04e-29

* 1st population from the Takahata cohort, 2nd from the Yamagata cohort

** Regression coefficient

*** Coefficient t-statistic

**** Optimal lambda value for Box-Cox transformation is 0.8 for all SNPs.

Table S5. Associations of ABO gene in 9q32 with P-LIP or ACE levels after adjustment or subgroup by treatment

SNPs	P-LIP								ACE							
	adjusted by treatment history*				subgroup of notreatment				adjusted by treatment history*				subgroup of notreatment			
(position)	stage**	beta***	SE	p value	stage**	beta***	SE	p value	stage**	beta***	SE	p value	stage**	beta***	SE	p value
rs4363269 (136123840)	1st<br>2nd	-0.0325<br>-0.343	0.481<br>0.564	> 1e-7<br>> 1e-7	comb	0.525	0.443	> 1e-7	1st<br>2nd	-1.96<br>-1.94	0.212<br>0.194	9.62e-20<br>6.18e-23	comb	-1.89	0.160	3.07e-31
rs8176749 (136131188)	1st<br>2nd	4.40<br>5.40	0.575<br>0.635	3.92e-14<br>4.53e-17	comb	4.04	0.524	2.15e-14	1st<br>2nd	2.52<br>2.35	0.258<br>0.223	1.24e-21<br>4.14e-25	comb	2.30	0.192	9.06e-32
rs8176746 (136131322)	1st<br>2nd	4.37<br>5.69	0.568<br>0.620	2.79e-14<br>1.41e-19	comb	4.17	0.516	1.32e-15	1st<br>2nd	2.53<br>2.30	0.254<br>0.218	1.83e-22<br>3.23e-25	comb	2.32	0.189	3.00e-33
rs657152 (136139265)	1st<br>2nd	2.65<br>3.49	0.424<br>0.506	5.38e-10<br>7.98e-12	comb	3.01	0.401	7.37e-14	1st<br>2nd	-0.550<br>-0.651	0.195<br>0.182	> 1e-7<br>> 1e-7	comb	-0.680	0.151	> 1e-7
rs500498 (136148647)	1st<br>2nd	-2.44<br>-3.02	0.421<br>0.507	8.35e-9<br>3.16e-9	comb	-2.56	0.399	1.71e-10	1st<br>2nd	0.362<br>0.376	0.194<br>0.182	> 1e-7<br>> 1e-7	comb	0.386	0.151	> 1e-7
rs505922 (136149229)	1st<br>2nd	2.53<br>3.64	0.419<br>0.501	2.22e-9<br>5.85e-13	comb	3.02	0.394	3.25e-14	1st<br>2nd	-0.454<br>-0.597	0.193<br>0.180	> 1e-7<br>> 1e-7	comb	-0.598	0.149	> 1e-7
rs495828 (136154867)	1st<br>2nd	0.250<br>-0.182	0.481<br>0.562	> 1e-7<br>> 1e-7	comb	0.640	0.446	> 1e-7	1st<br>2nd	-2.25<br>-2.38	0.209<br>0.190	7.13e-26<br>2.07e-34	comb	-2.32	0.157	2.23e-46
rs7025162 (136166346)	1st<br>2nd	-0.806<br>-0.678	0.428<br>0.510	> 1e-7<br>> 1e-7	comb	-0.230	0.402	> 1e-7	1st<br>2nd	-1.41<br>-1.29	0.191<br>0.178	2.85e-13<br>7.02e-13	comb	-1.22	0.148	2.12e-16

* Treatment history is coded by 0 for untreated, 1 for treated or NA for not available.

** 1st population from the Takahata cohort and 2nd from the Yamagata cohort, whereas comb is combined populations of certainly untreated individuals in both cohorts.

*** Regression coefficient

Table S6. Associations of ACE1 gene in 17q23.2 with ACE level after adjustment or subgroup by treatment

SNPs	ACE							
	adjusted by treatment history*				subgroup of notreatment			
(position)	stage**	beta***	SE	p value	stage**	beta***	SE	p value
rs4459609 (61548948)	1st<br>2nd	3.11<br>2.52	0.210<br>0.179	5.22e-57<br>2.08e-42	comb	2.86	0.148	7.45e-76
rs4309 (61559923)	1st<br>2nd	3.33<br>3.30	0.195<br>0.163	1.91e-70<br>2.25e-80	comb	3.30	0.135	8.57e-114
rs4311 (61560763)	1st<br>2nd	3.29<br>2.62	0.205<br>0.181	5.39e-64<br>1.78e-44	comb	2.94	0.149	1.50e-78
rs4329 (61563458)	1st<br>2nd	3.30<br>2.70	0.204<br>0.177	7.35e-65<br>7.42e-49	comb	3.02	0.146	1.00e-85
rs4343 (61566031)	1st<br>2nd	3.28<br>2.71	0.204<br>0.177	3.13e-64<br>2.88e-49	comb	3.02	0.146	1.27e-85
rs4353 (61570422)	1st<br>2nd	3.99<br>3.60	0.180<br>0.159	2.32e-105<br>1.54e-97	comb	3.78	0.128	1.49e-155
rs4362 (61573761)	1st<br>2nd	4.01<br>3.62	0.179<br>0.158	1.48e-106<br>1.51e-99	comb	3.77	0.128	6.96e-156
rs4461142 (61578048)	1st<br>2nd	2.03<br>1.97	0.209<br>0.172	4.40e-26<br>4.27e-29	comb	1.89	0.145	7.80e-37

* Treatment history is coded by 0 for untreated, 1 for treated or NA for not available.
** 1st population from the Takahata cohort and 2nd from the Yamagata cohort, whereas comb is combined populations of certainly untreated individuals in both cohorts.

*** Regression coefficient
**** Coefficient t-statistic

Table S7. SNPs associated with P-LIP and/or ACE levels at a genome-wide significance level

locus (chr)	SNPs (build135)	position (GRCh37/hg19)	starnd	major allele	minor allele	class: genome	class: functional	residue change	note
ABO	rs4363269	136123840	+	A	G	genomic	3' downstream ~6.7kb		
(9q32)	rs8176749	136131188	-	G	A	coding	synonymous	Leu310Leu	
	rs8176746	136131322	-	C	A	coding	nonsynonymous	Leu266Met	Allele A is associated with B group
	rs2073824	136132633	+	A	G	intron			
	rs657152	136139265	+	C	A	intron			
	rs500498	136148647	-	G	A	intron			
	rs505922	136149229	-	A	G	intron			Allele A is associated with O group
	rs495828	136154867	-	C	A	genomic	5' upstream ~4.2kb		
	rs7025162	136166346	-	G	A	genomic	5' upstream ~15.7kb		
ACE1	rs4459609	61548948	+	A	C	genomic	5' upstream ~5.5kb		
(17q23.3)	rs4309	61559923	-	A	G	coding	synonymous	Pro405Pro	
	rs4311	61560763	-	G	A	intron			
	rs4329	61563458	+	G	A	intron			
	rs4343	61566031	+	A	G	intron			
	rs4353	61570422	+	G	A	intron			
	rs4362	61573761	-	G	A	coding	synonymous	Phe555Phe	
	rs4461142	61578048	-	G	A	genomic	3' downstream ~2.3kb		

Table S8. Associations of ABO gene in 9q32 with P-LIP and ACE in combined population

SNPs	minor		P-LIP			ACE		
(position)	allele	MAF	beta*	T**	p value	beta*	T**	p value
rs4363269	G	0.276	-0.115	-0.321	> 1e-7	-1.94	-14.1	1.87e-43
rs8176749	A	0.177	4.78	11.6	3.02e-30	2.29	14.0	4.08e-43
rs8176746	A	0.186	4.94	12.2	2.01e-33	-1.07	-8.40	7.06e-17
rs657152	A	0.448	3.05	9.61	1.50e-21	0.417	3.26	> 1e-7
rs500498	A	0.449	-2.76	-8.71	5.16e-18	-0.581	-4.57	> 1e-7
rs505922	G	0.455	3.11	9.88	1.18e-22	-2.31	-17.0	> 1e-7
rs495828	A	0.271	0.101	0.282	> 1e-7	-2.27	-10.8	8.24e-62
rs7025162	A	0.404	-0.715	-2.21	> 1e-7	-1.31	-10.3	1.65e-24

* Regression coefficient
** Coefficient t-statistic

Table S9. Associations of ACE gene in 17q23.2 with ACE in combined population

SNPs (position)	minor allele	MAF	ACE beta*	ACE SE	ACE p value
rs4459609	G	0.276	2.765	0.125	9.462e-101
rs4309	A	0.177	3.27	0.116	5.659e-154
rs4311	A	0.186	2.891	0.126	4.389e-108
rs4329	A	0.448	2.945	0.123	3.04e-115
rs4343	A	0.449	2.939	0.123	6.647e-115
rs4353	G	0.455	3.702	0.112	4.446e-205
rs4362	A	0.271	3.726	0.111	1.527e-208
rs4461142	A	0.404	1.923	0.123	8.542e-053

* Regression coefficient
** Coefficient t-statistic

Table S10. Sample numbers removed by each quality control filter

Initial sample size	1639
Individual missing data rate (imiss) > 0.05	16
Individual heterozygosity > 0.35	6
Cryptic relatedness: likely 1st - 3rd relatives*	314
Population outliers (PCA by EIGENSTRAT)	51
Final sample size	1252

*eliminating sample with higher imiss from each pair.

Text S1. Organization and all contributors list of Yamagata University Genomic Cohort Consortium

Steering Committee: Takamasa KAYAMA[1] (Chair), Hidetoshi YAMASHITA[2] (Deputy chair), Akira FUKAO[3,4], Isao KUBOTA[5,6], Takeo KATO[7,8], Chifumi KITANAKA[9,10], Shinya SATO[1,5], Yoshiyuki UENO[11]

Respiratory, Cardiovascular and Renal Diseases & Related Traits: Isao KUBOTA[5,6], Shinya SATO[1,5], Tsuneo KONTA[6], Yoko SHIBATA[6], Tetsu WATANABE[6], Shuichi ABE[6], Takuya MIYAMOTO[6], Sumito INOUE[6], Takehiko MIYASHITA[6], Kazunobu ICHIKAWA[6], Tetsuro SHISHIDO[6], Takanori ARIMOTO[6], Hiroki TAKAHASHI[6], Satoshi NISHIYAMA[6] and Ami IKEDA[6]

Metabolic and Neurodegenerative Diseases & Related Traits: Takeo KATO[7,8], Makoto DAIMON[8], Toru KAWANAMI[8], Manabu WADA[8], Shigeki ARAWAKA[8], Hidetoshi OIZUMI[8], Katsuro KUROKAWA[8], Shingi SUSA[8], Yuichi KATOU[8], Wataru KAMEDA[8], Shingo KOYAMA[8], Shigeru KARASAWA[8], Chifumi ISEKI[8] and Yoshimi TAKAHASHI[8]

Cancer & Related Traits: Chifumi KITANAKA[9,10], Yoshiyuki UENO[11]

Gastrointestinal, Hepatic and Pancreatic Diseases & Related Traits: Yoshiyuki UENO[11], Sumio KAWATA[11]*, Takafumi SAITO[11], Naohiko MAKINO[11,12], Kazuo OKUMOTO[11], Hiroaki HAGA[11], Takeshi SATO[11], Chikako SATO[11], Hisayoshi WATANABE[11], Yuko NISHISE[11], Rika ISHII[11], Akiko MATSUDA[12] and Tomohiro TOZAWA[11,12]

Eye Diseases & Related Traits: Hidetoshi YAMASHITA[2] and Kei HONMA[2]

Cohort Establishment: Akira FUKAO[3,4], Hiroto NARIMATSU[3], Kyoko SHIBATA[3], Akiko MIURA[3], Rina INOUE[3], Ai NUMAZAWA[3], Kahori KUDO[3], Yoko AITA[3], Noriko UMEZAWA[3], Yuko SAITO[3], Yumi TAKAHASHI[3], Yuka SUZUKI[3], Katsumi OTANI[4], Atsushi HOZAWA[4], Li SHAO[4], Masatsugu ORUI[13], Atsuko KOBAYASHI[14], Yuka KANOYA[14], Takiko HOSOYA[14], Ikuko SUZUKI[14], Mariko OTAKE[14], Yuko MORIKAGI[14], Akiko SEKIMATA[14], Manami HIRAKA[14], Yumi MATSUDA[14], Chika SATO[14], Yoko TAKEDA[14], Yoko MATSUNAMI[14], Tatsuya HORIE[14], Shiho SATO[14], Mizue INOUE[14] and Kaoru BABA[14]

Genetic, Genomic and Statistical Analyses: Gen TAMIYA[15], Masao UEKI[15] and Tomohiro NAKAMURA[15]

Genotyping: Gen TAMIYA[15], Jamiyansuren JAMBALDORJ[16] and Satoko ARAKI[16]

DNA Extraction and Biobanking: Osamu NAKAJIMA[17,18]

Database Construction: Kazuei TAKAHASHI[19] and Kazuo GOTO[19]

Patent Control and Commercialization: Kimishige ISHIZAKA[20]

1. Yamagata University, Graduate School of Medicine, Department of Neurosurgery,
2. Department of Ophthalmology,
3. Advanced Molecular Epidemiology Research Institute (AMERI), Cohort Management Unit,
4. Department of Public Health,
5. AMERI, Respiratory and Cardiovascular Diseases Research Center,
6. Department of Cardiology, Pulmonology, and Nephrology,
7. AMERI, Metabolic and Degenerative Diseases Research Center,
8. Department of Neurology, Hematology, Metabolism, Endocrinology and Diabetology,
9. AMERI, Oncology Research Center,
10. Department of Molecular Cancer Science,
11. Department of Gastroenterology,
12. Division of Endoscopy, Yamagata University Hospital,
13. Yamagata Prefectural Tsuruoka Hospital,
14. School of Nursing,
15. AMERI, Genomic Information Analysis Unit,
16. AMERI, Shared Laboratory,
17. AMERI, Specimen Management Unit,
18. Research Laboratory for Molecular Genetics,
19. AMERI, Data Management Unit,
20. COME center.

* present address: Hyogo Prefectural Nishinomiya Hospital.
